# Annotated checklist of fishes from Monterey Bay National Marine Sanctuary with notes on extralimital species

**DOI:** 10.3897/zookeys.887.38024

**Published:** 2019-11-07

**Authors:** Erica J. Burton, Robert N. Lea

**Affiliations:** 1 Monterey Bay National Marine Sanctuary, National Ocean Service, National Oceanic and Atmospheric Administration, 99 Pacific Street, Building 455A, Monterey, California 93940, USA National Ocean Service, National Oceanic and Atmospheric Administration Monterey United States of America; 2 Department of Ichthyology, California Academy of Sciences, Golden Gate Park, 55 Music Concourse Drive, San Francisco, California 94118, USA Department of Ichthyology, California Academy of Sciences San Francisco United States of America; 3 Section of Ichthyology, Natural History Museum of Los Angeles County, 900 Exposition Boulevard, Los Angeles, California, 90007, USA Section of Ichthyology, Natural History Museum of Los Angeles County Los Angeles United States of America

**Keywords:** central California, cold-water event, Davidson Seamount, Elkhorn Slough, El Niño/Southern Oscillation, extralimital, introduced species, La Niña, marine protected area, species inventory, taxonomy, warm-water event

## Abstract

Monterey Bay National Marine Sanctuary is a federal, marine protected area located off the central coast of California, USA. Understanding biodiversity, and how it is changing, is necessary to effectively manage the sanctuary. The large size of this sanctuary, which contains a variety of habitats and is influenced by several water masses, provides for a diverse fish fauna. The central California coast has a rich history of ichthyological research and surveys, contributing to a unique repository of information on fish diversity. Herein, we provide a checklist of fishes that occur within the sanctuary, including justification for each species. Ancillary record information including name-bearing type specimens, historic species, cold- or warm-water event species, introduced species, and occurrence at Davidson Seamount or Elkhorn Slough are also provided. This represents the first comprehensive annotated checklist of 507 fishes known to occur within the sanctuary. In addition, 18 species are considered to be extralimital. This annotated checklist of fishes can be used by those interested in zoogeography, marine protected areas, ichthyology, regional natural history, and sanctuary management.

## Introduction

**Figure 1.**
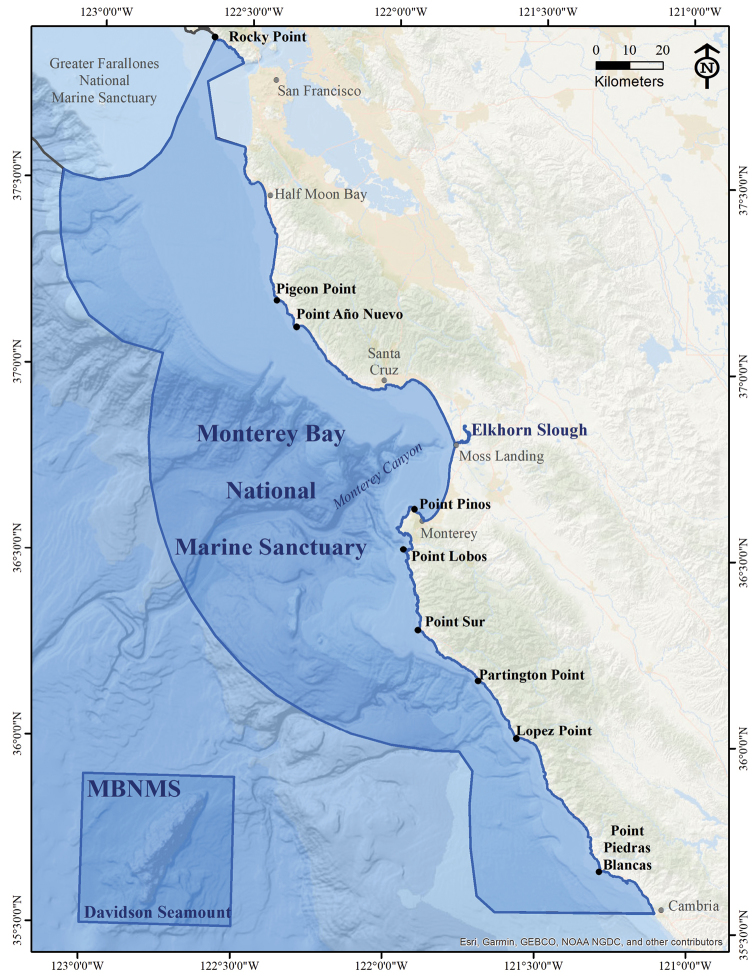
Monterey Bay National Marine Sanctuary. Monterey Bay National Marine Sanctuary (MBNMS) is a federal, marine protected area that spans from Rocky Point (Marin County, just north of the Golden Gate Bridge) to Cambria (San Luis Obispo County), California, USA; and includes Elkhorn Slough and the Davidson Seamount Management Zone. Points of interest (bold black font) and cities of interest (light gray font) are notated. Credit: Sophie De Beukelaer (Lynker Technologies LLC/MBNMS).

Monterey Bay National Marine Sanctuary (MBNMS or sanctuary) is a federal, marine protected area located off the central coast of California, USA (Fig. 1). Spanning the area from Rocky Point (Marin County) to Cambria (San Luis Obispo County), it encompasses 444 km of shoreline, 4,601 square nm of ocean, and extends from mean high tide to a seaward boundary that averages 48 km offshore. At its deepest point within the Davidson Seamount Management Zone, MBNMS reaches a depth of 3,875 m. The sanctuary contains a variety of habitats including estuaries (largest is Elkhorn Slough), sandy beaches, rocky shores, kelp forests, continental shelf and slope, deep submarine canyons (largest is Monterey Canyon), open ocean, and Davidson Seamount (Fig. 1). These habitats, individually and collectively, harbor an incredible variety of marine life.

It is an inconvenient truth that managing areas for ecosystem sustainability is impossible without knowledge of the species that compose it (Wilson 2014). Moreover, to demonstrate ecological significance or from simple curiosity the question is often asked, “How many species occur in Monterey Bay National Marine Sanctuary?” It may be specific to particular taxa, such as algae, invertebrates, fishes, seabirds and shorebirds, or marine mammals. The answer, however, is not absolute. Species distributions are dynamic (Lonhart 2009) shifting over time due to natural and/or anthropogenic mechanisms, or due to oceanic and atmospheric events (e.g., El Niño/Southern Oscillation, global change). In addition, species continue to be discovered and described from less sampled areas (e.g., deep sea).

The large size of MBNMS, influenced by several water masses and its proximity to Point Conception, provides for a diverse fish fauna. The central California marine fish fauna is influenced by water masses from the north, south, and west; and these water masses converge to create a major transition zone centered around Point Conception (south of MBNMS, Horn et al. 2006). Two distinctive fish faunas intermingle in this transition zone, a warm-temperate, southern component (San Diegan Province) and a cool-temperate, northern component (Oregonian Province). The fish fauna in the southern end of the sanctuary may at times be influenced by this transition zone, especially during warm-water events such as El Niño/Southern Oscillation. The Davidson Seamount lies well offshore (ca. 121 km due west of San Simeon, San Luis Obispo County), and the fish fauna there may be influenced by oceanic water masses to the west, such as the Subarctic-Transitional and Central Pacific water masses.

The central California coast has a rich history of ichthyological research and surveys, including: early biological expeditions to describe the California fish fauna (e.g., Pacific Expeditions of the US Fish Commission Steamer “Albatross”, Pacific Railroad Surveys); early institutions involved in specimen collections and subsequent study (e.g., California Academy of Sciences, Stanford University, California Department of Fish and Game, currently California Department of Fish and Wildlife, and Scripps Institution of Oceanography); and the more recent expansion of marine research institutions in central California. These early and recent surveys, preserved specimen collections, and scientific publications provide a wealth of information to create an inventory of fishes that occur within MBNMS.

A comprehensive inventory of fish species occurring within MBNMS is unavailable. Disparate lists are available for subsets of fishes, including commonly occurring species, fished species, and species within a particular habitat of MBNMS (e.g., Guerrero and Kvitek 1996, Monterey Bay Aquarium 1999, Starr et al. 2002, Yoklavich et al. 2002, Burton and Lundsten 2008, Burton et al. 2017, SIMoN 2019). We created a comprehensive annotated checklist of fishes occurring within MBNMS based on critical analyses of material from ichthyological collections at natural history museums, the literature, and visual records. The checklist presented here provides sources of basis (i.e., justification), occurrence of fishes during cold- or warm-water events, records of historically occurring fishes, reference to original species descriptions from within MBNMS, special places of occurrence (i.e., Elkhorn Slough, Davidson Seamount), and introduced species. Our goal was to compile a defensible checklist of fishes known to occur within the sanctuary.

## Materials and methods

We first generated a draft list of fishes that should occur within MBNMS based on regional guidebooks and checklists of fishes, including: Fitch and Lavenberg (1968), Miller and Lea (1972), Hubbs et al. (1979), Eschmeyer et al. (1983), Love et al. (2002), Yoklavich et al. (2002), Ebert (2003), Love et al. (2005), and Burton and Lundsten (2008). The majority of these guidebooks encompass broader regions (e.g., California, eastern North Pacific) than investigated here. From these lists, we examined basis within MBNMS for each species.

In an effort to provide defensible justification for each species, we considered the following sources as basis for inclusion, in order of importance: 1) museum specimen; 2) publication; and/or 3) expert-verified visual record. In addition, we followed a general rule for inclusion: the occurrence of any life history stage of the species within the current boundaries of MBNMS (including warm-water event, cold-water event, or historic record).

To determine a basis for inclusion (if any), we investigated ichthyological collections at natural history museum collections with online databases including California Academy of Sciences, Stanford University, Scripps Institution of Oceanography at University of California San Diego, Los Angeles County Museum of Natural History, Burke Museum at University of Washington, and the Smithsonian Institution’s National Museum of Natural History. In addition, we consulted the Moss Landing Marine Laboratories fish collection, which is primarily a teaching resource. Where records were few and/or questionable, we examined specimens that were readily accessible to confirm identification. If museum specimens were lacking, we then consulted publications, where primary sources of peer-reviewed scientific literature carried the most weight. Finally, if taxonomic experts could verify species from imagery (i.e., photos or video), we sparingly used these visual record(s) for basis. With technological advancements, video observations of deep-sea species are on the rise. Monterey Bay Aquarium Research Institute’s (MBARI) online Deep Sea Guide (Jacobson Stout et al. 2017) provides an excellent source of in situ imagery observations of regional species, particularly for rarely collected deep-sea species.

In addition, we conducted a midwater trawl survey during May 2015 aboard NOAA Ship “Bell M. Shimada”, which included the following areas of MBNMS: Davidson Seamount, Sur Ridge, Monterey Canyon, and off Monterey (unpublished data, Burton, Lea, and DeVogelaere). Juvenile and adult specimens were deposited at California Academy of Sciences (CAS), and larval specimens were stored as uncatalogued specimens at Southwest Fisheries Science Center (SWFSC), National Marine Fisheries Service (NMFS), La Jolla, California.

The checklist is organized in taxonomic order, following "Fishes of the World" (Nelson 2006), unless otherwise stated. Species names are alphabetized within family. Authority is included following current usage in "Catalog of Fishes" (Eschmeyer et al. 2017) and "Common and Scientific Names of Fishes" (Page et al. 2013), unless otherwise stated. If a species was originally described from within MBNMS boundaries, the original publication (therefore authority) is included in the references section (as basis). Official common names follow Page et al. (2013) or Hubbs et al. (1979), unless otherwise stated; and are capitalized following Page et al. (2013). Where no official common name was available, a generic common name (lowercase) was used, based on family name.

Terms used to describe species inclusion are defined below. Symbolic museum codes used in the checklist are listed in Table 1; full museum names and location information were sourced from Sabaj (2016). Other institution codes and acronyms are listed in Table 2.

**Table 1.** Symbol codes for natural history museum collections, including names of institutions and locations. Source: Sabaj (2016).

**Abbrev.**	**Name**	**Location**
ANSP	Academy of Natural Sciences of Drexel University	Philadelphia, Pennsylvania, USA
BMNH	Natural History Museum [formerly British Museum (Natural History)]	London, England, UK
CAS-ICH	California Academy of Sciences, Ichthyology Collection	San Francisco, California, USA
CAS-SU	Stanford University (collection now housed at California Academy of Sciences)	San Francisco, California, USA
FMNH	Field Museum of Natural History, Zoology Department	Chicago, Illinois, USA
KU	University of Kansas Biodiversity Institute	Lawrence, Kansas, USA
LACM	Natural History Museum of Los Angeles County	Los Angeles, California, USA
MBARI	Monterey Bay Aquarium Research Institute	Moss Landing, California, USA
MCZ	Museum of Comparative Zoology, Harvard University	Cambridge, Massachusetts, USA
MLMLF	Moss Landing Marine Laboratories Fishes	Moss Landing, California, USA
MNHN	Muséum national d’Histoire naturelle, Paris	Paris, France
NCSM	North Carolina Museum of Natural Sciences [formerly North Carolina State Museum]	Raleigh, North Carolina, USA
OS	Oregon State University, Department of Fisheries and Wildlife	Corvalis, Oregon, USA
OSUM	Ohio State University, Museum of Biological Diversity, Museum of Zoology	Columbus, Ohio, USA
SBMNH	Santa Barbara Museum of Natural History	Santa Barbara, California, USA
SIO	Scripps Institution of Oceanography at University of California San Diego	La Jolla, California, USA
UAM	University of Alaska Museum of the North, Fairbanks	Fairbanks, Alaska, USA
UCLA	University of California Los Angeles (now at SIO)	Los Angeles, California, USA
UCM	University of Colorado Museum of Natural History, Boulder, Colorado	Boulder, Colorado, USA
UF	University of Florida, Florida Museum of Natural History	Gainesville, Florida, USA
UMMZ	University of Michigan Museum of Zoology	Ann Arbor, Michigan, USA
USNM	National Museum of Natural History, Smithsonian Institution [formerly US National Museum]	Washington, DC, USA
UW	University of Washington	Seattle, Washington, USA
ZMB	Museum für Naturkunde [formerly Zoologischen Museum]	Berlin, Germany
ZMUB	Universitetet Bergen, Bergen Museum, Naturhistorie, Zoologiske, Vertebratsamlinger	Bergen, Norway

**Table 2.** List of institution codes (non-museum) and other acronyms.

**Acronym**	**Institution or definition**
CalCOFI	California Cooperative Oceanic Fisheries Investigations
CDFG	California Department of Fish and Game (now CDF Wildlife)
CSUMB	California State University Monterey Bay
ESNERR	Elkhorn Slough National Estuarine Research Reserve
IfAME	Institute for Applied Marine Ecology (at CSUMB)
MBA	Monterey Bay Aquarium
MBARI	Monterey Bay Aquarium Research Institute
MBNMS	Monterey Bay National Marine Sanctuary
MLML	Moss Landing Marine Laboratories
NOAA	National Oceanic and Atmospheric Administration
NMFS	National Marine Fisheries Service
SIMoN	Sanctuary Integrated Monitoring Network
SL	Standard Length
SWFSC	Southwest Fisheries Science Center (at NMFS)
TL	Total Length
UCLA	University of California, Los Angeles

### Basis categories and definitions

**Museum specimens**: Specimens were collected within MBNMS and catalogued at a natural history museum. Specimens listed do not necessarily represent all catalogued specimens occurring within MBNMS, but merely as examples to provide a basis for inclusion. We attempted to list three records (where available); and for those records to span a geographical range of occurrence within MBNMS, identified by known experts. Records are listed in alphabetical order, according to museum symbolic code (list of natural history museum collections and symbolic codes used for this checklist are provided in Table 1); type specimens are listed first. Collection location and name of who identified specimen are included. Identifier’s first initial, middle initial (if provided), and last name are provided; names are transcribed as provided and no assumptions are made to refine or match to others. Year of collection may be noted, especially if species occurred during an unusual oceanographic event. Type specimens (name-bearing and non-name-bearing) may be denoted (see ancillary record categories, below). Museum specimen(s) notated in checklist as follows: Museum code and catalog number (collection location, identifier).

**Publications**: Peer-reviewed information that provides a basis for occurrence within MBNMS. Other supporting publications may also be listed. Location observed is in parentheses. Year of collection may be noted, especially if species occurred during an unusual oceanographic event. Publication(s) notated in checklist as follows: Author (year) (location, additional information if available).

**Visual records**: Species observed with imagery (video or photo) and verified by expert or authority. Year of collection may be noted, especially if it occurred during an unusual oceanographic event. Visual record(s) are listed in checklist as institution code and dive number (observed location, identifier).

Several species occurring within MBNMS are anadromous (i.e., move up streams and rivers from the sea to spawn). Records of species occurring in fresh or brackish water (adjacent to MBNMS) are included where no marine records are available, because part of the life cycle requires movement between fresh and marine water bodies, thereby traversing MBNMS waters.

### Ancillary record categories and definitions

**Type specimens**: Name-bearing type specimens (holotype, neotype, syntype, or lectotype) collected within MBNMS. Type specimen(s) are listed in checklist as name-bearing type (museum code and catalog number).

**Historic specimens**: Specimen(s) collected many years before present, but not known from recent years; and not otherwise collected during unusual oceanographic events (i.e., warm or cold-water events).

**Warm-water events**: Specimen(s) collected during, or soon after, unusually warm-water events (e.g., warm phase of El Niño/Southern Oscillation, ENSO; El Niño), but otherwise unknown or rare during normal oceanographic conditions. Several references were consulted to determine the occurrence of warm-water events, including Wolter and Timlin (2011) (ENSO behavior since 1871), NOAA Earth System Research Laboratory (2019), and Null (2019); referencing the Multivariate ENSO Index (MEI), Oceanic Niño Index (ONI), and Niño 3.4 region.

**Cold-water events**: Specimen(s) collected during, or soon after, unusually cold-water events (e.g., cold phase of El Niño/Southern Oscillation: La Niña), but otherwise unknown or rare during normal oceanographic conditions. Several references were consulted to determine the occurrence of cold-water events, including Wolter and Timlin (2011) (ENSO behavior since 1871), NOAA Earth System Research Laboratory (2019), and Null (2019), referencing the Multivariate ENSO Index (MEI), Oceanic Niño Index (ONI), and Niño 3.4 region.

### Occurrence of note and definitions

**Davidson Seamount area**: Known to occur within the Davidson Seamount Management Zone of MBNMS (Fig. 1).

**Elkhorn Slough area**: Known to occur in Elkhorn Slough (within MBNMS, Fig. 1) or adjacent areas (i.e., Moss Landing Harbor, jetties).

**Introduced species**: Non-native to MBNMS, and either accidentally or deliberately transported to the area.

**Extralimital**: Species known to occur from areas to the north and south of MBNMS, but no basis could be found within MBNMS (yet likely to occur within MBNMS). Extralimital species are not considered part of the primary checklist, and are listed separately.

## Results and discussion

The annotated checklist includes 507 fish species, representing 325 genera and 148 families (Table 3). The number of taxa supported by specific sources of basis are as follows: museum specimens (465); publications (230); and visual records (44). Original descriptions of 57 species (type specimens, 11%) originated from within current MBNMS boundaries.

**Table 3.** Monterey Bay National Marine Sanctuary Fishes Checklist summary. Species are listed in taxonomic order, and include scientific name, authority, and common name. Categories to summarize basis and occurrence are provided, including: Basis for Inclusion (i.e., museum specimen, publication, visual record); Ancillary Record (i.e., type specimen, historic, warm-water event, cold-water event); and Occurrence of Note (i.e., Davidson Seamount area, Elkhorn Slough area, Introduced Species). Symbols denote the following: X (basis provided by); M (museum specimen); P (publication); V (visual record). Column subtotals are provided after line 507. See Methods for explanation of basis.

						Basis			Ancillary record				Occurrence of note		
	Scientific name				Common name	Museum specimen	Publication	Visual record	Type specimen	Historic	Warm-water event	Cold-water event	Davidson Seamount area	Elkhorn Slough area	Introduced species
	CLASS/Order/**Family**/*Species*														
	MYXINI														
		Myxiniformes													
			**Myxinidae (hagfishes)**												
1				*Eptatretusdeani* (Evermann & Goldsborough, 1907)	Black Hagfish	X									
2				*Eptatretusstoutii* (Lockington, 1878)	Pacific Hagfish	X								M	
	PETROMYZONTIDA														
		Petromyzontiformes													
			**Petromyzonidae (lampreys)**												
3				*Entosphenustridentatus* (Gairdner, 1836)	Pacific Lamprey	X									
	CHONDRICHTHYES														
		Chimaeriformes													
			**Chimaeridae (shortnose chimaeras)**												
4				*Hydrolaguscolliei* (Lay & Bennett, 1839)	Spotted Ratfish	X	X		X						
5				Hydrolaguscf.trolli Didier & Séret, 2002	Pointy-nosed Blue Chimaera		X	X					P/V		
		Heterodontiformes													
			**Heterodontidae (horn sharks)**												
6				*Heterodontusfrancisci* (Girard, 1855)	Horn Shark	X	X		X						
		Orectolobiformes													
			**Rhincodontidae (whale sharks)**												
7				*Rhincodontypus* Smith, 1828	Whale Shark		X	X				X			
		Lamniformes													
			**Alopiidae (thresher sharks)**												
8				*Alopiasvulpinus* (Bonnaterre, 1788)	Common Thresher Shark	X									
			**Cetorhinidae (basking sharks)**												
9				*Cetorhinusmaximus* (Gunnerus, 1765)	Basking Shark	X	X								
			**Lamnidae (mackerel sharks)**												
10				*Carcharodoncarcharias* (Linnaeus, 1758)	White Shark	X	X								
11				*Isurusoxyrinchus* Rafinesque, 1810	Shortfin Mako	X					X				
12				*Lamnaditropis* Hubbs & Follett, 1947	Salmon Shark	X	X								
		Carcharhiniformes													
			**Scyliorhinidae (cat sharks)**												
13				*Apristurusbrunneus* (Gilbert, 1892)	Brown Cat Shark	X									
14				*Apristuruskampae* Taylor, 1972	Longnose Cat Shark	X	X								
15				*Cephaloscylliumventriosum* (Garman, 1880)	Swell Shark	X									
16				*Parmaturusxaniurus* (Gilbert, 1892)	Filetail Cat Shark	X									
			**Triakidae (hound sharks)**												
17				*Galeorhinusgaleus* (Linnaeus, 1758)	Tope	X									
18				*Musteluscalifornicus* Gill, 1864	Gray Smoothhound	X	X							M/P	
19				*Mustelushenlei* (Gill, 1863)	Brown Smoothhound	X								M	
20				*Triakissemifasciata* Girard, 1855	Leopard Shark	X	X							M/P	
			**Carcharhinidae (requiem sharks)**												
21				*Prionaceglauca* (Linnaeus, 1758)	Blue Shark	X							P		
		Hexanchiformes													
			**Hexanchidae (cow sharks)**												
22				*Hexanchusgriseus* (Bonnaterre, 1788)	Bluntnose Sixgill Shark	X									
23				*Notorynchuscepedianus* (Péron, 1807)	Broadnose Sevengill Shark	X									
		Squaliformes													
			**Echinorhinidae (bramble sharks)**												
24				*Echinorhinuscookei* Pietschmann, 1928	Prickly Shark	X									
			**Squalidae (dogfish sharks)**												
25				*Squalussuckleyi* (Girard, 1855)	Pacific Spiny Dogfish	X									
			**Somniosidae (sleeper sharks)**												
26				*Somniosuspacificus* Bigelow & Schroeder, 1944	Pacific Sleeper Shark	X	X								
		Squatiniformes													
			**Squatinidae (angel sharks)**												
27				*Squatinacalifornica* Ayres, 1859	Pacific Angel Shark	X									
		Torpediniformes													
			**Torpedinidae (torpedo electric rays)**												
28				*Tetronarcecalifornica* (Ayres, 1855)	Pacific Electric Ray	X									
		Rajiformes													
			**Rhinobatidae (guitarfishes)**												
29				*Pseudobatosproductus* (Ayres, 1854)	Shovelnose Guitarfish	X	X		X					M/P	
			**Rajidae (skates)**												
30				*Amblyrajabadia* (Garman, 1899)	Broad Skate	X	X	X					P/V		
31				*Beringrajabinoculata* (Girard, 1855)	Big Skate	X									
32				*Beringrajainornata* (Jordan & Gilbert, 1881)	California Skate	X									
33				*Beringrajarhina* (Jordan & Gilbert, 1880)	Longnose Skate	X	X		X						
34				*Beringrajastellulata* (Jordan & Gilbert, 1880)	Starry Skate	X	X		X						
			**Arhynchobatidae (softnose skates)**												
35				*Bathyrajaabyssicola* (Gilbert, 1896)	Deepsea Skate	X	X	X					P/V		
36				*Bathyrajaaleutica* (Gilbert, 1896)	Aleutian Skate	X									
37				*Bathyrajakincaidii* (Garman, 1908)	Sandpaper Skate	X									
38				*Bathyrajatrachura* (Gilbert, 1892)	Roughtail Skate	X									
			**Platyrhinidae (thornbacks)**												
39				*Platyrhinoidistriseriata* (Jordan & Gilbert, 1880)	Thornback	X	X							M/P	
		Myliobatiformes													
			**Urotrygonidae (round stingrays)**												
40				*Urobatishalleri* (Cooper, 1863)	Round Stingray	X	X							M/P	
			**Dasyatidae (whiptail stingrays)**												
41				*Pteroplatytrygonviolacea* (Bonaparte, 1832)	Pelagic Stingray		X						P		
			**Myliobatidae (eagle rays)**												
42				*Myliobatiscalifornica* Gill, 1865	Bat Ray	X	X							M/P	
	ACTINOPTERI														
		Acipenseriformes													
			**Acipenseridae (sturgeons)**												
43				*Acipensermedirostris* Ayres, 1854	Green Sturgeon	X									
44				*Acipensertransmontanus* Richardson, 1836	White Sturgeon	X									
		Albuliformes													
			**Albulidae (bonefishes)**												
45				*Albulagilberti* Pfeiler & van der Heiden, 2011	Cortez Bonefish	X									
		Notacanthiformes													
			**Halosauridae (halosaurs)**												
46				*Aldrov&ia*cf.oleosa Sulak 1977	halosaur		X	X					P/V		
			**Notacanthidae (deep-sea spiny eels)**												
47				*Notacanthuschemnitzii* Bloch, 1788	Snubnosed Spiny Eel	X	X								
		Anguilliformes													
			**Synaphobranchidae (cutthroat eels)**												
48				SYNAPHOBRANCHIDAE sp. 1 (unidentified specimen)	cutthroat eel		X	X					P/V		
			**Ophichthidae (snake eels)**												
49				*Ophichthustriserialis* (Kaup, 1856)	Pacific Snake Eel	X	X							M	
50				*Ophichthuszophochir* Jordan & Gilbert, 1882	Yellow Snake Eel	X	X							M	
			**Nemichthyidae (snipe eels)**												
51				*Avocettinainfans* (Günther, 1878)	Blackline Snipe Eel	X									
52				*Nemichthysscolopaceus* Richardson, 1848	Slender Snipe Eel	X									
			**Nettastomatidae (duckbill eels)**												
53				*Facciolellaequatorialis* (Gilbert, 1891)	Dogface Witch Eel			X			X				
54				*Veneficatentaculata* Garman, 1899	Longnose Witch Eel		X	X					P/V		
			**Serrivomeridae (sawtooth eels)**												
55				*Serrivomersector* Garman, 1899	Sawtooth Eel	X	X	X					P/V		
		Saccopharyngiformes													
			**Cyematidae (bobtail eels)**												
56				*Cyemaatrum* Günther, 1878	Bobtail Eel	X	X	X					P/V		
			**Saccopharyngidae (whiptail gulpers)**												
57				*Saccopharynxlavenbergi* Nielsen & Bertelsen, 1985	Whiptail Gulper			X							
			**Eurypharyngidae (gulpers)**												
58				*Eurypharynxpelecanoides* Vaillant, 1882	Umbrellamouth Gulper			X							
		Clupeiformes													
			**Engraulidae (anchovies)**												
59				*Engraulismordax* Girard, 1854	Northern Anchovy	X	X						M	M/P	
			**Clupeidae (herrings)**												
60				*Alosasapidissima* (Wilson, 1811)	American Shad	X								M	X
61				*Clupeapallasii* Valenciennes, 1847	Pacific Herring	X	X							M/P	
62				*Dorosomapetenense* (Günther, 1867)	Threadfin Shad		X							P	X
63				*Etrumeusacuminatus* Gilbert, 1890	Round Herring	X	X								
64				*Sardinopssagax* (Jenyns, 1842)	Pacific Sardine	X							M	M	
		Argentiniformes													
			**Argentinidae (argentines)**												
65				*Argentina sialis* Gilbert, 1890	Pacific Argentine	X									
			**Microstomatidae (pencilsmelts)**												
66				*Microstoma* sp. (Pacific species)	pencilsmelt	X							M		
67				*Nansenia c&ida* Cohen, 1958	Bluethroat Argentine	X									
			**Bathylagidae (deepsea smelts)**												
68				*Bathylagoideswesethi* (Bolin, 1938)	Snubnose Blacksmelt	X	X		X				M		
69				*Bathylaguspacificus* Gilbert, 1890	Pacific Blacksmelt	X									
70				*Leuroglossusstilbius* Gilbert, 1890	California Smoothtongue	X							M		
71				*Lipolagusochotensis* (Schmidt, 1938)	Popeye Blacksmelt	X							M		
72				*Pseudobathylagusmilleri* (Jordan & Gilbert, 1898)	Robust Blacksmelt	X							M		
			**Opisthoproctidae (spookfishes)**												
73				*Bathylychnopsexilis* Cohen, 1958	Javelin Spookfish	X									
74				*Dolichopteryxlongipes* (Vaillant, 1888)	Brownsnout Spookfish	X									
75				*Macropinnamicrostoma* Chapman, 1939	Barreleye	X	X	X					M/P/V		
			**Alepocephalidae (slickheads)**												
76				*Alepocephalustenebrosus* Gilbert, 1892	California Slickhead	X									
77				*Talismaniabifurcata* (Parr, 1951)	Threadfin Slickhead	X									
			**Platytroctidae (tubeshoulders)**												
78				*Holtbyrnialatifrons* Sazonov, 1976	Streaklight Tubeshoulder	X									
79				*Mentoduseubranchus* (Matsui & Rosenblatt, 1987)	tubeshoulder	X							M		
80				*Sagamichthysabei* Parr, 1953	Shining Tubeshoulder	X							M		
		Osmeriformes													
			**Osmeridae (smelts)**												
81				*Allosmeruselongatus* (Ayres, 1854)	Whitebait Smelt	X									
82				*Hypomesuspretiosus* (Girard, 1854)	Surf Smelt	X								M	
83				*Spirinchusstarksi* (Fisk, 1913)	Night Smelt	X	X							M/P	
84				*Spirinchusthaleichthys* (Ayres, 1860)	Longfin Smelt	X									
85				*Thaleichthyspacificus* (Richardson, 1837)	Eulachon	X									
		Salmoniformes													
			**Salmonidae (trouts and salmons)**												
86				*Oncorhynchusgorbuscha* (Walbaum, 1792)	Pink Salmon		X			X					
87				*Oncorhynchusketa* (Walbaum, 1792)	Chum Salmon		X			X					
88				*Oncorhynchuskisutch* (Walbaum, 1792)	Coho Salmon	X	X								
89				*Oncorhynchusmykiss* (Walbaum, 1792)	Rainbow Trout (Steelhead)	X	X								
90				*Oncorhynchustshawytscha* (Walbaum, 1792)	Chinook Salmon	X	X								
		Stomiiformes													
			**Gonostomatidae (bristlemouths)**												
91				*Cyclothoneacclinidens* Garman, 1899	Benttooth Bristlemouth	X							M		
92				*Cyclothoneatraria* Gilbert, 1905	Black Bristlemouth	X							M		
93				*Cyclothonepallida* Brauer, 1902	Tan Bristlemouth	X									
94				*Cyclothonepseudopallida* Mukhacheva, 1964	Slender Bristlemouth	X							M		
95				*Cyclothonesignata* Garman, 1899	Showy Bristlemouth	X							M		
96				*Gonostomaatlanticum* Norman, 1930	Atlantic Fangjaw	X							M		
			**Sternoptychidae (marine hatchetfishes)**												
97				*Argyropelecusaffinis* Garman, 1899	Slender Hatchetfish	X							M		
98				*Argyropelecushemigymnus* Cocco, 1829	Spurred Hatchetfish	X							M		
99				*Argyropelecuslychnus* Garman, 1899	Tropical Hatchetfish	X									
100				*Argyropelecussladeni* Regan, 1908	Lowcrest Hatchetfish	X							M		
101				*Danaphosoculatus* (Garman, 1899)	Bottlelight	X							M		
102				*Sternoptyxdiaphana* Hermann, 1781	Longspine Hatchetfish	X									
103				*Sternoptyxobscura* Garman, 1899	Dusky Hatchetfish	X									
			**Phosichthyidae (lightfishes)**												
104				*Vinciguerrialucetia* (Garman, 1899)	Panama Lightfish	X									
			**Stomiidae (dragonfishes)**												
105				*Aristostomiasscintillans* (Gilbert, 1915)	Shiny Loosejaw	X	X		X						
106				*Bathophilusflemingi* Aron & McCrery, 1958	Highfin Dragonfish	X									
107				*Chauliodusmacouni* Bean, 1890	Pacific Viperfish	X							M		
108				*Idiacanthusantrostomus* Gilbert, 1890	Pacific Blackdragon	X							M		
109				*Stomiasatriventer* Garman, 1899	Blackbelly Dragonfish	X									
110				*Tactostomamacropus* Bolin, 1939	Longfin Dragonfish	X	X		X				M		
		Aulopiformes													
			**Synodontidae (lizardfishes)**												
111				*Synoduslucioceps* (Ayres, 1855)	California Lizardfish	X									
			**Notosudidae (waryfishes)**												
112			†	*Scopelosaurusadleri* (Fedorov, 1967)	Longfin Waryfish	X							M		
113				*Scopelosaurusharryi* (Mead, 1953)	Scaly Waryfish	X							M		
			**Scopelarchidae (pearleyes)**												
114				*Benthalbelladentata* (Chapman, 1939)	Northern Pearleye	X									
			**Alepisauridae (lancetfishes)**												
115				*Alepisaurusferox* Lowe, 1833	Longnose Lancetfish	X	X								
			**Anotopteridae (daggertooths)**												
116				*Anotopterusnikparini* Kukuev, 1998	North Pacific Daggertooth	X									
			**Paralepididae (barracudinas)**												
117				*Arctozenusrisso* (Bonaparte, 1840)	White Barracudina	X									
118				*Lestidiopsringens* (Jordan & Gilbert, 1880)	Slender Barracudina	X									
119				*Lestidiopssphyraenopsis* Hubbs, 1916	Smalleye Barracudina	X									
120				*Magnisudisatlantica* (Krøyer, 1868)	Duckbill Barracudina	X									
			**Bathysauridae (deepsea lizardfishes)**												
121				*Bathysaurusmollis* Günther, 1878	Highfin Lizardfish		X	X					P/V		
		Myctophiformes													
			**Myctophidae (lanternfishes)**												
122				*Ceratoscopelustownsendi* (Eigenmann & Eigenmann, 1889)	Dogtooth Lampfish	X							M		
123				*Diaphustheta* Eigenmann & Eigenmann, 1890	California Headlightfish	X							M		
124				*Diogenichthysatlanticus* (Tåning, 1928)	Longfin Lanternfish	X							M		
125				*Lampanyctussteinbecki* Bolin, 1939	Longfin Lampfish	X							M		
126			†	*Lampanyctustenuiformis* (Brauer, 1906)	lanternfish	X									
127				*Nannobrachiumregale* (Gilbert, 1892)	Pinpoint Lampfish	X							M		
128				*Nannobrachiumritteri* (Gilbert, 1915)	Broadfin Lampfish	X	X		X				M		
129				*Parviluxingens* Hubbs & Wisner, 1964	Giant Lampfish	X									
130				*Protomyctophumcrockeri* (Bolin, 1939)	California Flashlightfish	X							M		
131				*Protomyctophumthompsoni* (Chapman, 1944)	Northern Flashlightfish	X									
132				*Stenobrachiusleucopsarus* (Eigenmann & Eigenmann, 1890)	Northern Lampfish	X							M		
133				*Symbolophoruscaliforniensis* (Eigenmann & Eigenmann, 1889)	California Lanternfish	X							M		
134				*Taaningichthyspaurolychnus* Davy, 1972	Dimlight Lampfish	X							M		
135				*Tarletonbeaniacrenularis* (Jordan & Gilbert, 1880)	Blue Lanternfish	X							M		
136				*Triphoturusmexicanus* (Gilbert, 1890)	Mexican Lampfish	X							M		
		Lampridiformes													
			**Lampridae (opahs)**												
137				*Lamprisincognitus* (Underkoffler, Luers, Hyde, & Craig, 2018)	Smalleye Pacific Opah	X	X				X				
			**Trachipteridae (ribbonfishes)**												
138				*Desmodemalorum* Rosenblatt & Butler, 1977	Whiptail Ribbonfish	X		X					M		
139				*Trachipterusaltivelis* Kner, 1859	King-of-the-salmon	X	X								
		Gadiformes													
			**Macrouridae (grenadiers)**												
140				*Coelorinchusscaphopsis* (Gilbert, 1890)	Shoulderspot Grenadier	X									
141				*Coryphaenoidesacrolepis* (Bean, 1884)	Pacific Grenadier	X	X	X					P/V		
142				*Coryphaenoidesarmatus* (Hector, 1875)	Abyssal Grenadier		X	X					P/V		
143				*Coryphaenoidescinereus* (Gilbert, 1896)	Popeye Grenadier	X	X								
144				*Coryphaenoidesfilifer* (Gilbert, 1896)	Threadfin Grenadier		X	X					P/V		
145				*Coryphaenoidesleptolepis* Günther, 1877	Ghostly Grenadier		X	X					P/V		
146				*Coryphaenoidespectoralis* (Gilbert, 1892)	Giant Grenadier	X		X					V		
147				*Malacocephaluslaevis* (Lowe, 1843)	Softhead Grenadier	X	X								
148				*Nezumialiolepis* (Gilbert, 1890)	Smooth Grenadier	X	X								
149				*Nezumiastelgidolepis* (Gilbert, 1890)	California Grenadier	X	X								
			**Moridae (codlings)**												
150				*Antimoramicrolepis* Bean, 1890	Pacific Flatnose	X	X	X					P/V		
151				*Halargyreusjohnsonii* Günther, 1862	Slender Codling	X									
152				*Physiculusnematopus* Gilbert, 1890	Charcoal Codling	X									
153				*Physiculusrastrelliger* Gilbert, 1890	Hundred-fathom Codling	X									
			**Merlucciidae (merlucciid hakes)**												
154				*Merlucciusproductus* (Ayres, 1855)	Pacific Hake	X									
			**Gadidae (cods)**												
155				*Gaduschalcogrammus* Pallas, 1814	Walleye Pollock		X	X							
156				*Gadusmacrocephalus* Tilesius, 1810	Pacific Cod	X	X								
157				*Microgadusproximus* (Girard, 1854)	Pacific Tomcod	X									
		Ophidiiformes													
			**Ophidiidae (cusk-eels)**												
158				*Chilarataylori* (Girard, 1858)	Spotted Cusk-eel	X	X		X				M	P	
159				*Lamprogrammusniger* Alcock, 1891	Paperbone Cusk-eel	X									
160				*Luciobrotula* sp. A	cusk-eel		X	X					P/V		
161				*Spectrunculus gr&is* (Günther, 1877)	Giant Cusk-eel		X	X					P/V		
			**Bythitidae (viviparous brotulas)**												
162				*Brosmophycismarginata* (Ayres, 1854)	Red Brotula	X									
163				*Cataetyxrubrirostris* Gilbert, 1890	Rubynose Brotula	X	X								
		Batrachoidiformes													
			**Batrachoididae (toadfishes)**												
164				*Porichthysnotatus* Girard, 1854	Plainfin Midshipman	X	X							M/P	
		Lophiiformes													
			**Lophiidae (goosefishes)**												
165				*Lophiodesspilurus* (Garman, 1899)	Threadfin Goosefish	X	X				X				
			**Chaunacidae (gapers)**												
166				*Chaunacopscoloratus* (Garman, 1899)	gaper	X	X						M/P		
			**Melanocetidae (blackdevils)**												
167				*Melanocetusjohnsonii* Günther, 1864	Blackdevil	X		X							
			**Himantolophidae (footballfishes)**												
168				*Himantolophusnigricornis* Bertelsen & Krefft, 1988	footballfish	X									
169				*Himantolophussagamius* (Tanaka, 1918)	Pacific Footballfish	X	X								
			**Oneirodidae (dreamers)**												
170				*Chaenophrynelongiceps* Regan, 1925	dreamer	X									
171				*Chaenophrynemelanorhabdus* Regan & Trewavas, 1932	Smooth Dreamer	X									
			**Ceratiidae (seadevils)**												
172				*Cryptopsarascouesii* Gill, 1883	Triplewart Seadevil	X	X				X				
		Mugiliformes													
			**Mugilidae (mullets)**												
173				*Mugilcephalus* Linnaeus, 1758	Striped Mullet	X		X			X				
		Atheriniformes													
			**Atherinopsidae (New World silversides)**												
174				*Atherinopsaffinis* (Ayres, 1860)	Topsmelt	X	X							M/P	
175				*Atherinopsiscaliforniensis* Girard, 1854	Jacksmelt	X	X							M/P	
176				*Leuresthestenuis* (Ayres, 1860)	California Grunion	X	X				X				
		Beloniformes													
			**Exocoetidae (flyingfishes)**												
177				*Cheilopogonpinnatibarbatus* (Bennett, 1831)	Smallhead Flyingfish		X	X			X				
			**Belonidae (needlefishes)**												
178				*Strongyluraexilis* (Girard, 1854)	California Needlefish	X	X				X				
			**Scomberesocidae (sauries)**												
179				*Cololabissaira* (Brevoort, 1856)	Pacific Saury	X	X						V		
		Stephanoberyciformes													
			**Melamphaidae (bigscales)**												
180				*Melamphaeslugubris* Gilbert, 1890	Highsnout Bigscale	X							M		
181				*Poromitracrassiceps* (Günther, 1878)	Crested Bigscale	X							M		
182				*Scopelogadusbispinosus* (Gilbert, 1915)	Twospine Bigscale	X							M		
		Beryciformes													
			**Anoplogastridae (fangtooths)**												
183				*Anoplogastercornuta* (Valenciennes, 1833)	Fangtooth	X									
		Zeiformes													
			**Oreosomatidae (oreos)**												
184				*Allocyttusfolletti* Myers, 1960	Oxeye Oreo	X	X								
			**Zeidae (dories)**												
185				*Zenopsisnebulosa* (Temminck & Schlegel, 1845)	Mirror Dory	X									
		Gasterosteiformes													
			**Aulorhynchidae (tubesnouts)**												
186				*Aulorhynchusflavidus* Gill, 1861	Tubesnout	X									
			**Gasterosteidae (sticklebacks)**												
187				*Gasterosteusaculeatus* Linnaeus, 1758	Threespine Stickleback	X	X							M/P	
			**Syngnathidae (pipefishes)**												
188				*Cosmocampusarctus* (Jenkins & Evermann, 1889)	Snubnose Pipefish	X								M	
189				*Syngnathuscaliforniensis* Storer, 1845	Kelp Pipefish	X								M	
190				*Syngnathusexilis* (Osburn & Nichols, 1916)	Barcheek Pipefish	X								M	
191				*Syngnathusleptorhynchus* Girard, 1854	Bay Pipefish	X	X							M/P	
		Scorpaeniformes													
			**Scorpaenidae (scorpionfishes)**												
192				*Scorpaenaguttata* Girard, 1854	California Scorpionfish	X	X	X	X						
193				*Sebastesatrovirens* (Jordan & Gilbert, 1880)	Kelp Rockfish	X								P	
194				*Sebastesauriculatus* Girard, 1854	Brown Rockfish	X	X							M/P	
195				*Sebastesaurora* (Gilbert, 1890)	Aurora Rockfish	X									
196				*Sebastesbabcocki* (Thompson, 1915)	Redbanded Rockfish	X									
197				*Sebastesborealis* Barsukov, 1970	Shortraker Rockfish	X									
198				*Sebastesbrevispinis* (Bean, 1884)	Silvergray Rockfish	X									
199				*Sebastescarnatus* (Jordan & Gilbert, 1880)	Gopher Rockfish	X	X		X						
200				*Sebastescaurinus* Richardson, 1844	Copper Rockfish	X	X							M/P	
201				*Sebasteschlorostictus* (Jordan & Gilbert, 1880)	Greenspotted Rockfish	X	X		X						
202				*Sebasteschrysomelas* (Jordan & Gilbert, 1881)	Black-and-yellow Rockfish	X	X		X						
203				*Sebastesconstellatus* (Jordan & Gilbert, 1880)	Starry Rockfish	X									
204				*Sebastescrameri* (Jordan, 1897)	Darkblotched Rockfish	X									
205				*Sebastesdallii* (Eigenmann & Beeson, 1894)	Calico Rockfish	X	X		X					P	
206				*Sebastesdiaconus* Frable, Wagman, Frierson, Aguilar, & Sidlauskas, 2015	Deacon Rockfish	X	X								
207				*Sebastesdiploproa* (Gilbert, 1890)	Splitnose Rockfish	X									
208				*Sebasteselongatus* Ayres, 1859	Greenstriped Rockfish	X									
209				*Sebastesemphaeus* (Starks, 1911)	Puget Sound Rockfish	X									
210				*Sebastesensifer* Chen, 1971	Swordspine Rockfish	X									
211				*Sebastesentomelas* (Jordan & Gilbert, 1880)	Widow Rockfish	X	X		X						
212				*Sebasteseos* (Eigenmann & Eigenmann, 1890)	Pink Rockfish	X									
213				*Sebastesflavidus* (Ayres, 1862)	Yellowtail Rockfish	X									
214				*Sebastesgilli* (Eigenmann, 1891)	Bronzespotted Rockfish		X								
215				*Sebastesgoodei* (Eigenmann & Eigenmann, 1890)	Chilipepper	X									
216				*Sebasteshelvomaculatus* Ayres, 1859	Rosethorn Rockfish	X									
217				*Sebasteshopkinsi* (Cramer, 1895)	Squarespot Rockfish	X	X		X						
218				*Sebastesjordani* (Gilbert, 1896)	Shortbelly Rockfish	X									
219				*Sebasteslevis* (Eigenmann & Eigenmann, 1889)	Cowcod	X							M		
220				*Sebastesmacdonaldi* (Eigenmann & Beeson, 1893)	Mexican Rockfish	X	X				X				
221				*Sebastesmaliger* (Jordan & Gilbert, 1880)	Quillback Rockfish	X									
222				*Sebastesmelanops* Girard, 1856	Black Rockfish	X	X							P	
223				*Sebastesmelanostictus* (Matsubara, 1934)	Blackspotted Rockfish		X								
224				*Sebastesmelanostomus* (Eigenmann & Eigenmann, 1890)	Blackgill Rockfish	X									
225				*Sebastesminiatus* (Jordan & Gilbert, 1880)	Vermilion Rockfish	X	X		X						
226				*Sebastesmystinus* (Jordan & Gilbert, 1881)	Blue Rockfish	X	X							P	
227				*Sebastesnebulosus* Ayres, 1854	China Rockfish	X									
228				*Sebastesnigrocinctus* Ayres, 1859	Tiger Rockfish	X									
229				*Sebastesovalis* (Ayres, 1862)	Speckled Rockfish	X									
230				*Sebastespaucispinis* Ayres, 1854	Bocaccio	X	X						M	M/P	
231				*Sebastesphillipsi* (Fitch, 1964)	Chameleon Rockfish		X								
232				*Sebastespinniger* (Gill, 1864)	Canary Rockfish	X									
233				*Sebastesproriger* (Jordan & Gilbert, 1880)	Redstripe Rockfish	X	X		X						
234				*Sebastesrastrelliger* (Jordan & Gilbert, 1880)	Grass Rockfish	X	X							M/P	
235				*Sebastesrosaceus* Girard, 1854	Rosy Rockfish	X									
236				*Sebastesrosenblatti* Chen, 1971	Greenblotched Rockfish	X									
237				*Sebastesruberrimus* (Cramer, 1895)	Yelloweye Rockfish	X									
238				*Sebastesrubrivinctus* (Jordan & Gilbert, 1880)	Flag Rockfish	X	X		X						
239				*Sebastesrufus* (Eigenmann & Eigenmann, 1890)	Bank Rockfish	X									
240				*Sebastessaxicola* (Gilbert, 1890)	Stripetail Rockfish	X									
241				*Sebastessemicinctus* (Gilbert, 1897)	Halfbanded Rockfish	X									
242				*Sebastesserranoides* (Eigenmann & Eigenmann, 1890)	Olive Rockfish	X								M	
243				*Sebastesserriceps* (Jordan & Gilbert, 1880)	Treefish	X									
244				*Sebastessimulator* Chen, 1971	Pinkrose Rockfish	X									
245				*Sebastesumbrosus* (Jordan & Gilbert, 1882)	Honeycomb Rockfish	X									
246				*Sebasteswilsoni* (Gilbert, 1915)	Pygmy Rockfish	X	X		X						
247				*Sebasteszacentrus* (Gilbert, 1890)	Sharpchin Rockfish	X									
248				*Sebastolobusalascanus* Bean, 1890	Shortspine Thornyhead	X	X	X					P/V		
249				*Sebastolobusaltivelis* Gilbert, 1896	Longspine Thornyhead	X							M		
			**Triglidae (searobins)**												
250				*Prionotusstephanophrys* Lockington, 1881	Lumptail Searobin	X	X				X				
			**Anoplopomatidae (sablefishes)**												
251				*Anoplopomafimbria* (Pallas, 1814)	Sablefish	X	X								
252				*Erilepiszonifer* (Lockington, 1880)	Skilfish	X	X		X						
			**Hexagrammidae (greenlings)**												
253				*Hexagrammosdecagrammus* (Pallas, 1810)	Kelp Greenling	X								M	
254				*Hexagrammoslagocephalus* (Pallas, 1810)	Rock Greenling	X									
255				*Ophiodonelongatus* Girard, 1854	Lingcod	X	X							P	
256				*Oxylebiuspictus* Gill, 1862	Painted Greenling	X									
257				*Pleurogrammusmonopterygius* (Pallas, 1810)	Atka Mackerel	X	X								
258				*Zaniolepisfrenata* Eigenmann & Eigenmann, 1889	Shortspine Combfish	X									
259				*Zaniolepislatipinnis* Girard, 1858	Longspine Combfish	X									
			**Rhamphocottidae (grunt sculpins)**												
260				*Rhamphocottusrichardsonii* Günther, 1874	Grunt Sculpin	X									
			**Cottidae (sculpins)**												
261				*Artediuscorallinus* (Hubbs, 1926)	Coralline Sculpin	X									
262				*Artediusfenestralis* Jordan & Gilbert, 1883	Padded Sculpin	X									
263				*Artediusharringtoni* (Starks, 1896)	Scalyhead Sculpin	X								M	
264				*Artediuslateralis* (Girard, 1854)	Smoothhead Sculpin	X	X		X						
265				*Artediusnotospilotus* Girard, 1856	Bonyhead Sculpin	X								M	
266				*Ascelichthysrhodorus* Jordan & Gilbert, 1880	Rosylip Sculpin	X									
267				*Chitonotuspugetensis* (Steindachner, 1876)	Roughback Sculpin	X								M	
268				*Clinocottusacuticeps* (Gilbert, 1896)	Sharpnose Sculpin	X									
269				*Clinocottusanalis* (Girard, 1858)	Woolly Sculpin	X	X		X						
270				*Clinocottusembryum* (Jordan & Starks, 1895)	Calico Sculpin	X									
271				*Clinocottusglobiceps* (Girard, 1858)	Mosshead Sculpin	X									
272				*Clinocottusrecalvus* (Greeley, 1899)	Bald Sculpin	X	X		X						
273				*Enophrysbison* (Girard, 1854)	Buffalo Sculpin	X								M	
274				*Enophrystaurina* Gilbert, 1914	Bull Sculpin	X	X		X						
275				*Hemilepidotushemilepidotus* (Tilesius, 1811)	Red Irish Lord	X									
276				*Hemilepidotusspinosus* Ayres, 1854	Brown Irish Lord	X									
277				*Icelinusburchami* Evermann & Goldsborough, 1907	Dusky Sculpin	X									
278				*Icelinuscavifrons* Gilbert, 1890	Pit-head Sculpin	X									
279				*Icelinusfilamentosus* Gilbert, 1890	Threadfin Sculpin	X									
280				*Icelinusfimbriatus* Gilbert, 1890	Fringed Sculpin	X									
281				*Icelinusoculatus* Gilbert, 1890	Frogmouth Sculpin	X									
282				*Icelinusquadriseriatus* Lockington, 1880	Yellowchin Sculpin	X									
283				*Icelinustenuis* Gilbert, 1890	Spotfiin Sculpin	X									
284				*Jordaniazonope* Starks, 1895	Longfin Sculpin	X									
285				*Leptocottusarmatus* Girard, 1854	Pacific Staghorn Sculpin	X	X							M/P	
286				*Oligocottusmaculosus* Girard, 1856	Tidepool Sculpin	X									
287				*Oligocottusrimensis* (Greeley, 1899)	Saddleback Sculpin	X	X		X						
288				*Oligocottusrubellio* (Greeley, 1899)	Rosy Sculpin	X	X		X						
289				*Oligocottussnyderi* Greeley, 1898	Fluffy Sculpin	X	X		X						
290				*Orthonopiastriacis* Starks & Mann, 1911	Snubnose Sculpin	X	X								
291				*Radulinusasprellus* Gilbert, 1890	Slim Sculpin	X									
292				*Radulinusboleoides* Gilbert, 1898	Darter Sculpin	X									
293				*Ruscariuscreaseri* (Hubbs, 1926)	Roughcheek Sculpin	X									
294				*Scorpaenichthysmarmoratus* (Ayres, 1854)	Cabezon	X	X							M/P	
295				*Synchirusgilli* Bean, 1890	Manacled Sculpin	X									
296				*Zesticelusprofundorum* (Gilbert, 1896)	Flabby Sculpin	X	X								
			**Hemitripteridae (searavens)**												
297				*Blepsiascirrhosus* (Pallas, 1814)	Silverspotted Sculpin	X									
298				*Nautichthysoculofasciatus* (Girard, 1858)	Sailfin Sculpin	X									
			**Agonidae (poachers)**												
299				*Agonomalusmozinoi* Wilimovsky & Wilson, 1979	Kelp Poacher	X	X								
300				*Agonopsissterletus* (Gilbert, 1898)	Southern Spearnose Poacher	X									
301				*Agonopsisvulsa* (Jordan & Gilbert, 1880)	Northern Spearnose Poacher	X									
302				*Bathyagonuspentacanthus* (Gilbert, 1890)	Bigeye Poacher	X									
303				*Bothragonusswanii* (Steindachner, 1876)	Rockhead	X									
304				*Chesnoniaverrucosa* (Lockington, 1880)	Warty Poacher	X									
305				*Odontopyxistrispinosa* Lockington, 1880	Pygmy Poacher	X									
306				*Stellerinaxyosterna* (Jordan & Gilbert, 1880)	Pricklebreast Poacher	X	X		X						
307				*Xeneretmuslatifrons* (Gilbert, 1890)	Blacktip Poacher	X									
308				*Xeneretmusleiops* Gilbert, 1915	Smootheye Poacher	X									
309				*Xeneretmustriacanthus* (Gilbert, 1890)	Bluespotted Poacher	X									
			**Psychrolutidae (fathead sculpins)**												
310				*Psychrolutesphrictus* Stein & Bond, 1878	Blob Sculpin	X	X	X					P/V		
			**Liparidae (snailfishes)**												
311				*Bathyphasmaovigerum* Gilbert, 1896	Abyssal Snailfish		X	X							
312				*Careproctusfilamentosus* Stein, 1978	snailfish		X	X							
313				*Careproctusgilberti* Burke, 1912	Smalldisk Snailfish	X	X								
314				*Careproctuskamikawai* Orr, 2012	Arbiter Snailfish	X	X	X	X				V		
315				*Careproctuslongifilis* Garman, 1892	Threadfin Snailfish	X	X								
316				*Careproctusmelanurus* Gilbert, 1892	Blacktail Snailfish	X	X	X							
317				*Elassodiscuscaudatus* (Gilbert, 1915)	Humpback Snailfish	X	X		X						
318				*Liparisflorae* (Jordan & Starks, 1895)	Tidepool Snailfish	X									
319				*Liparisfucensis* Gilbert, 1896	Slipskin Snailfish	X									
320				*Liparismucosus* Ayres, 1855	Slimy Snailfish	X									
321				*Liparispulchellus* Ayres, 1855	Showy Snailfish	X									
322				*Lipariscusnanus* Gilbert, 1915	Pygmy Snailfish	X	X		X						
323				*Nectoliparispelagicus* Gilbert & Burke, 1912	Tadpole Snailfish	X	X								
324				*Osteodiscuscascadiae* Stein, 1978	Bonydisk Snailfish	X	X								
325				*Paraliparisalbescens* Gilbert, 1915	Phantom Snailfish	X	X		X						
326				*Paralipariscephalus* Gilbert, 1892	Swellhead Snailfish	X									
327				*Paraliparisdactylosus* Gilbert, 1896	Polydactyl Snailfish	X	X		X						
328				*Paraliparisdeani* Burke, 1912	Prickly Snailfish	X	X								
329				*Paraliparismento* Gilbert, 1892	Bulldog Snailfish	X	X								
330				*Paraliparispectoralis* Stein, 1978	Pectoral Snailfish	X									
331				*Paraliparisrosaceus* Gilbert, 1890	Rosy Snailfish	X									
332				*Paraliparisulochir* Gilbert, 1896	Broadfin Snailfish	X									
333				*Rhinoliparisattenuatus* Burke, 1912	Slim Snailfish	X	X								
334				*Rhinoliparisbarbulifer* Gilbert, 1896	Longnose Snailfish	X	X								
		Perciformes													
			**Moronidae (temperate basses)**												
335				*Moronesaxatilis* (Walbaum, 1791)	Striped Bass	X	X							M/P	X
			**Polyprionidae (wreckfishes)**												
336				*Stereolepisgigas* Ayres, 1859	Giant Sea Bass	X	X				X				
			**Epinephelidae (groupers)**												
337				*Hyporthodusniphobles* (Gilberts & Starks, 1897)	Star-studded Grouper	X					X				
338				*Mycteropercaxenarcha* Jordan, 1888	Broomtail Grouper	X									
			**Serranidae (sea basses and groupers)**												
339				*Paralabraxclathratus* (Girard, 1854)	Kelp Bass	X	X				X				
340			†	*Paralabraxmaculatofasciatus* (Steindachner, 1868)	Spotted Sand Bass		X			X	X				
341				*Paralabraxnebulifer* (Girard, 1854)	Barred Sand Bass	X	X		X						
			**Priacanthidae (bigeyes)**												
342				*Pristigenysserrula* (Gilbert, 1891)	Popeye Catalufa	X	X				X				
			**Malacanthidae (tilefishes)**												
343				*Caulolatilusprinceps* (Jenyns, 1840)	Ocean Whitefish		X				X				
			**Coryphaenidae (dolphinfishes)**												
344				*Coryphaenahippurus* Linnaeus, 1758	Dolphinfish		X				X				
			**Echeneidae (remoras)**												
345				*Remoraaustralis* (Bennett, 1840)	Whalesucker	X									
346				*Remoraremora* (Linnaeus, 1758)	Remora	X									
			**Carangidae (jacks)**												
347				*Caranxcaballus* Günther, 1868	Green Jack	X	X				X				
348				*Decapterusmuroadsi* (Temminck & Schlegel, 1844)	Amberstripe Scad	X	X								
349				*Naucratesductor* (Linnaeus, 1758)	Pilotfish	X									
350				*Serioladorsalis* (Gill, 1863)	Yellowtail Jack	X									
351				*Trachurussymmetricus* (Ayres, 1855)	Jack Mackerel	X	X						P	M	
			**Bramidae (pomfrets)**												
352				*Bramajaponica* Hilgendorf, 1878	Pacific Pomfret	X									
353				*Pteraclisaesticola* (Jordan & Snyder, 1901)	Pacific Fanfish	X									
			**Caristiidae (manefishes)**												
354				*Caristiusmacropus* (Bellotti, 1903)	Bigmouth Manefish	X									
			**Haemulidae (grunts)**												
355				*Haemuloncaliforniensis* (Steindachner, 1876)	Salema	X	X			X	X				
			**Polynemidae (threadfins)**												
356				*Polydactylusapproximans* (Lay & Bennett, 1839)	Blue Bobo	X	X				X				
			**Sciaenidae (drums and croakers)**												
357				*Atractoscionnobilis* (Ayres, 1860)	White Seabass		X				X				
358				*Genyonemuslineatus* (Ayres, 1855)	White Croaker	X	X							M	
359			†	*Menticirrhusundulatus* (Girard, 1854)	California Corbina		X			X	X				
360				*Seriphuspolitus* Ayres, 1860	Queenfish	X	X							M/P	
			**Kyphosidae (sea chubs)**												
361				*Girellanigricans* (Ayres, 1860)	Opaleye	X	X								
362				*Hermosillaazurea* Jenkins & Evermann, 1889	Zebraperch		X	X							
363				*Kyphosusvaigiensis* (Quoy & Gaimard 1825)	Blue-bronze Chub	X					X				
364				*Medialunacaliforniensis* (Steindachner, 1876)	Halfmoon	X	X				X				
			**Pentacerotidae (armorheads)**												
365				*Pseudopentaceroswheeleri* (Hardy, 1983)	North Pacific Armorhead	X									
			**Oplegnathidae (knifejaws)**												
366				*Oplegnathusfasciatus* (Temminck & Schlegel, 1844)	Barred Knifejaw		X	X							X
			**Embiotocidae (surfperches)**												
367				*Amphistichusargenteus* Agassiz, 1854	Barred Surfperch	X									
368				*Amphistichuskoelzi* (Hubbs, 1933)	Calico Surfperch	X									
369				*Amphistichusrhodoterus* (Agassiz, 1854)	Redtail Surfperch	X									
370				*Brachyistiusfrenatus* Gill, 1862	Kelp Perch	X									
371				*Cymatogasteraggregata* Gibbons, 1854	Shiner Perch	X	X							M/P	
372				*Embiotocacaryi* Agassiz, 1853	Rainbow Seaperch	X								M	
373				*Embiotocajacksoni* Agassiz, 1853	Black Perch	X	X							M/P	
374				*Embiotocalateralis* Agassiz, 1854	Striped Seaperch	X									
375				*Hyperprosoponargenteum* Gibbons, 1854	Walleye Surfperch	X	X							M/P	
376				*Hyperprosoponellipticum* (Gibbons, 1854)	Silver Surfperch	X								M	
377				*Hypocritichthysanalis* (Agassiz, 1861)	Spotfin Surfperch	X									
378				*Micrometrusaurora* (Jordan & Gilbert, 1880)	Reef Perch	X	X		X						
379				*Micrometrusminimus* (Gibbons, 1854)	Dwarf Perch	X	X							M/P	
380				*Phanerodonatripes* (Jordan & Gilbert, 1880)	Sharpnose Seaperch	X	X		X						
381				*Phanerodonfurcatus* Girard, 1854	White Seaperch	X	X							M/P	
382				*Phanerodonvacca* (Girard, 1855)	Pile Perch	X	X							M/P	
383				*Rhacochilustoxotes* Agassiz, 1854	Rubberlip Seaperch	X	X							M/P	
384				*Zalembiusrosaceus* (Jordan & Gilbert, 1880)	Pink Seaperch	X									
			**Pomacentridae (damselfishes)**												
385				*Chromispunctipinnis* (Cooper, 1863)	Blacksmith	X	X				X				
386				*Hypsypopsrubicundus* (Girard, 1854)	Garibaldi	X	X	X	X	X	X				
			**Labridae (wrasses)**												
387				*Oxyjuliscalifornica* Günther, 1861	Señorita	X	X		X					M	
388				*Semicossyphuspulcher* (Ayres, 1854)	California Sheephead	X	X				X				
			**Bathymasteridae (ronquils)**												
389				*Rathbunellaalleni* Gilbert, 1904	Stripefin Ronquil	X	X		X						
390				*Ronquilusjordani* (Gilbert, 1889)	Northern Ronquil	X									
			**Zoarcidae (eelpouts)**												
391				*Bothrocarabrunneum* (Bean, 1890)	Twoline Eelpout	X	X	X					P/V		
392				*Bothrocaramolle* Bean, 1890	Soft Eelpout	X									
393				*Eucryphycuscalifornicus* (Starks & Mann, 1911)	Persimmon Eelpout	X	X								
394				*Lycenchelyscallista* &erson, 1995	eelpout	X	X		X						
395				*Lycenchelyscamchatica* (Gilbert & Burke, 1912)	Kamchatka Eelpout	X	X								
396				*Lycenchelyscrotalinus* (Gilbert, 1890)	Snakehead Eelpout	X									
397				*Lycenchelysjordani* (Evermann & Goldsborough, 1907)	Shortjaw Eelpout	X									
398				*Lycenchelysmicropora* &riashev, 1955	Manytoothed Eelpout	X									
399				*Lycenchelysmonstrosa* &erson, 1982	eelpout	X									
400				*Lycodapusdermatinus* Gilbert, 1896	Looseskin Eelpout	X									
401				*Lycodapusfierasfer* Gilbert, 1890	Blackmouth Eelpout	X	X	X					P/V		
402				*Lycodapus m&ibularis* Gilbert, 1915	Pallid Eelpout	X	X	X	X				P/V		
403				*Lycodapuspachysoma* Peden & &erson, 1978	Stout Eelpout	X							M		
404				*Lycodapuspsarostomatus* Peden & &erson, 1981	Specklemouth Eelpout	X									
405				*Lycodescortezianus* (Gilbert, 1890)	Bigfin Eelpout	X									
406				*Lycodesdiapterus* Gilbert, 1892	Black Eelpout	X									
407				*Lycodespacificus* (Collett, 1879)	Blackbelly Eelpout	X									
408				*Lyconemabarbatum* Gilbert, 1896	Bearded Eelpout	X	X		X						
409				*Melanostigmapammelas* Gilbert, 1896	Midwater Eelpout	X	X		X						
410				*Pachycarabulbiceps* (Garman, 1899)	Snubnose Eelpout		X	X					P/V		
411				*Pachycarakarenae* &erson, 2012	eelpout	X	X		X						
			**Stichaeidae (pricklebacks)**												
412				*Anoplarchuspurpurescens* Gill, 1861	High Cockscomb	X									
413				*Cebidichthysviolaceus* (Girard, 1854)	Monkeyface Prickleback	X								M	
414				*Chirolophisnugator* (Jordan & Williams, 1895)	Mosshead Warbonnet	X									
415				*Ernogrammuswalkeri* Follett & Powell, 1988	Masked Prickleback	X	X		X						
416				*Esselenichthyscarli* (Follett & &erson, 1990)	Threeline Prickleback	X	X								
417				*Kasatkiaseigeli* Posner & Lavenberg, 1999	Sixspot Prickleback	X									
418				*Phytichthyschirus* (Jordan & Gilbert, 1880)	Ribbon Prickleback	X	X		X						
419				*Plagiogrammushopkinsii* Bean, 1894	Crisscross Prickleback	X	X		X						
420				*Plectobranchusevides* Gilbert, 1890	Bluebarred Prickleback	X	X								
421				*Xiphisteratropurpureus* (Kittlitz, 1858)	Black Prickleback	X									
422				*Xiphistermucosus* (Girard, 1858)	Rock Prickleback	X									
			**Pholidae (gunnels)**												
423				*Apodichthysflavidus* Girard, 1854	Penpoint Gunnel	X								M	
424				*Apodichthysfucorum* Jordan & Gilbert, 1880	Rockweed Gunnel	X	X		X						
425				*Pholisclemensi* Rosenblatt, 1964	Longfin Gunnel		X	X							
426				*Pholisornata* (Girard, 1854)	Saddleback Gunnel	X									
427				*Pholisschultzi* Schultz, 1931	Red Gunnel	X									
428				*Ulvicolasanctaerosae* Gilbert & Starks, 1897	Kelp Gunnel	X									
			**Anarhichadidae (wolffishes)**												
429				*Anarrhichthysocellatus* Ayres, 1855	Wolf-eel	X									
			**Zaproridae (prowfishes)**												
430				*Zaprorasilenus* Jordan, 1896	Prowfish	X	X								
			**Scytalinidae (graveldivers)**												
431				*Scytalinacerdale* Jordan & Gilbert, 1880	Graveldiver	X									
			**Chiasmodontidae (swallowers)**												
432				*Chiasmodonniger* Johnson 1864	Black Swallower	X	X	X							
433				*Kaliindica* Lloyd, 1909	Shortnose Swallower	X									
			**Trichodontidae (sandfishes)**												
434				*Trichodontrichodon* (Tilesius, 1813)	Pacific Sandfish		X		X	X					
			**Ammodytidae (sand lances)**												
435				*Ammodyteshexapterus* Pallas, 1814	Pacific Sand Lance	X								M	
			**Uranoscopidae (stargazers)**												
436				*Kathetostomaaverruncus* Jordan & Bollman, 1890	Smooth Stargazer	X					X				
			**Blenniidae (combtooth blennies)**												
437				*Hypsoblenniusgentilis* (Girard, 1854)	Bay Blenny	X	X		X					M	
438				*Hypsoblenniusgilberti* (Jordan, 1882)	Rockpool Blenny	X									
439				*Hypsoblenniusjenkinsi* (Jordan & Evermann, 1896)	Mussel Blenny	X	X				X				
			**Clinidae (kelp blennies)**												
440				*Gibbonsiaelegans* (Cooper, 1864)	Spotted Kelpfish	X					X				
441				*Gibbonsiametzi* Hubbs, 1927	Striped Kelpfish	X	X		X					M	
442				*Gibbonsiamontereyensis* Hubbs, 1927	Crevice Kelpfish	X	X		X						
443				*Heterostichusrostratus* Girard, 1854	Giant Kelpfish	X								M	
			**Chaenopsidae (tube blennies)**												
444				*Neoclinusblanchardi* Girard, 1858	Sarcastic Fringehead	X								M	
445				*Neoclinusstephensae* Hubbs, 1953	Yellowfin Fringehead	X		X			X				
446				*Neoclinusuninotatus* Hubbs, 1953	Onespot Fringehead	X	X							P	
			**Icosteidae (ragfishes)**												
447				*Icosteusaenigmaticus* Lockington, 1880	Ragfish	X	X								
			**Gobiesocidae (clingfishes)**												
448				*Gobiesox mae&ricus* (Girard, 1858)	Northern Clingfish	X									
449				*Rimicolamuscarum* (Meek & Pierson, 1895)	Kelp Clingfish	X	X		X						
			**Gobiidae (gobies)**												
450				*Acanthogobiusflavimanus* (Temminck & Schlegel, 1845)	Yellowfin Goby	X	X							M/P	X
451				*Clevel&ia ios* (Jordan & Gilbert, 1882)	Arrow Goby	X	X							M/P	
452				*Gillichthysmirabilis* Cooper, 1864	Longjaw Mudsucker	X	X							M/P	
453				*Lepidogobiuslepidus* (Girard, 1858)	Bay Goby	X	X							M/P	
454				*Lethopsconnectens* Hubbs, 1926	Halfblind Goby	X	X		X						
455				*Lythrypnusdalli* (Gilbert, 1890)	Bluebanded Goby	X					X				
456				*Lythrypnuszebra* (Gilbert, 1890)	Zebra Goby	X									
457				*Rhinogobiopsnicholsii* (Bean, 1882)	Blackeye Goby	X								M	
458				*Typhlogobiuscaliforniensis* Steindachner, 1879	Blind Goby	X									
			**Luvaridae (louvars)**												
459				*Luvarusimperialis* Rafinesque, 1810	Louvar	X									
			**Sphyraenidae (barracudas)**												
460				*Sphyraenaargentea* Girard, 1854	Pacific Barracuda	X	X				X				
			**Gempylidae (snake mackerels)**												
461				*Ruvettuspretiosus* Cocco, 1833	Oilfish	X					X				
			**Trichiuridae (cutlassfishes)**												
462				*Aphanopusintermedius* Parin, 1983	Intermediate Scabbardfish	X									
463				*Benthodesmuspacificus* Parin & Becker, 1970	North Pacific Frostfish	X									
464				*Lepidopusfitchi* Rosenblatt & Wilson, 1987	Pacific Scabbardfish	X									
			**Scombridae (mackerels)**												
465				*Auxisrochei* (Risso, 1810)	Bullet Mackerel	X					X				
466				*Katsuwonuspelamis* (Linnaeus, 1758)	Skipjack Tuna		X				X		P		
467				*Sardachiliensis* (Cuvier, 1832)	Pacific Bonito		X				X				
468				*Scomberjaponicus* Houttuyn, 1782	Pacific Chub Mackerel	X	X				X				
469				*Scomberomorusconcolor* (Lockington, 1879)	Gulf Sierra	X	X		X		X				
470				*Thunnusalalunga* (Bonnaterre, 1788)	Albacore	X	X				X		P		
471				*Thunnusalbacares* (Bonnaterre, 1788)	Yellowfin Tuna		X				X				
472				*Thunnusobesus* (Lowe, 1839)	Bigeye Tuna		X				X				
473				*Thunnusorientalis* (Temminck & Schlegel, 1844)	Pacific Bluefin Tuna	X	X				X				
			**Xiphiidae (swordfishes)**												
474				*Xiphiasgladius* Linnaeus, 1758	Swordfish		X				X		P		
			**Istiophoridae (billfishes)**												
475				*Kajikiaaudax* (Philippi, 1887)	Striped Marlin		X				X				
			**Centrolophidae (medusafishes)**												
476				*Icichthyslockingtoni* Jordan & Gilbert, 1880	Medusafish	X							M		
			**Tetragonuridae (squaretails)**												
477				*Tetragonuruscuvieri* Risso, 1810	Smalleye Squaretail	X	X				X		P		
			**Stromateidae (butterfishes)**												
478				*Peprilussimillimus* (Ayres, 1860)	Pacific Pompano	X								M	
		Pleuronectiformes													
			**Paralichthyidae (sand flounders)**												
479				*Citharichthyssordidus* (Girard, 1854)	Pacific Sanddab	X							M	M	
480				*Citharichthysstigmaeus* Jordan & Gilbert, 1882	Speckled Sanddab	X	X						M	M/P	
481				*Citharichthysxanthostigma* Gilbert, 1890	Longfin Sanddab	X	X								
482				*Hippoglossinastomata* Eigenmann & Eigenmann, 1890	Bigmouth Sole	X	X								
483				*Paralichthyscalifornicus* (Ayres, 1859)	California Halibut	X	X							M/P	
484				*Xystreurysliolepis* Jordan & Gilbert, 1880	Fantail Sole	X	X								
			**Pleuronectidae (righteye flounders)**												
485				*Atheresthesevermanni* Jordan & Starks, 1904	Kamchatka Flounder	X	X								
486				*Atheresthesstomias* (Jordan & Gilbert, 1880)	Arrowtooth Flounder	X									
487				*Clidodermaasperrimum* (Temminck & Schlegel, 1846)	Roughscale Sole	X									
488				*Embassichthysbathybius* (Gilbert, 1890)	Deepsea Sole	X									
489				*Eopsettajordani* (Lockington, 1879)	Petrale Sole	X									
490				*Glyptocephaluszachirus* Lockington, 1879	Rex Sole	X							M		
491				*Hippoglossoideselassodon* Jordan & Gilbert, 1880	Flathead Sole	X	X								
492				*Hippoglossusstenolepis* Schmidt, 1904	Pacific Halibut		X								
493				*Isopsettaisolepis* (Lockington, 1880)	Butter Sole	X									
494				*Lepidopsettabilineata* (Ayres, 1855)	Rock Sole	X	X								
495				*Lyopsettaexilis* (Jordan & Gilbert, 1880)	Slender Sole	X	X								
496				*Microstomuspacificus* (Lockington, 1879)	Dover Sole	X							M		
497				*Parophrysvetulus* Girard, 1854	English Sole	X	X							M/P	
498				*Platichthysstellatus* (Pallas, 1787)	Starry Flounder	X	X							M/P	
499				*Pleuronichthyscoenosus* Girard, 1854	C-O Sole	X									
500				*Pleuronichthysdecurrens* Jordan & Gilbert, 1881	Curlfin Turbot	X								P	
501				*Pleuronichthysguttulatus* Girard, 1856	Diamond Turbot	X	X							M/P	
502				*Pleuronichthysverticalis* Jordan & Gilbert, 1880	Hornyhead Turbot	X									
503				*Psettichthysmelanostictus* Girard, 1854	Sand Sole	X	X								
			**Cynoglossidae (tonguefishes)**												
504				*Symphurusatricaudus* (Jordan & Gilbert, 1880)	California Tonguefish	X	X							M/P	
		Tetraodontiformes													
			**Balistidae (triggerfishes)**												
505				*Balistespolylepis* Steindachner, 1876	Finescale Triggerfish	X	X				X				
			**Diodontidae (porcupinefishes)**												
506				*Diodonholocanthus* Linnaeus, 1758	Balloonfish	X					X				
			**Molidae (molas)**												
507				*Molamola* (Linnaeus, 1758)	Ocean Sunfish	X	X								
	SUBTOTAL					465	230	44	57	7	49	1	83	79	5

† Listed with reservation.

Seven species are considered historic: Pink Salmon (*Oncorhynchusgorbuscha*); Chum Salmon (*Oncorhynchusketa*); Spotted Sand Bass (*Paralabraxmaculatofasciatus*); Salema (*Haemuloncaliforniensis*); California Corbina (*Menticirrhusundulatus*); Garibaldi (*Hypsypopsrubicundus*); and Pacific Sandfish (*Trichodontrichodon*). These species have not been observed within MBNMS for many years, and/or have rarely been observed. Their recent rarity can be attributed to local extinction (i.e., salmon), or otherwise a northern or southern species that has rarely been observed in sanctuary waters. Although rarely observed or not observed for some time, they could potentially occur again and remain on the checklist.

At least 50 species are known to occur during unusual oceanographic events (e.g., warm or cold phases of El Niño/Southern Oscillation). The 49 species occurring during warm-water events do not necessarily occur solely during warm-water events (e.g., El Niño), but they are more likely to be observed at these times (see Table 3). The occurrence of the Whale Shark (*Rhincodontypus*) was reported during several cold-water events (La Niña, Ebert et al. 2004). These observations seem counterintuitive, as *R.typus* typically occupies tropical waters (Miller and Lea 1972, Ebert 2003, Love et al. 2005, Castro 2011). We include the cold-water categorization here, as reported by Ebert et al. (2004).

Species occurring within two specific habitats of the sanctuary, Elkhorn Slough and Davidson Seamount, are noted due to the uniqueness of each habitat compared to the rest of MBNMS. Of the 507 species in MBNMS, 79 species are found in the Elkhorn Slough area, and 83 species in the Davidson Seamount area. The majority of these species are not restricted to these habitats within MBNMS, although Elkhorn Slough may be the exception. This is not surprising due to the mixture of brackish and saltwater habitats, and the life history requirements of some species. Elkhorn Slough also provides habitat for marine species from nearshore waters to feed, mate, and spawn (Yoklavich et al. 2002). Five species are less likely to occur in marine environments: Threadfin Shad (*Dorosomapetenense*; introduced species); Threespine Stickleback (*Gasterosteusaculeatus*); Yellowfin Goby (*Acanthogobiusflavimanus*; introduced species); Arrow Goby (*Clevelandiaios*); and Longjaw Mudsucker (*Gillichthysmirabilis*). The Davidson Seamount Management Zone occurs far offshore and the fish fauna there is influenced by oceanic water masses to the north and west, such as the Subarctic-Transitional and Central Pacific water masses. The 83 species occurring at Davidson Seamount are not restricted to the seamount and occur elsewhere in deep-sea or oceanic habitats.

Five species are non-native, that were either purposefully or inadvertently introduced to California: American Shad (*Alosasapidissima*); Threadfin Shad (*Dorosomapetenense*); Striped Bass (*Moronesaxatilis*); Barred Knifejaw (*Oplegnathusfasciatus*); and Yellowfin Goby (*Acanthogobiusflavimanus*). The Barred Knifejaw is the most recent introduction to MBNMS (2014, one confirmed living specimen), and it remains to be seen if it will establish itself as a viable resident.

Four species are listed with reservation: waryfish (*Scopelosaurusadleri*); lanternfish (*Lampanyctustenuiformis*); Spotted Sand Bass (*Paralabraxmaculatofasciatus*); and California Corbina (*Menticirrhusundulatus*). Reservation with the first two of these species is due to lack of confidence with identification; the latter two are categorized as historic and/or occurring during warm-water events, and evidence is scant (see checklist for explanation). We include these species on the checklist with the basis as cited.

Eighteen species are considered to be extralimital, are not considered part of the primary checklist, and are listed separately. The geographic ranges for these species encompass MBNMS boundaries. They are likely to occur within MBNMS; however, no verifiable records occur from within MBNMS. These species should be anticipated during future MBNMS surveys and deposited and catalogued at a natural history museum.

In recent years erroneous geographical distributions were introduced in the literature for two species: Basketweave Cusk-eel (*Ophidionscrippsae*) and Spotted Turbot (*Pleuronichthysritteri*). Both errors were based on misidentifications in the field that were then perpetuated in the literature. Once these sorts of mistakes occur and are repeated, these myths become difficult to eradicate. Our purpose in emphasizing these examples is to shed light on such problems and resolve these misrepresentations. First, the northern limit for *Ophidionscrippsae* has been reported as “Central California” (Love et al. 2005; Kells et al. 2016) based on NOAA trawl survey field identifications off the Santa Cruz County coast (lat. 37°38’N, 209 m; Lauth 2001). This represented the first record north of Point Arguello (34°34’38”N, 120°39’2”W; 11.75 nautical miles NW of the Point Conception faunal boundary) and from a depth greatly exceeding its known bathymetric limit. The accessioned specimens (UW 47375) were examined by RN Lea and re-determined as Spotted Cusk-eel (*Chilarataylori*), with both location and depth well within the limits for *Chilarataylori*. The known northern limit for *Ophidionscrippsae* is off the vicinity of Point Arguello (SIO 48-304, 3 specimens) and the maximum confirmed depth is 135 m (off central Baja California). Similarly, the northern limit for *Pleuronichthysritteri* was reported off Northern California (Love et al. 2005; Kells et al. 2016) based on field-identified specimens (lat. 37°55’N; Weinberg et al. 2002). The accessioned material (UW 47438) was examined by RN Lea and JW Orr (NOAA, NMFS) and re-determined as Hornyhead Turbot (*Pleuronichthysverticalis*), a species relatively common within MBNMS. *Pleuronichthysritteri* is primarily a southern California–Baja California shallow water species (Fitch 1963) with a known northern limit of Morro Bay (LACM 33703-1). We bring these emendations to the attention of the reader to avoid further confusion and note there are no confirmed records of either *Ophidionscrippsae* or *Pleuronichthysritteri* from MBNMS.

This is the first annotated checklist of fishes that occur within Monterey Bay National Marine Sanctuary (see Table 3). All fishes listed here also occur outside of MBNMS boundaries, and full species ranges can be found from other sources (e.g., Miller and Lea 1972, Eschmeyer et al. 1983, Love et al. 2002, Ebert 2003, Love et al. 2005, and Kells et al. 2016).

Species checklists or inventories can be used by various user groups for different reasons. A simple checklist can teach and inform users of what to expect within a geographic area, including educators, students, and those interested in regional natural history. This annotated checklist includes name-bearing type specimen information for students and ichthyologists interested in species discoveries (and original descriptions) that occurred within today’s MBNMS boundaries. Those interested in zoogeography of fishes may be interested in historic and recent occurrence information for fishes within MBNMS. In addition, warm-water and cold-water event information is included to help explain potentially temporary occurrence patterns, or sporadic events. An inventory of known sanctuary resources is a basic requirement of the National Marine Sanctuaries Act (NMSA) of 1972, as amended (16 U.S.C. § 1431 et seq.). Species inventories provide evidence of occurrence and estimates of species richness. Establishing an inventory is a crucial first step to further identify those species that are endemic, threatened, introduced (provided herein), or socio-economically important, information which may be useful for sanctuary managers. It is our hope that this checklist will be useful for many readers and serve as a model for species inventories for the National Marine Sanctuary System.

### Annotated checklist

#### Order Myxiniformes

##### Family MYXINIDAE – hagfishes

***Eptatretusdeani*** (Evermann & Goldsborough, 1907). **Black Hagfish**. Museum specimens: CAS-ICH 39879 (west of Yankee Point, WC Ruark); CAS-ICH 42563 (off Cypress Point, RN Lea); MLMLF0003 (Monterey Bay, R Parrish).

***Eptatretusstoutii*** (Lockington, 1878). **Pacific Hagfish**. Museum specimens: MLMLF0007 (1.5 miles south of Kirby Park at Elkhorn Slough, LT Ackerman); CAS-SU 58418 (Monterey Bay, JC Briggs); CAS-SU 66927 (Monterey Bay, CL Hubbs).

#### Order Petromyzontiformes

##### Family PETROMYZONIDAE – lampreys

***Entosphenustridentatus*** (Gairdner, 1836). **Pacific Lamprey**. Museum specimens: MLMLF0010 (Monterey Bay, “on throat of *Onchorhynchustshawytscha*”, LT Ackerman); MLMLF0011 (Moss Landing beach; Ackerman, Lewis, Lindquist); CAS-SU 11299 (Pacific Grove, identifier unknown).

#### Order Chimaeriformes

##### Family CHIMAERIDAE – shortnose chimaeras

***Hydrolaguscolliei*** (Lay & Bennett, 1839). **Spotted Ratfish**. Described from Monterey. Museum specimens: Type material (original material not preserved); CAS-ICH 51176 (west of Davenport, L Podshadley); CAS-SU 10829 (Monterey, RL Bolin); CAS-SU 62378 (off Point Sur, RL Bolin). Publication: Lay and Bennett (1839) (original description).

**Hydrolaguscf.trolli** Didier and Séret, 2002. **Pointy-nosed Blue Chimaera**. Publications: Lundsten et al. (2009) and Reichert et al. (2016) (Davidson Seamount). Visual records: MBARI T1102-04 (Davidson Seamount, DA Didier and D Ebert; Jacobson Stout et al. 2017); MBARI T1075-02 (Monterey Canyon DA Didier and D Ebert; Jacobson Stout et al. 2017). Naming convention follows Reichert et al. (2016); specimens cannot yet be confirmed as *Hydrolagustrolli* until morphometric data and/or DNA samples from preserved specimens have been collected and analyzed. Common name follows Didier and Séret (2002).

#### Order Heterodontiformes

##### Family HETERODONTIDAE – horn sharks

***Heterodontusfrancisci*** (Girard, 1855). **Horn Shark**. Described from ‘Bay of Monterey, Cal.’. Museum specimens: Type material (original material not located); USNM 112106 (Monterey, DS Jordan). Publication: Girard (1855) (original description).

#### Order Orectolobiformes

##### Family RHINCODONTIDAE – whale sharks

***Rhincodontypus*** Smith, 1828. **Whale Shark**. Publication: Ebert et al. (2004) (based on visual records, Monterey Bay during 1928-1930, 1929, 1944, 1947, and off Big Sur during 1989). Ebert et al. (2004) state all reported local sightings (where season and year recorded) were during La Niña years. Categorized as occurring during cold-water events (i.e., La Niña) based on Ebert et al (2004).

#### Order Lamniformes

##### Family ALOPIIDAE – thresher sharks

***Alopiasvulpinus*** (Bonnaterre, 1788). **Common Thresher Shark**. Museum specimens: CAS-ICH 65976 (off Point San Pedro at San Mateo County, WI Follett); MLMLF0024 (off Manressa State Beach, ME Anderson).

##### Family CETORHINIDAE – basking sharks

***Cetorhinusmaximus*** (Gunnerus, 1765). **Basking Shark**. Museum specimens: CAS-ICH 2224 (15 miles off Santa Cruz, idenfier unknown); CAS-ICH 26246 (off Salinas River mouth, identifier unknown). Publications: Jordan and Gilbert (1880a) (Monterey during 1880); Jordan (1887a) (Santa Cruz County, Monterey Bay during 1880); Jordan (1887b) (Monterey Bay fishery during 1880); Scofield (1917) (Monterey Bay); Starks (1921) (Monterey); Thompson (1921a) (Monterey); Phillips (1948) (Monterey Bay fishery during 1946-1947, with reference to harpooning for sport in Monterey Bay during 1924); Follett (1966) (off Salinas River mouth, CAS-ICH 26246).

##### Family LAMNIDAE – mackerel sharks

***Carcharodoncarcharias*** (Linnaeus, 1758). **White Shark**. Museum specimens: CAS-ICH 26245 (off Salinas River mouth, WI Follett); CAS-ICH 26308 (Pebble Beach, Monterey County; WI Follett and LJ Dempster); CAS-ICH 26678 (off La Selva Beach, WI Follett). Publication: Jordan and Gilbert (1880a) (“Point Carmelo, near Monterey”).

***Isurusoxyrinchus*** Rafinesque, 1810. **Shortfin Mako**. Museum specimens: CAS-ICH 53202 (west of Princeton Harbor during 1983, El Niño, identifier unknown); CAS-ICH 55473 (off Santa Cruz, identifier unknown). Categorized as occurring during warm-water events (e.g., El Niño).

***Lamnaditropis*** Hubbs and Follett, 1947. **Salmon Shark**. Museum specimens: CAS-ICH 26710 (Monterey Bay, WI Follett and LJ Dempster); CAS-SU 12656 (Monterey Bay, LJV Compagno). Publication: Carlisle et al. (2015) (numerous strandings along US west coast; in MBNMS from Montara State Beach to San Simeon area).

#### Order Carcharhiniformes

##### Family SCYLIORHINIDAE – cat sharks

Based on molecular data and morphology, Iglesias et al. (2005) proposed resurrection of family Pentanchidae, and assigned the content of Pentanchinae (i.e., including the genera *Apristurus* and *Parmaturus*) as in Compagno (1988). This proposed change is not yet widely accepted; therefore, we chose to keep deepwater catsharks in the family Scyliorhinidae.

***Apristurusbrunneus*** (Gilbert, 1892). **Brown Cat Shark**. Museum specimens: CAS-ICH 37479 (Monterey Bay, LJV Compagno); CAS-ICH 37512 (west of Yankee Point, WN Eschmeyer); MLMLF0028 (off Point Sur, ME Anderson).

***Apristuruskampae*** Taylor, 1972. **Longnose Cat Shark**. Museum specimens: CAS-ICH 58482 (off Point Sur, RN Lea); CAS-ICH 58487 (west of Point Año Nuevo, RN Lea); MLMLF0030 (off Cypress Point, ME Anderson). Publication: Anderson et al. (1979) (off Cypress Point, MLMLF0030).

***Cephaloscylliumventriosum*** (Garman, 1880). **Swell Shark**. Museum specimens: CAS-ICH 18050 (Monterey, LJV Compagno); CAS-ICH 52983 (Santa Cruz, M Moriguchi).

***Parmaturusxaniurus*** (Gilbert, 1892). **Filetail Cat Shark**. Museum specimens: CAS-ICH 37499 (west of Point Año Nuevo, T Iwamoto); CAS-ICH 37513 (west of Yankee Point, WN Eschmeyer); MLMLF0036 (Monterey Bay, L Talent).

##### Family TRIAKIDAE – hound sharks

***Galeorhinusgaleus*** (Linnaeus, 1758). **Tope**. Museum specimens: CAS-ICH 55475 (Monterey Bay area, WI Follett); MLMLF0040 (Monterey Bay, ME Anderson); CAS-SU 4148 (Monterey, LJV Compagno).

***Musteluscalifornicus*** Gill, 1864. **Gray Smoothhound**. Museum specimens: CAS-ICH 13158 (Monterey Bay, JVL Compagno); CAS-ICH 65045 (Elkhorn Slough, WI Follett); MLMLF0043 (Kirby Park at Elkhorn Slough, LT Ackerman). Publications: Herald (1953) (Elkhorn Slough); Yoklavich et al. (1991) (Kirby Park and Hudson’s Landing at Elkhorn Slough).

***Mustelushenlei*** (Gill, 1863). **Brown Smoothhound**. Museum specimens: CAS-ICH 40629 (Elkhorn Slough, LJV Compagno); MLMLF0046 (Monterey Bay, JP Harville); MLMLF047 (Monterey Bay, L Compagno).

***Triakissemifasciata*** Girard, 1855. **Leopard Shark**. Museum specimens: CAS-ICH 13162 (Monterey Bay, WM Morton); CAS-ICH 75994 (Kirby Park at Elkhorn Slough, DT Anderson); MLMLF0050 (1.5 mile below Kirby Park at Elkhorn Slough, LT Ackerman). Publications: Herald (1953) (Elkhorn Slough); Herald and Dempster (1952) (Elkhorn Slough); Yoklavich et al. (1991) (Hwy 1 Bridge, Dairy, Kirby Park, and Hudson’s Landing at Elkhorn Slough).

##### Family CARCHARHINIDAE – requiem sharks

***Prionaceglauca*** (Linnaeus, 1758). **Blue Shark**. Museum specimens: CAS-ICH 65086 (Moss Beach, WI Follett); SIO 62-413 (SW of Pigeon Point, RL McNeely); CAS-SU 53183 (off Santa Cruz, WC Freihofer). Publication: Fitch (1949) (Davidson Seamount).

#### Order Hexanchiformes

##### Family HEXANCHIDAE – cow sharks

***Hexanchusgriseus*** (Bonnaterre, 1788). **Bluntnose Sixgill Shark**. Museum specimens: MLMLF0014 (off Davenport, LT Ackerman); CAS-SU 13291 (Monterey Bay, EC Starks); CAS-SU 49011 (Monterey Bay area, WI Follett).

***Notorynchuscepedianus*** (Péron, 1807). **Broadnose Sevengill Shark**. Museum specimen: CAS-SU 40909 (“Monterey Bay area of California?”, WI Follett). In addition, 15 collections from San Francisco Bay are accessioned at California Academy of Sciences; which presumably traversed MBNMS waters.

#### Order Squaliformes

##### Family ECHINORHINIDAE – bramble sharks

***Echinorhinuscookei*** Pietschmann, 1928. **Prickly Shark**. Museum specimens: MLMLF0068 (Monterey Canyon, D Lewis); UF 41736 (off Moss Landing, D Ebert).

##### Family SQUALIDAE – dogfish sharks

***Squalussuckleyi*** (Girard, 1855). **Pacific Spiny Dogfish**. Museum specimens: MLMLF0064 (off Santa Cruz, GV Morejohn); MLMLF0065 (Monterey Bay, ME Anderson); CAS-SU 58376 (Monterey Bay, JC Briggs).

##### Family SOMNIOSIDAE – sleeper sharks

***Somniosuspacificus*** Bigelow and Schroeder, 1944. **Pacific Sleeper Shark**. Museum specimens: CAS-ICH 53524 (off Pescadero Point, T Iwamoto); MLMLF0061 (Monterey Bay, ME Anderson); MLMLF0062 (Monterey Bay, GV Morejohn). Publication: Anderson et al. (1979) (Monterey Bay, MLMLF0062).

#### Order Squatiniformes

##### Family SQUATINIDAE – angel sharks

***Squatinacalifornica*** Ayres, 1859. **Pacific Angel Shark**. Museum specimens: CAS-ICH 65090 (Monterey Bay, identifier unknown); MLMLF0069 (Monterey Bay, D Lewis).

#### Order Torpediniformes

##### Family TORPEDINIDAE – torpedo electric rays

***Tetronarcecalifornica*** (Ayres, 1855). **Pacific Electric Ray**. Museum specimens: CAS-ICH 37472 (Monterey Bay, T Iwamoto); CAS-SU 21416 (Monterey Bay, LJV Compagno); CAS-SU 58396 (Monterey Bay, JC Briggs). Previously recognized as *Torpedocalifornica*. We follow Carvalho et al. (2016), Weigmann (2016), and current usage in *Catalog of Fishes* (Eschmeyer et al. 2017); with placement in *Tetronarce*.

#### Order Rajiformes

##### Family RHINOBATIDAE – guitarfishes

***Pseudobatosproductus*** (Ayres, 1854). **Shovelnose Guitarfish**. Described from Monterey. Museum specimens: Syntype (USNM 1009); CAS-ICH 65978 (Moss Landing at Elkhorn Slough, WI Follett); MLMLF0079 (Kirby Park at Elkhorn Slough, LT Ackerman). Publications: Ayres (1854) (original description); Herald and Dempster (1952) (Elkhorn Slough); Herald (1953) (Elkhorn Slough). Previously recognized as *Rhinobatosproductus*. We follow Last et al. (2016a), Séret et al. (2016), and current usage in *Catalog of Fishes* (Eschmeyer et al. 2017); with placement in *Pseudobatos*.

###### Family RAJIDAE – skates

***Amblyrajabadia*** (Garman, 1899). **Broad Skate**. Museum specimen: CAS-ICH 58604 (Point Sur, M Stehmann and RN Lea). Publication: Burton and Lundsten (2008) (Davidson Seamount, based on imagery). Visual record: MBARI/NOAA D943-06 (Davidson Seamount, D Ebert and L Kuhnz; Jacobson Stout et al. 2017). Last et al. (2016c) suggest *A.badia* may be junior synonym of *A.hyperborea*. Proposed change is unresolved and not widely accepted; we do not accept revision at this time. Common name follows Ebert (2003).

***Beringrajabinoculata*** (Girard, 1855). **Big Skate**. Museum specimens: CAS-ICH 12875 (off Point Montara Light, EC Johnston); CAS-ICH 40328 (5.5. miles SSW of Point Piedras Blancas, WC Ruark); CAS-ICH 47407 (west of Pajaro River mouth, L Ayres); MLMLF0104 (Monterey Bay, E Osada).

***Beringrajainornata*** (Jordan & Gilbert, 1881). **California Skate**. Museum specimens: CAS-ICH 232351 (west of Santa Cruz, JD Hopkirk); CAS-SU 4032 (Monterey, DS Jordan); CAS-SU 23988 (Pacific Grove, EC Starks). Previously recognized as *Rajainornata*. We follow Last et al. (2016c), and current usage in *Catalog of Fishes* (Eschmeyer et al. 2017); with placement in *Beringraja*. *Raja* not found within eastern Pacific.

***Beringrajarhina*** (Jordan & Gilbert, 1880). **Longnose Skate**. Described from Monterey Bay and San Francisco Bay. Museum specimens: Syntypes (3 specimens, accession location unknown); CAS-ICH 39891 (Monterey, WC Ruark); CAS-SU 61651 (Monterey Bay, JC Briggs). Publication: Jordan and Gilbert (1880h) (original description). Previously recognized as *Rajarhina*. We follow Last et al. (2016c), and current usage in *Catalog of Fishes* (Eschmeyer et al. 2017); with placement in *Beringraja*. *Raja* not found within eastern Pacific.

***Beringrajastellulata*** (Jordan & Gilbert, 1880). **Starry Skate**. Described from Monterey Bay. Museum specimens: Paratypes [ANSP 414 (1), MNHN A-3295 (1), USNM 26975 (orig. 8, now 2), ZMB 11687 (1), ZMUB 877 (1)]; CAS-ICH 26919 (Pacific Grove, R Ishiyama); CAS-ICH 224344 (off Davenport, D Ebert and C Davis); CAS-SU 13288 (Santa Cruz, LJV Campangno). Publication: Jordan and Gilbert (1880d) (original description). Previously recognized as *Rajastellulata*. We follow Last et al. (2016c), and current usage in *Catalog of Fishes* (Eschmeyer et al. 2017); with placement in *Beringraja*. *Raja* not found within eastern Pacific.

###### Family ARHYNCHOBATIDAE – softnose skates

*Bathyraja* previously recognized in family Rajidae. We follow Last et al. (2016b), Last et al. (2016c), and current usage in *Catalog of Fishes* (Eschmeyer et al. 2017); with placement in family Arhynchobatidae.

***Bathyrajaabyssicola*** (Gilbert, 1896). **Deepsea Skate**. Museum specimens: CAS-ICH 38013 (west of Yankee Point, WN Eschmeyer); CAS-ICH 58481 (off Point Sur, RN Lea). Publication: Burton and Lundsten (2008) (Davidson Seamount, based on imagery). Visual records: MBARI/NOAA T427-07 and MBARI/NOAA T427-09 (Davidson Seamount, D Ebert and L Kuhnz).

***Bathyrajaaleutica*** (Gilbert, 1896). **Aleutian Skate**. Museum specimen: CAS-ICH 243646 (Año Nuevo Canyon, identifier unknown; RN Lea examined specimen, radiograph, and confirmed presence of scapular spines).

***Bathyrajakincaidii*** (Garman, 1908). **Sandpaper Skate**. Museum specimens: CAS-ICH 39852 (off Hurricane Point, RN Lea); CAS-ICH 58485 (west of Point Año Nuevo, RN Lea, previously *Bathyrajainterrupta*); CAS-SU 5434 (near La Cruz Canyon, ME Anderson, previously *B.interrupta*). Previously recognized as *B.interupta*. We follow Last et al. (2016b), with placement in *Bathyraja*. California records previously identified as *B.interrupta* now deemed to be *B.kincaidii*; *B.interrupta* not known south of Bering Sea.

***Bathyrajatrachura*** (Gilbert, 1892). **Roughtail Skate**. Museum specimens: CAS-ICH 47392 (Monterey Canyon, RN Lea); CAS-ICH 56106 (off Point Sur, ME Anderson); CAS-ICH 58486 (west of Point Año Nuevo, RN Lea). Common name follows Ebert (2003).

###### Family PLATYRHINIDAE – thornbacks

***Platyrhinoidistriseriata*** (Jordan & Gilbert, 1880). **Thornback**. Museum specimens: CAS-ICH 20586 and CAS-ICH 63204 (Elkhorn Slough, ES Herald); MLMLF0074 (Elkhorn Slough, A King). Publication: Herald (1953) (Elkhorn Slough). Ordinal classification unresolved at present; here, placed in Order Rajiformes.

#### Order Myliobatiformes

##### Family UROTRYGONIDAE – round stingrays

***Urobatishalleri*** (Cooper, 1863). **Round Stingray**. Museum specimens: CAS-ICH 20587 and CAS-ICH 31371 (Elkhorn Slough, ES Herald); MLMLF0116 (1.5 miles below Kirby Park at Elkhorn Slough, LT Ackerman). Publications: Herald (1953) (Elkhorn Slough); Yoklavich et al. (1991) (Dairy and Kirby Park at Elkhorn Slough).

##### Family DASYATIDAE – whiptail stingrays

***Pteroplatytrygonviolacea*** (Bonaparte, 1832). **Pelagic Stingray**. Publication: Mollet (2002) (bycatch in California drift gill net fishery for swordfish within Davidson Seamount Management Zone).

##### Family MYLIOBATIDAE – eagle rays

***Myliobatiscalifornica*** Gill, 1865. **Bat Ray**. Museum specimens: MLMLF0113 (Kirby Park at Elkhorn Slough, E Yarberry); CAS-SU 47326 (Elkhorn Slough, RL Bolin). Publications: Herald (1953) (Elkhorn Slough); Herald and Dempster (1952) (Elkhorn Slough); Yoklavich et al. (1991) (Hwy 1 Bridge, Dairy, Kirby Park, and Hudson’s Landing at Elkhorn Slough).

#### Order Acipenseriformes

##### Family ACIPENSERIDAE – sturgeons

***Acipensermedirostris*** Ayres, 1854. **Green Sturgeon**. Museum specimens: CAS-ICH 53083 (Santa Cruz Pier, D Rawls); CAS-SU 55089 (Moss Landing Beach, WC Freihofer and HH DeWitt). Anadromous.

***Acipensertransmontanus*** Richardson, 1836. **White Sturgeon**. Museum specimen: CAS-ICH 82295 (Pajaro River Lagoon, RN Lea). Although museum specimens were not collected within MBNMS, this is an anadromous species that enters the ocean (adjacent MBNMS) during its life cycle.

#### Order Albuliformes

##### Family ALBULIDAE – bonefishes

***Albulagilberti*** Pfeiler and van der Heiden, 2011. **Cortez Bonefish**. Museum specimens (under old name *Albulavulpes*): CAS-SU 13034 (Monterey Bay, identifier unknown); CAS-SU 35301 (Monterey during Mar 1941, El Niño, RL Bolin).

#### Order Notacanthiformes

##### Family HALOSAURIDAE – halosaurs

**Aldrovandiacf.oleosa** Sulak 1977. **halosaur**. Publications: Burton and Lundsten (2008) and Lundsten et al. (2009) (Davidson Seamount, based on imagery, as *Aldrovandia* sp.). Visual record: MBARI/NOAA T427-11 (Davidson Seamount, GM Cailliet and RH Rosenblatt; Jacobson Stout et al. 2017, as *Aldrovandia* sp.). Specimen observed at Davidson Seamount (video) confirmed to genus by GM Cailliet (MLML) and R Rosenblatt (SIO); unable to identify to species level. However, Kamikawa and Stevenson (2010) state *A.oleosa* is the only halosaur and known species of *Aldrovandia* from the eastern North Pacific. No official common name.

##### Family NOTACANTHIDAE – deep-sea spiny eels

***Notacanthuschemnitzii*** Bloch, 1788. **Snubnosed Spiny Eel**. Museum specimens: CAS-ICH 58441 (Monterey Canyon, RN Lea); SIO 85-52 (off Point Sur, W Wakefield); SIO 88-99 (south of Point Sur, identifier unknown, NMFS). Publication: Lea and Rosenblatt (1987) (Monterey Canyon, CAS-ICH 58441; off Point Sur, SIO 85-52).

#### Order Anguilliformes

##### Family SYNAPHOBRANCHIDAE – cutthroat eels

**SYNAPHOBRANCHIDAE sp. 1**. **cutthroat eel**. Publications: Burton and Lundsten (2008), and Lundsten et al. (2009) (Davidson Seamount, based on imagery). Visual record: MBARI/NOAA T945-04 (Davidson Seamount, EJ Burton, RN Lea, and L Lundsten; Jacobson Stout et al. 2017). Specimen observed at Davidson Seamount (video) was identified to the family level by EJ Burton (MBNMS), RN Lea (CAS), and L Lundsten (MBARI); Burton and Lundsten (2008). This is the only known record of Synaphobranchidae in MBNMS and may represent a new species (Lundsten et al. 2009). No official common name.

##### Family OPHICHTHIDAE – snake eels

***Ophichthustriserialis*** (Kaup, 1856). **Pacific Snake Eel**. Museum specimen: MLMLF0139 (Moss Landing Harbor during Nov 1967, R Parrish; re-examined by RN Lea, 2017; 1002 mm). Miller and Lea (1972) consider rare from California. This species has also been taken from San Francisco Bay (Hopkirk 1965) and WSW of the Klamath River (Quirollo and Dinnel 1975).

***Ophichthuszophochir*** Jordan and Gilbert, 1882. **Yellow Snake Eel.** Museum specimen: MLMLF0140 (powerplant intake screen at Moss Landing Harbor during Apr 1969, D Varoujean). This species is rather common in southern California waters (Hopkirk 1965). Other specimens were collected from San Francisco Bay in 1964 (CAS-ICH 23683, Hopkirk 1965) and 1999 (leptocephalus stage, Lea and Rosenblatt 2000). Specimens were most likely transported northward, through the MBNMS area, during a warm-water event (Lea and Rosenblatt 2000). This species has also been taken from Humboldt Bay during Jan 1971 (Quirollo and Dinnel 1975).

##### Family NEMICHTHYIDAE – snipe eels

***Avocettinainfans*** (Günther, 1878). **Blackline Snipe Eel.** Museum specimen: CAS-ICH 56233 (Point Sur, ME Anderson).

***Nemichthysscolopaceus*** Richardson, 1848. **Slender Snipe Eel**. Museum specimen: CAS-ICH 216851 (off Point Sur, RN Lea).

##### Family NETTASTOMATIDAE - duckbill eels

***Facciolellaequatorialis*** (Gilbert, 1891). **Dogface Witch Eel**. Visual records: CSUMB Dive 75-T1 (Carmel Canyon during Jun 2010, ~345 m, 2 specimens, reviewed by RN Lea; SIMoN (2018) (IfAME/MBNMS/MARE/TNC). Observation occurred during warm-water event (e.g., El Niño).

***Veneficatentaculata*** Garman, 1899. **Longnose Witch Eel**. Publication: Burton and Lundsten (2008) (Davidson Seamount, based on imagery). Visual record: MBARI/NOAA T427-11 (Davidson Seamount, GM Cailliet, RN Lea, and RH Rosenblatt).

##### Family SERRIVOMERIDAE – sawtooth eels

***Serrivomersector*** Garman, 1899. **Sawtooth Eel**. Museum specimen: SIO 67-112 (Monterey Canyon, CL Hubbs). Publication: Burton and Lundsten (2008) (Davidson Seamount, based on imagery). Visual record: MBARI/NOAA T944-10 (Davidson Seamount, Jacobson Stout et al. 2017).

#### Order Saccopharyngiformes

##### Family CYEMATIDAE – bobtail eels

***Cyemaatrum*** Günther, 1878. **Bobtail Eel**. Museum specimen: CAS-ICH 63700 (Monterey Canyon, R Bolin). Publication: Burton and Lundsten (2008) (Davidson Seamount, based on imagery). Visual record: MBARI/NOAA T425-11 (Davidson Seamount, EJ Burton and T Trejo).

##### Family SACCOPHARYNGIDAE – whiptail gulpers

***Saccopharynxlavenbergi*** Nielsen and Bertelsen, 1985. **Whiptail Gulper**. Visual records: MBARI T622-07 and MBARI V2869-05 (Monterey Bay, Jacobson Stout et al. 2017).

##### Family EURYPHARYNGIDAE – gulpers

***Eurypharynxpelecanoides*** Vaillant, 1882. **Umbrellamouth Gulper**.Visual record: MBARI D198-02 (Monterey Canyon, Jacobson Stout et al. 2017).

#### Order Clupeiformes

##### Family ENGRAULIDAE – anchovies

***Engraulismordax*** Girard, 1854. **Northern Anchovy**. Museum specimens: CAS-ICH 65975 (off Point San Pedro, WI Follett); CAS-ICH 211630 (Monterey Canyon, RL Bolin); CAS-ICH 232634 (Elkhorn Slough, DW Behrens); SWFSC uncatalogued (Davidson Seamount during May 2015, W Watson). Publication: Yoklavich et al. (1991) (Hwy 1 Bridge, Dairy, Kirby Park, and Hudson’s Landing at Elkhorn Slough).

##### Family CLUPEIDAE – herrings

***Alosasapidissima*** (Wilson, 1811). **American Shad**. Museum specimens: KU 23736 (west of Point Año Nuevo, M Domeier); MLMLF0146 (Elkhorn Slough near railroad bridge, LT Ackerman); SIO 05-88 (west of Lucia, D Kamikawa). Introduced from the Atlantic; anadromous (Miller and Lea 1972).

***Clupeapallasii*** Valenciennes, 1847. **Pacific Herring**. Museum specimens: CAS-ICH 25755 (Santa Cruz Municipal Wharf, WI Follett); CAS-SU 58410 (Elkhorn Slough, JC Briggs); CAS-SU 60786 (Monterey Bay, RL Bolin). Publication: Yoklavich et al. (1991) (Dairy, Kirby Park, and Hudson’s Landing at Elkhorn Slough).

***Dorosomapetenense*** (Günther, 1867). **Threadfin Shad**. Publication: Yoklavich et al. (1991) (Kirby Park and Hudson’s Landing at Elkhorn Slough). Findings presented by Yoklavich et al. (1991) are based on otter trawl studies by Barry (1983) and Cailliet et al. (1977). Native to southeastern U.S., and along eastern coast of Mexico and Central America; introduced into California freshwater lakes (Miller and Lea 1972, Dill and Cordone 1997). Live mainly in fresh water and become progressively less abundant as salinity increases; however, can survive and grow in seawater (Moyle 2002).

***Etrumeusacuminatus*** Gilbert, 1890. **Round Herring**. Museum specimens: CAS-ICH 59626 (off Monterey breakwater at Monterey Bay, RN Lea); CAS-SU 14165 (Monterey Bay, JE Randall). Publication: Phillips (1951b) (one mile offshore between Cape San Martin and Piedras Blancas). Previously recognized as *Etrumeusteres* (now known to be restricted to eastern North Atlantic). We follow naming convention of Randall and DiBattista (2012), and current usage in *Catalog of Fishes* (Eschmeyer et al. 2017).

***Sardinopssagax*** (Jenyns, 1842). **Pacific Sardine**. Museum specimens: CAS-ICH 54931 (Monterey Bay, RN Lea); CAS-SU 58378 (Elkhorn Slough, JC Briggs); SWFSC uncatalogued (Davidson Seamount during May 2015, W Watson).

#### Order Argentiniformes

##### Family ARGENTINIDAE – argentines

***Argentina sialis*** Gilbert, 1890. **Pacific Argentine**. Museum specimens: MLMLF0167 and MLMLF0168 (Monterey Bay, ME Anderson); CAS-SU 35311 (Monterey Bay, RL Bolin); CAS-SU 49737 (off Point Pinos, J Phillips).

##### Family MICROSTOMATIDAE – pencilsmelts

***Microstoma* sp.** (Pacific species). **pencilsmelt**. Museum specimens: CAS-ICH 239292 (Davidson Seamount, RN Lea and EJ Burton); SIO 67-102 (within Davidson Seamount Management Zone, CL Hubbs); CAS-SU 49800 (Monterey Canyon, TN Fast). Recognized by some researchers as *Microstomamicrostoma*. We follow Ahlstrom et al. (1984), and Moser and Butler (1996), where North Pacific is represented by a single undescribed species (*Microstoma* sp.). No official common name.

***Nanseniacandida*** Cohen, 1958. **Bluethroat Argentine**. Museum specimen: LACM-36264.001 (west of Monterey, identifier unknown).

##### Family BATHYLAGIDAE – deep-sea smelts

Four genera previously recognized in family Microstomatidae include: *Bathylagoides, Leuroglossus, Lipolagus*, and *Pseudobathylagus*. We follow Nelson et al. (2004), Nelson et al. (2016), and current usage in *Catalog of Fishes* (Eschmeyer et al. 2017), with placement in family Bathylagidae.

***Bathylagoideswesethi*** (Bolin, 1938). **Snubnose Blacksmelt**. Described from Monterey Bay. Museum specimens: Holotype (CAS-SU 32971); Paratypes (CAS-SU 32972, CAS-SU 32973); CAS-ICH 239291 (Davidson Seamount, RN Lea and EJ Burton); SIO 67-102 (within Davidson Seamount Management Zone, CL Hubbs). Publications: Bolin (1938) (original description).

***Bathylaguspacificus*** Gilbert, 1890. **Pacific Blacksmelt**. Museum specimens: CAS-ICH 54824 (Monterey Canyon, ME Anderson); CAS-ICH 55061 (off Point Sur, ME Anderson); CAS-ICH 98332 (Monterey Canyon, RL Bolin).

***Leuroglossusstilbius*** Gilbert, 1890. **California Smoothtongue**. Museum specimens: CAS-ICH 239320 and CAS-ICH 239348 (Davidson Seamount, RN Lea and EJ Burton); SIO 67-102 (within Davidson Seamount Management Zone, CL Hubbs).

***Lipolagusochotensis*** (Schmidt, 1938). **Popeye Blacksmelt**. Museum specimens: CAS-ICH 239374 (off Monterey, RN Lea and EJ Burton); SIO 11-250 (west of Lucia, P Davison); SIO 85-57 (Monterey Canyon, W Wakefield); SWFSC uncatalogued (Davidson Seamount during May 2015, W Watson).

***Pseudobathylagusmilleri*** (Jordan & Gilbert, 1898). **Robust Blacksmelt**. Museum specimens: SIO 67-102 (within Davidson Seamount Management Zone, CL Hubbs); SWFSC uncatalogued (Davidson Seamount during May 2015, W Watson).

##### Family OPISTHOPROCTIDAE – spookfishes

***Bathylychnopsexilis*** Cohen, 1958. **Javelin Spookfish**. Museum specimen: CAS-ICH 74351 (Monterey Bay, F Tsuji).

***Dolichopteryxlongipes*** (Vaillant, 1888). **Brownsnout Spookfish**. Museum specimen: UW 113807 (NE of Cabrillo Canyon, D Kamikawa).

***Macropinnamicrostoma*** Chapman, 1939. **Barreleye**. Museum specimens: CAS-SU 48781 (Monterey Canyon, TN Fast); MLMLF0200 (Monterey Canyon, S Zeiner); SWFSC uncatalogued (Davidson Seamount during May 2015, W Watson). Publications: Bradbury and Cohen (1958) (Monterey Bay); Robison and Reisenbichler (2008) (Monterey Bay and Davidson Seamount). Visual records: MBARI V2571-05 (Monterey Bay, Robison and Reisenbichler 2008, Jacobson Stout et al. 2017); MBARI T0987-02 (Davidson Seamount, Robison and Reisenbichler 2008, Jacobson Stout et al. 2017).

##### Family ALEPOCEPHALIDAE – slickheads

***Alepocephalustenebrosus*** Gilbert, 1892. **California Slickhead**. Museum specimens: CAS-ICH 42921 (Partington Canyon, B Antrim); MLMLF0202 (Monterey Bay, RH Parrish); MLMLF0204 (Ascension Canyon, ME Anderson); CAS-SU 65825 (Monterey Canyon, RL Bolin and TN Fast).

***Talismaniabifurcata*** (Parr, 1951). **Threadfin Slickhead**. Museum specimens: CAS-ICH 36776 (Monterey Bay, ME Anderson); CAS-ICH 59661 (Monterey Bay, RN Lea).

##### Family PLATYTROCTIDAE – tubeshoulders

***Holtbyrnialatifrons*** Sazonov, 1976. **Streaklight Tubeshoulder**. Museum specimens: MLMLF0211 (Monterey Bay, ME Anderson); SIO 67-112 (Monterey Canyon, CL Hubbs).

***Mentoduseubranchus*** (Matsui & Rosenblatt, 1987). **tubeshoulder**. Museum specimen: CAS-ICH 239349 (Davidson Seamount, RN Lea and EJ Burton). This is the first known record of occurrence within MBNMS (unpublished data, Burton, Lea, and DeVogelaere). No official common name.

***Sagamichthysabei*** Parr, 1953. **Shining Tubeshoulder**. Museum specimens: CAS-ICH 73324 (Monterey Bay, F Tsuji); MLMLF0217 (Monterey Bay, ME Anderson); SIO 87-16 (off Point Sur, R Snodgrass); SWFSC uncatalogued (Davidson Seamount during May 2015, RN Lea, EJ Burton, W Watson).

#### Order Osmeriformes

##### Family OSMERIDAE – smelts

***Allosmeruselongatus*** (Ayres, 1854). **Whitebait Smelt**. Museum specimens: CAS-ICH 46529 Half Moon Bay, M Hearne); CAS-SU 58426 (Monterey Bay, JC Briggs); USNM 86558 (Monterey, DS Jordan).

***Hypomesuspretiosus*** (Girard, 1854). **Surf Smelt**. Museum specimens: CAS-ICH 25756 (Santa Cruz Municipal Wharf, WI Follett); CAS-SU 58391 (Elkhorn Slough, JC Briggs).

***Spirinchusstarksi*** (Fisk, 1913). **Night Smelt**. Museum specimens: CAS-ICH 25470 (San Simeon, CL Hubbs, LC Hubbs, WI Follett et al.); CAS-ICH 212344 (off Point Montara, RL Bolin); MLMLF0221 (Moss Landing Harbor, GE Kukowski); CAS-SU 60470 (Monterey Bay, RL Bolin). Publication: Yoklavich et al. (1991) (Hwy 1 Bridge at Elkhorn Slough).

***Spirinchusthaleichthys*** (Ayres, 1860). **Longfin Smelt**. Museum specimens: ANSP 7726 (Monterey, HW Fowler); CAS-SU 5215 (SE of Santa Cruz, US Fish Commission).

***Thaleichthyspacificus*** (Richardson, 1837). **Eulachon**. Museum specimens: CAS-ICH 37473 (Monterey Bay, T Iwamoto); CAS-ICH 54942 (Monterey Bay, ME Anderson); MLMLF0226 (off Davenport at Monterey Bay, ME Anderson).

#### Order Salmoniformes

##### Family SALMONIDAE – trouts and salmons

***Oncorhynchusgorbuscha*** (Walbaum, 1792). **Pink Salmon**. Categorized as Historic. Scofield (1916) reported the following: several *O.gorbuscha* were collected from San Lorenzo River (Santa Cruz Co) in 1916; appears in San Lorenzo River only occasionally; is far out of natural range; strays have been taken in the Sacramento River. More recently, Moyle (2002) stated *O.gorbuscha* today are considered extremely rare in California and must be regarded as extirpated from the state.

***Oncorhynchusketa*** (Walbaum, 1792). **Chum Salmon**. Categorized as Historic. Scofield (1916) reported the following: three *O.keta* were collected from San Lorenzo River (Santa Cruz Co) in 1916; and have been reported from the Sacramento River. More recently, Moyle (2002) stated small runs of *O.keta* were historically present in streams from the Sacramento River north; a few fish are still taken in the Sacramento drainage, but no spawning has been recorded in recent decades; it has become increasingly rare in California, probably always uncommon; no recent records of chum salmon at northern Sacramento River drainage or San Joaquin drainage; considered endangered in California; and population in Sacramento River is extirpated.

***Oncorhynchuskisutch*** (Walbaum, 1792). **Coho Salmon**. Museum specimens: CAS-ICH 20840 (Scotts Creek, 1934, AC Jones); CAS-ICH 21044 and CAS-ICH 21048 (San Lorenzo River, 1955, AC Jones); CAS-ICH 210251 (Gazos Creek Lagoon, San Mateo County, 1970, WI Follett); CAS-ICH 210273 (Tunitas Creek, San Mateo County, 1939, JD Hopkirk); CAS-SU 4667 (Waddell Creek, 1895, Rutter and Scofield). Publication: Moyle (2002) [Central California coast (Scott and Waddell Creeks, Santa Cruz County)]. Although museum specimens were not collected within MBNMS, this is an anadromous species that must enter the ocean (adjacent MBNMS) during its life cycle. Central California Coast Coho Salmon were listed as threatened in 1996, and in 2005 reclassified as endangered (NOAA Fisheries 2016).

***Oncorhynchusmykiss*** (Walbaum, 1792). **Rainbow Trout (Steelhead)**. Museum specimens: CAS-ICH 23343 (Santa Cruz, RF Elwell); CAS-SU 15095 (Big Sur at Pfeiffer State Park, C Hubbs); USNM 27356 (Monterey, DS Jordan). Publication: Moyle (2002) [Central California coast steelhead (Aptos Creek); South/Central coast steelhead (Monterey Bay tributaries: Pajaro, Salinas, and Carmel Rivers; small streams of Big Sur coast; intermittent stream of San Luis Obispo)].

***Oncorhynchustshawytscha*** (Walbaum, 1792). **Chinook Salmon**. Museum specimens: CAS-ICH 225433 (Monterey Bay, DA Neely); MLMLF0230 (Monterey Bay, E Yarberry); CAS-SU 65851 (off Seaside, JB Phillips). Publications: Gilbert (1896) (Monterey Bay); Moyle (2002) [Central Valley fall-run (Sacramento and San Joaquin Rivers and tributaries); Central Valley spring-run (Sacramento River drainage); Sacramento River winter-run (mainstream Sacramento River)]. Important in recreational and commercial fisheries off central California coast.

#### Order Stomiiformes

##### Family GONOSTOMATIDAE – bristlemouths

***Cyclothoneacclinidens*** Garman, 1899. **Benttooth Bristlemouth**. Museum specimens: CAS-ICH 239350 (Davidson Seamount, RN Lea and EJ Burton); MLMLF0231 and MLMLF0232 (Monterey Bay, ME Anderson); SIO 67-102 (within Davidson Seamount Management Zone, CL Hubbs).

***Cyclothoneatraria*** Gilbert, 1905. **Black Bristlemouth**. Museum specimens: CAS-ICH 239321 and CAS-ICH 239351 (Davidson Seamount, RN Lea and EJ Burton); SIO 67-102 (within Davidson Seamount Management Zone, CL Hubbs).

***Cyclothonepallida*** Brauer, 1902. **Tan Bristlemouth**. Museum specimens: CAS-ICH 36750 (Monterey Bay, ME Anderson); CAS-ICH 97657 (Monterey Canyon, RL Bolin); CAS-ICH 97660 (off Point Pinos, RL Bolin).

***Cyclothonepseudopallida*** Mukhacheva, 1964. **Slender Bristlemouth**. Museum specimens: CAS-ICH 239322 and CAS-ICH 239352 (Davidson Seamount, RN Lea and EJ Burton); CAS-ICH 239362 (Sur Ridge, RN Lea and EJ Burton).

***Cyclothonesignata*** Garman, 1899. **Showy Bristlemouth**. Museum specimens: CAS-ICH 239353 (Davidson Seamount, RN Lea and EJ Burton); MLMLF0238 (Monterey Bay, GM Cailliet); SIO 67-102 (within Davidson Seamount Management Zone, CL Hubbs).

***Gonostomaatlanticum*** Norman, 1930. **Atlantic Fangjaw**. Museum specimen: CAS-ICH 239304 (Davidson Seamount, RN Lea and EJ Burton).

##### Family STERNOPTYCHIDAE – marine hatchetfishes

***Argyropelecusaffinis*** Garman, 1899. **Slender Hatchetfish**. Museum specimens: CAS-ICH 239354 (Davidson Seamount, RN Lea and EJ Burton); SIO 67-102 (within Davidson Seamount Management Zone, CL Hubbs).

***Argyropelecushemigymnus*** Cocco, 1829. **Spurred Hatchetfish**. Museum specimens: CAS-ICH 239331 and CAS-ICH 239355 (Davidson Seamount, RN Lea and EJ Burton); SIO 67-102 (within Davidson Seamount Management Zone, CL Hubbs).

***Argyropelecuslychnus*** Garman, 1899. **Tropical Hatchetfish**. Museum specimens: MLMLF0255 (Monterey Bay, ME Anderson); SWFSC uncatalogued (Sur Ridge during May 2015, W Watson).

***Argyropelecussladeni*** Regan, 1908. **Lowcrest Hatchetfish**. Museum specimens: CAS-ICH 239356 (Davidson Seamount, RN Lea and EJ Burton); MLMLF0258 (off Santa Cruz, B Antrim); SIO 67-112 (Monterey Canyon, CL Hubbs). Common name follows Hubbs et al. (1979).

***Danaphosoculatus*** (Garman, 1899). **Bottlelight**. Museum specimens: CAS-ICH 239295 and CAS-ICH 239357 (Davidson Seamount, RN Lea and EJ Burton); SIO 67-102 (within Davidson Seamount Management Zone, CL Hubbs).

***Sternoptyxdiaphana*** Hermann, 1781. **Longspine Hatchetfish**. Museum specimens: CAS-ICH 36778 (Monterey Bay, ME Anderson); CAS-ICH 54992 (Monterey Canyon, ME Anderson); MLMLF0265 (Monterey Bay, GM Cailliet).

***Sternoptyxobscura*** Garman, 1899. **Dusky Hatchetfish**. Museum specimens: CAS-ICH 239386 (Monterey Canyon, RN Lea and EJ Burton); MLMLF0268 (Monterey Bay, ME Anderson); CAS-SU 63852 (Monterey Canyon, TN Fast).

##### Family PHOSICHTHYIDAE – lightfishes

***Vinciguerrialucetia*** (Garman, 1899). **Panama Lightfish**. Museum specimen: MLMLF0278 (Monterey Bay, ME Anderson). Specimen re-examined by RN Lea, 2018; 29.5 mm SL.

##### Family STOMIIDAE – dragonfishes

***Aristostomiasscintillans*** (Gilbert, 1915). **Shiny Loosejaw**. Described from Monterey Bay. Museum specimens: Holotype (USNM 75808); CAS-ICH 239365 (Sur Ridge, RN Lea and EJ Burton); CAS-SU 58424 (off Hopkins Marine Station, JC Briggs); CAS-SU 64323 (Monterey Canyon, TN Fast). Publication: Gilbert (1915) (original description).

***Bathophilusflemingi*** Aron and McCrery, 1958. **Highfin Dragonfish**. Museum specimens: CAS-ICH 36762 (Monterey Bay, ME Anderson), CAS-ICH 56236 (off Point Sur, ME Anderson); CAS-SU 63582 (Monterey Canyon, ME Anderson).

***Chauliodusmacouni*** Bean, 1890. **Pacific Viperfish**. Museum specimens: CAS-ICH 36779 (Monterey Bay, ME Anderson); CAS-ICH 239333 (Davidson Seamount, RN Lea and EJ Burton); CAS-ICH 239366 (Sur Ridge, RN Lea and EJ Burton); SIO 67-102 (within Davidson Seamount Management Zone, CL Hubbs).

***Idiacanthusantrostomus*** Gilbert, 1890. **Pacific Blackdragon**. Museum specimens: CAS-ICH 36746 and CAS-ICH 36782 (Monterey Bay, ME Anderson); CAS-ICH 239316 (Davidson Seamount, RN Lea and EJ Burton); CAS-ICH 239367 (Sur Ridge, RN Lea and EJ Burton); SIO 67-102 (within Davidson Seamount Management Zone, CL Hubbs).

***Stomiasatriventer*** Garman, 1899. **Blackbelly Dragonfish**. Museum specimens: SIO 89-167 (south of Sur Ridge, identifier unknown, NMFS); SIO 78-115 (between Davidson Seamount Management Zone and MBNMS, 6.5 miles east of zone, identifier unknown, CalCOFI) included here due to close proximity of MBNMS and other record.

***Tactostomamacropus*** Bolin, 1939. **Longfin Dragonfish**. Described from off Monterey Bay. Museum specimens: Holotype (CAS-SU 33325); CAS-ICH 15012 (west of Big Sur, RN Lea); CAS-ICH 36740 (Monterey Bay, ME Anderson); CAS-ICH 239297 (Davidson Seamount, RN Lea and EJ Burton); SIO 67-102 (within Davidson Seamount Management Zone, CL Hubbs). Publication: Bolin (1939) (original description).

#### Order Aulopiformes

##### Family SYNODONTIDAE – lizardfishes

***Synoduslucioceps*** (Ayres, 1855). **California Lizardfish**. Museum specimens: CAS-ICH 18218 (Santa Cruz, R Dempster); CAS-ICH 79997 (Monterey Beach near Wharf #2, WC Freihofer); CAS-SU 12604 (Pacific Grove, H Heath).

##### Family NOTOSUDIDAE – waryfishes

***Scopelosaurusadleri*** (Fedorov, 1967). **Longfin Waryfish**. Listed with reservation. Museum specimens: SWFSC uncatalogued (Davidson Seamount during May 2015, W Watson, provisional identification; two specimens: 15.7-30.3 mm SL). Larger specimen identified as *S.adleri* according to pigment characters in Balanov and Savinykh (1999). Smaller specimen identified as *Scopelosaurus* sp.; pigment characters don’t quite fit those given by Balanov and Savinykh (1999) for either *S.adleri* or *S.harryi*, but are closest to *S.adleri*. This species has a broad distribution in the northern Pacific Ocean, including California (Balanov and Savinykh 1999). This is the first known record of occurrence within Monterey Bay National Marine Sanctuary (unpublished data, Burton, Lea, and DeVogelaere). Common name follows Mecklenburg et al. (2002).

***Scopelosaurusharryi*** (Mead, 1953). **Scaly Waryfish**. Museum specimens: CAS-ICH 36764 (Monterey Bay, ME Anderson); SIO 74-14 (within Davidson Seamount Management Zone, identifier unknown, CalCOFI).

##### Family SCOPELARCHIDAE – pearleyes

***Benthalbelladentata*** (Chapman, 1939). **Northern Pearleye**. Museum specimens: CAS-ICH 83254 (Monterey Canyon, ME Anderson); CAS-ICH 239368 (Sur Ridge, RN Lea and EJ Burton); SIO 67-112 (Monterey Canyon, CL Hubbs).

##### Family ALEPISAURIDAE – lancetfishes

***Alepisaurusferox*** Lowe, 1833. **Longnose Lancetfish**. Museum specimens: CAS-ICH 26078 (Half Moon Bay beach during May 1953, identifier unknown); CAS-ICH 26776 (Monterey Bay during May 1960, identifier unknown). Publications: Phillips (1930a) (Fan Shell Beach during Apr 1930, El Niño); Phillips (1931b) (Monterey during Oct 1930, El Niño); Phillips (1932c) (Monterey during 1931, El Niño). Records have coincided with El Niño events, but collections are likely due to beach cast events. According to Phillips (1930a) “This fish is taken only when it is forced above its natural deep sea strata.”

##### Family ANOTOPTERIDAE – daggerfishes

***Anotopterusnikparini*** Kukuev, 1998. **North Pacific Daggertooth**. Museum specimen: SIO 06-16 (Monterey Bay, KA Moots). Previously recognized in family Paralepididae (barracudinas). We follow Voskoboinikova and Nazarkin (2017), and current usage in *Catalog of Fishes* (Eschmeyer et al. 2017); with placement in family Anotopteridae.

##### Family PARALEPIDIDAE – barracudinas

***Arctozenusrisso*** (Bonaparte, 1840). **White Barracudina**. Museum specimens: CAS-SU 49270 (Monterey Bay, TN Fast); CAS-SU 49271 (Monterey Canyon, RL Bolin).

***Lestidiopsringens*** (Jordan & Gilbert, 1880). **Slender Barracudina**. Museum specimens: MLMLF0316 (Monterey Bay, ME Anderson); SIO 67-112 (Monterey Canyon, CL Hubbs); SIO 74-14 (within Davidson Seamount Management Zone, identifier unknown, CalCOFI).

***Lestidiopssphyraenopsis*** Hubbs, 1916. **Smalleye Barracudina**. Museum specimens: CAS-ICH 18728 (Half Moon Bay, L Dempster); CAS-SU 35171 (10 miles south of Point Sur, identifier unknown, Templeton Crocker Expedition). Common name follows Hubbs et al. (1979).

***Magnisudisatlantica*** (Krøyer, 1868). **Duckbill Barracudina**. Museum specimen: CAS-ICH 41868 (Monterey Wharf #2, JB Phillips).

##### Family BATHYSAURIDAE – deep-sea lizardfishes

***Bathysaurusmollis*** Günther, 1878. **Highfin Lizardfish**. Publication: Burton and Lundsten (2008) (Davidson Seamount, based on imagery). Visual records: MBARI/NOAA T426-08, MBARI/NOAA T427-07 and MBARI/NOAA T427-10 (Davidson Seamount, Jacobson Stout et al. 2017). Previously recognized in family Synodontidae (lizardfishes). We follow Sato and Nakabo (2002), Russell (2003), and current usage in *Catalog of Fishes* (Eschmeyer et al. 2017), with placement in family Bathysauridae.

#### Order Myctophiformes

##### Family MYCTOPHIDAE – lanternfishes

***Ceratoscopelustownsendi*** (Eigenmann & Eigenmann, 1889). **Dogtooth Lampfish**. Museum specimens: CAS-ICH 239298 and CAS-ICH 239334 (Davidson Seamount, RN Lea and EJ Burton); SIO 67-102 (within Davidson Seamount Management Zone, CL Hubbs).

***Diaphustheta*** Eigenmann and Eigenmann, 1890. **California Headlightfish**. Museum specimens: CAS-ICH 239307 (Davidson Seamount, RN Lea and EJ Burton); CAS-ICH 239369 (Sur Ridge, RN Lea and EJ Burton); CAS-ICH 239389 (Monterey Canyon, RN Lea and EJ Burton); SIO 67-102 (within Davidson Seamount Management Zone, CL Hubbs).

***Diogenichthysatlanticus*** (Tåning, 1928). **Longfin Lanternfish**. Museum specimens: CAS-ICH 239336 (Davidson Seamount, RN Lea and EJ Burton); SIO 74-14 (within Davidson Seamount Management Zone, identifier unknown, CalCOFI); CAS-SU 63873 (Monterey Canyon, TN Fast).

***Lampanyctussteinbecki*** Bolin, 1939. **Longfin Lampfish**. Museum specimens: SIO 74-14 (within Davidson Seamount Management Zone during Aug 1970, identifier unknown, CalCOFI); SIO 89-182 (outside Monterey Bay during Aug 1972, identifier unknown, NMFS); SIO 89-184 (outside Monterey Bay during Aug 1972, identifier unknown, NMFS).

***Lampanyctustenuiformis*** (Brauer, 1906). **lanternfish**. Listed with reservation. Museum specimens: SIO 89-165, SIO 89-167, SIO 89-168 and SIO 96-193 (west of Big Sur, identifier unknown, NMFS). Wisner (1976) indicates *L.tenuiformis* is apparently widespread in the eastern Pacific Ocean, but with few specimens taken is either uncommon or not captured. A large number of SIO specimens are available; however, we consider suspect due to rarity. We tentatively include here, understanding specimens may be misidentified. No official common name.

***Nannobrachiumregale*** (Gilbert, 1892). **Pinpoint Lampfish**. Museum specimens: MLMLF0389 (Monterey Bay, GM Cailliet); SIO 67-102 (within Davidson Seamount Management Zone, CL Hubbs); SWFSC uncatalogued (Sur Ridge during May 2015, W Watson).

***Nannobrachiumritteri*** (Gilbert 1915). **Broadfin Lampfish**. Described from Monterey Bay. Museum specimens: Holotype (USNM 75807); CAS-ICH 239308 (Davidson Seamount, RN Lea and EJ Burton); CAS-ICH 239370 (Sur Ridge, RN Lea and EJ Burton); SIO 67-102 (within Davidson Seamount Management Zone, CL Hubbs). Publication: Gilbert (1915) (original description).

***Parviluxingens*** Hubbs and Wisner, 1964. **Giant Lampfish**. Museum specimens: CAS-ICH 63859 (Monterey Canyon, RL Bolin); MLMLF0397 (Monterey Bay, GM Cailliet); SIO 87-16 (off Point Sur, R Snodgrass).

***Protomyctophumcrockeri*** (Bolin, 1939). **California Flashlightfish**. Museum specimens: CAS-ICH 239326 (Davidson Seamount, RN Lea and EJ Burton); CAS-ICH 239377 (Monterey Bay, RN Lea and EJ Burton); SIO 67-102 (within Davidson Seamount Management Zone, CL Hubbs).

***Protomyctophumthompsoni*** (Chapman, 1944). **Northern Flashlightfish**. Museum specimen: CAS-ICH 203156 (Monterey Canyon, RL Bolin).

***Stenobrachiusleucopsarus*** (Eigenmann & Eigenmann, 1890) **Northern Lampfish**. Museum specimens: CAS-ICH 239359 (Davidson Seamount, RN Lea and EJ Burton); CAS-ICH 239371 (Sur Ridge, RN Lea and EJ Burton); CAS-ICH 239378 (Monterey Bay, RN Lea and EJ Burton); SIO 67-102 (within Davidson Seamount Management Zone, CL Hubbs).

***Symbolophoruscaliforniensis*** (Eigenmann & Eigenmann, 1889). **California Lanternfish**. Museum specimens: CAS-ICH 239301 (Davidson Seamount, RN Lea and EJ Burton); MLMLF0412 (Monterey Canyon, ME Anderson); SIO 89-174 (offshore Monterey Bay, identifier unknown, NMFS). Common name follows Hubbs et al. (1979).

***Taaningichthyspaurolychnus*** Davy, 1972. **Dimlight Lampfish**. Museum specimens: Paratypes (SIO 67-102, within Davidson Seamount Management Zone, CL Hubbs).

***Tarletonbeaniacrenularis*** (Jordan & Gilbert, 1880). **Blue Lanternfish**. Museum specimens: CAS-ICH 239392 (Monterey Canyon, RN Lea and EJ Burton); MLMLF0420 (Monterey Bay, D Varoujean); SIO 67-102 (within Davidson Seamount Management Zone, CL Hubbs).

***Triphoturusmexicanus*** (Gilbert, 1890). **Mexican Lampfish**. Museum specimens: CAS-ICH 239360 (Davidson Seamount, RN Lea and EJ Burton); CAS-ICH 239372 (Sur Ridge, RN Lea and EJ Burton); CAS-SU 66290 (Monterey Canyon, RL Bolin).

#### Order Lampridiformes

##### Family LAMPRIDAE – opahs

***Lamprisincognitus*** (Underkoffler, Luers, Hyde, and Craig, 2018). **Smalleye Pacific Opah**. Museum specimens: CAS-ICH 29716 (off Princeton, Half Moon Bay, identifier unknown, Aug 1973, El Niño); CAS-ICH 53013 (WSW of Point Piedras Blancas, identifier unknown, Oct 1964, moderate El Niño); CAS-ICH 53015 (off Half Moon Bay, identifier unknown, Aug 1973, El Niño). Publication: Radovich (1961) (off Monterey during 1957 and 1959, El Niño). Categorized as occurring during warm-water events (e.g., El Niño). Based on recent genetic, morphological, and meristic data analyses, recent publications propose a taxonomic revision of *Lamprisguttatus* (Brünnich, 1788), including a description of the new species *Lamprisincognitus* (Hyde et al. 2014, Underkoffler et al. 2018). We follow Underkoffler et al. (2018), where *Lamprisincognitus* is the only known opah species occurring in the Eastern North Pacific. Previous to this revision, California records have been reported as *Lamprisregius* and *Lamprisguttatus*.

##### Family TRACHIPTERIDAE – ribbonfishes

***Desmodemalorum*** Rosenblatt and Butler, 1977. **Whiptail Ribbonfish**. Museum specimen: SIO 74-14 (within Davidson Seamount Management Zone, identifier unknown, CalCOFI). Visual record: MBARI D668-01 (2 miles north of Davidson Seamount Management Zone, Jacobson Stout et al. 2017) included here due to close proximity of MBNMS and other record.

***Trachipterusaltivelis*** Kner, 1859. **King-of-the-salmon**. Museum specimens: CAS-ICH 49067 (Pacific Grove during Oct 1981, WN Eschmeyer); CAS-ICH 58607 (Davenport during Jun 1986, identifier unknown, NMFS); MLMLF0433 (Monterey Breakwater during Jan 1971, GE Kukowski); CAS-SU 13080 (Monterey beach during Jun 1907, JO Snyder). Publication: Phillips (1932d) (Monterey Bay during 1931, El Niño).

#### Order Gadiformes

##### Family MACROURIDAE – grenadiers

***Coelorinchusscaphopsis*** (Gilbert, 1890). **Shoulderspot Grenadier**. Museum specimen: SIO 88-127 (off Cambria, HG Moser).

***Coryphaenoidesacrolepis*** (Bean, 1884). **Pacific Grenadier**. Museum specimens: CAS-ICH 18780 (off Santa Cruz, R Dempster); CAS-ICH 42566 (off Cypress Point, RN Lea); CAS-ICH 65080 (Monterey Bay, T Iwamoto); CAS-SU 17980 (off Point Lobos, RL Bolin). Publication: Burton and Lundsten (2008) (Davidson Seamount, based on imagery). Visual records: MBARI/NOAA T428-04 and MBARI/NOAA D944-04 (Davidson Seamount, EJ Burton, L Kuhnz, and L Lundsten; Jacobson Stout et al. 2017).

***Coryphaenoidesarmatus*** (Hector, 1875). **Abyssal Grenadier**. Publication: Burton and Lundsten (2008) (Davidson Seamount, based on imagery). Visual record: MBARI/NOAA T427-03 (Davidson Seamount, EJ Burton and L Kuhnz).

***Coryphaenoidescinereus*** (Gilbert, 1896). **Popeye Grenadier**. Museum specimens: UW 41685 (near Monterey Bay, Hoff 1999); UW 113725 (Monterey Bay, J Hoff). Publication: Hoff (1999) (near Monterey Bay, UW 41685). Common name follows Cohen et al. (1990).

***Coryphaenoidesfilifer*** (Gilbert, 1896). **Threadfin Grenadier**. Publication: Burton and Lundsten (2008) (Davidson Seamount, based on imagery). Visual record: MBARI/NOAA T426-04 (Davidson Seamount, EJ Burton and L Kuhnz; Jacobson Stout et al. 2017). Common name follows Hubbs et al. (1979).

***Coryphaenoidesleptolepis*** Günther, 1877. **Ghostly Grenadier**. Publication: Burton and Lundsten (2008) (Davidson Seamount, based on imagery). Visual records: MBARI/NOAA T427-04 and MBARI/NOAA T945-02 (Davidson Seamount, EJ Burton and L Kuhnz; Jacobson Stout et al. 2017).

***Coryphaenoidespectoralis*** (Gilbert, 1892). **Giant Grenadier**. Museum specimens: CAS-ICH 34356 (Monterey Bay, T Iwamoto); CAS-ICH 51589 (Santa Cruz, RN Lea); MLMLF0459 (Monterey Bay, ME Anderson). Visual records: MBARI/NOAA T943-03 (Davidson Seamount, EJ Burton and T Iwamoto); MBARI D621 (Sur Ridge, EJ Burton and L Kuhnz). Previously recognized as *Albatrossiapectoralis*. We follow Wilson (1994), Morita (1999), Wilson and Attia (2003), and Gaither et al. (2016), with placement in *Coryphaenoides*.

***Malacocephaluslaevis*** (Lowe, 1843). **Softhead Grenadier**. Museum specimens: CAS-ICH 55884 (San Simeon, T Iwamoto); UW 41690 (near Point Sur, Hoff 1999). Publication: Hoff (1999) (near Point Sur, UW 41690). Common name follows Cohen et al. (1990).

***Nezumialiolepis*** (Gilbert, 1890). **Smooth Grenadier**. Museum specimen: CAS-SU 5351 (Cabrillo Canyon, SW of Santa Cruz, identifier unknown, US Fish Commission). Publication: Hoff et al. (2000) (outside Monterey Bay).

***Nezumiastelgidolepis*** (Gilbert, 1890). **California Grenadier**. Museum specimens: CAS-ICH 30457 (Monterey Bay, ME Anderson); CAS-ICH 31509 (off Point San Simeon, WI Follett); CAS-ICH 32309 (off Pigeon Point, T Iwamoto). Publication: Hoff et al. (2000) (off Cape San Martin).

##### Family MORIDAE – codlings

***Antimoramicrolepis*** Bean, 1890. **Pacific Flatnose**. Museum specimens: CAS-ICH 34353 and CAS-ICH 34354 (off Monterey Bay, ME Anderson); CAS-SU 5276 (off Monterey Bay, identifier unknown, US Fish Commission). Publication: Burton and Lundsten (2008) (Davidson Seamount, based on imagery). Visual record: MBARI/NOAA T427-05 (Davidson Seamount, L Kuhnz).

***Halargyreusjohnsonii*** Günther, 1862. **Slender Codling**. Museum specimen: UW 48729 (east of Sur Ridge, RC Harrison). Common name follows Cohen et al. (1990).

***Physiculusnematopus*** Gilbert, 1890. **Charcoal Codling**. Museum specimen: CAS-ICH 241419 (head of Monterey Canyon at Monterey Bay, RN Lea). This account documents the first verifiable record of this species from outside the Panamic biogeographic province. The specimen was collected by the California Department of Fish and Wildlife, F/V *Donna Kathleen*, on 25 Mar 2013, using fish traps while conducting juvenile rockfish surveys in Monterey Bay. The fish was given to RNL for identification as an unusual capture. Upon examination it was obvious as *Physiculus* and would have been easy to assume it as *Physiculusrastrelliger*, Hundred-fathom Codling; the species known from the eastern North Pacific and occasionally encountered off California. However, during routine analysis it became apparent that it was not *P.rastrelliger* and was in fact *P.nematopus*. Data relating to the specimen including characteristics distinguishing it from *P.rastrelliger*: 173 mm TL; 155 mm SL; 62.1 g; caudal area injured, hence second dorsal fin, anal fin soft-ray, and caudal fin counts were not possible – confirmed by x-ray; first Dorsal fin IX; Pectoral fin left 24; Pelvic fins 6, filamentous (7 in *P.rastrelliger*); Gill rakers left 3+13=16 (18-22 on lower limb and 26 to 30 total in *P.rastrelliger*). Paulin (1989) provided a review of the genus *Physiculus*.

***Physiculusrastrelliger*** Gilbert, 1890. **Hundred-fathom Codling**. Museum specimens: MLMLF0486 (Monterey Bay, ME Anderson); SIO 09-200 (Monterey Canyon, identifier unknown).

##### Family MERLUCCIIDAE – merlucciid hakes

***Merlucciusproductus*** (Ayres, 1855). **Pacific Hake**. Museum specimens: CAS-ICH 26079 (Half Moon Bay, WI Follett); CAS-ICH 81648 (Monterey Canyon, EH Ahlstrom); CAS-SU 65856 (Monterey Canyon, TN Fast).

##### Family GADIDAE – cods

***Gaduschalcogrammus*** Pallas, 1814. **Walleye Pollock**. Publications: Phillips (1942a) (Monterey Bay); Phillips (1943b) (off Carmel). Visual records: unpublished data (landed at Moss Landing during 1986, 588 mm TL, RN Lea); unpublished data (off Santa Cruz during 1996, 165 fathoms, 625 mm TL, RN Lea); unpublished data (south of Lopez Point during 1997, 220 fathoms, 550 mm TL, RN Lea).

***Gadusmacrocephalus*** Tilesius, 1810. **Pacific Cod**. Museum specimens: MLMLF0467 and MLMLF0468 (Monterey Bay, E Osada). Publications: Phillips (1951a) (off Point Sur), Phillips (1953) (Monterey area).

***Microgadusproximus*** (Girard, 1854). **Pacific Tomcod**. Museum specimens: CAS-ICH 25447 (Half Moon Bay, WI Follett); MLMLF0469 (off Pajaro River at Monterey Bay, L Talent); UMMZ 56377 (Monterey Bay, WL Scofield).

#### Order Ophidiiformes

##### Family OPHIDIIDAE – cusk-eels

***Chilarataylori*** (Girard, 1858). **Spotted Cusk-eel**. Described from Monterey. Museum specimens: Syntypes [MCZ 35928, UMMZ 146978, USNM 867]; CAS-ICH 31773 (off Cambria, JB Phillips); CAS-ICH 81604 (Monterey, RL Bolin); CAS-ICH 236552 (Del Monte Beach, RN Lea); CAS-SU 64112 (Davidson Seamount, 82 mm TL nektonic prejuvenile, specimen likely spit up by Albacore, collected by fisherman, RN Lea). Publications: Girard (1858a) (original description); Cailliet et al. (1977) (Hwy 1 Bridge at Elkhorn Slough).

***Lamprogrammusniger*** Alcock, 1891. **Paperbone Cusk-eel**. Museum specimen: UW 47396 (canyon south of Pioneer Canyon, J Hoff).

***Luciobrotula* sp. A**. **cusk-eel.** Publications: Burton and Lundsten (2008) (Davidson Seamount, based on imagery); Burton et al. (2017) (Sur Ridge, based on imagery). Visual records: MBARI T0357-02 and MBARI T0611-02 (Monterey Canyon, Jacobson Stout et al. 2017); MBARI D0981-02 (Sur Ridge, Burton et al. 2017); MBARI/NOAA T0430-11 (Davidson Seamount, Burton and Lundsten 2008). Taxonomist assessment of MBARI visual records in Aug 2012 by Jørgen G. Nielsen (retired, University of Copenhagen); ‘closest recorded species in this genus is from Panama, so these are probably an undescribed species’ (Jacobson Stout et al. 2017). No official common name.

***Spectrunculusgrandis*** (Günther, 1877). **Giant Cusk-eel**. Publications: Burton and Lundsten (2008), and Lundsten et al. (2009) (Davidson Seamount, based on imagery). Visual records: MBARI/NOAA T946-01, MBARI/NOAA T946-03, MBARI/NOAA T946-04 and MBARI/NOAA T947-01 (Davidson Seamount, EJ Burton, GM Cailliet and L Lundsten).

##### Family BYTHITIDAE – viviparous brotulas

***Brosmophycismarginata*** (Ayres, 1854). **Red Brotula**. Museum specimens: CAS-ICH 80641 (off Half Moon Bay, B Breen); SIO 89-194 (west of Monterey Bay, identifier unknown, NMFS).

***Cataetyxrubrirostris*** Gilbert, 1890. **Rubynose Brotula**. Museum specimens: CAS-ICH 36749, CAS-ICH 36771, and CAS-ICH 36792 (Monterey Bay, ME Anderson). Publications: Anderson et al. (1979) (Monterey Bay); Gibbs (1991) (Monterey and Carmel Canyons).

#### Order Batrachoidiformes

##### Family BATRACHOIDIDAE – toadfishes

***Porichthysnotatus*** Girard, 1854. **Plainfin Midshipman**. Museum specimens: CAS-ICH 23406 (Santa Cruz Wharf, RN Lea); CAS-ICH 233552 (Monterey Bay, RR Rofen); CAS-SU 58446 (Elkhorn Slough, JC Briggs). Publication: Yoklavich et al. (1991) (Hwy 1 Bridge, Dairy, Kirby Park, and Hudson’s Landing at Elkhorn Slough).

#### Order Lophiiformes

##### Family LOPHIIDAE – goosefishes

***Lophiodesspilurus*** (Garman, 1899). **Threadfin Goosefish**. Museum specimen: LACM 43535.001 (SW of Santa Cruz during 1983, El Niño, RN Lea). Publication: Lea et al. (1984) (SW of Santa Cruz during 1983, El Niño, second California record). Categorized as occurring during warm-water events (e.g., El Niño).

##### Family CHAUNACIDAE – gapers

***Chaunacopscoloratus*** (Garman, 1899). **gaper**. Museum specimen: CAS-ICH 216055 (Davidson Seamount, GM Cailliet and HC Ho). Publications: Burton and Lundsten (2008) (Davidson Seamount, CAS-ICH 216055); Lundsten et al. (2012) (Davidson Seamount, CAS-ICH 216055). No official common name.

##### Family MELANOCETIDAE – blackdevils

***Melanocetusjohnsonii*** Günther, 1864. **Blackdevil**. Museum specimen: UW 48693 (Sur Canyon, identifier unknown, NMFS). Visual record: MBARI D695 (Monterey Bay, Jacobson Stout et al. 2017). MBARI visual record resolved to genus; however, *M.johnsonii* is the only known species off California (Fitch and Lavenberg 1968, Love et al. 2005).

##### Family HIMANTOLOPHIDAE – footballfishes

***Himantolophusnigricornis*** Bertelsen and Krefft, 1988. **footballfish**. Museum specimen: SIO 94-71 (off Point Año Nuevo, RN Lea). No official common name.

***Himantolophussagamius*** (Tanaka, 1918). **Pacific Footballfish**. Museum specimen: CAS-ICH 57639 (Monterey Canyon, E Bertelsen). Publication: Lea (1988) (Monterey Canyon, CAS-ICH 57639).

##### Family ONEIRODIDAE – dreamers

***Chaenophrynelongiceps*** Regan, 1925. **dreamer**. Museum specimens: SIO 98-115 (Monterey Bay, RN Lea); USNM 150087 (off Point Pinos, identifier unknown, “Albatross” Explorations of the California Coast, 1904). No official common name.

***Chaenophrynemelanorhabdus*** Regan and Trewavas, 1932. **Smooth Dreamer**. Museum specimens: SIO 96-75 off (Moss Landing, B Leos); SIO 97-128 (south of Point Sur, identifier unknown, CDFG). Common name follows Mecklenburg et al. (2002).

##### Family CERATIIDAE – seadevils

***Cryptopsarascouesii*** Gill, 1883. **Triplewart Seadevil**. Museum specimen: CAS-ICH 73320 (Monterey Canyon during Aug 1990, M Innes). Publication: Fast (1957) (Monterey Bay during Sept 1956, warm-water event). Categorized as occurring during warm-water events (e.g., El Niño).

#### Order Mugiliformes

##### Family MUGILIDAE – mullets

***Mugilcephalus*** Linnaeus, 1758. **Striped Mullet**. Museum specimens: SBMNH 1800 (Marina Dunes, identifier unknown); USNM 26796 (Monterey, DS Jordan). Visual records: unpublished data (Monterey Wharf #2 during Oct 1987, K Oda); unpublished data (Monterey Wharf #2 during Oct 2001, 545 mm TL, 1880.2 g, RN Lea). Found in warm seas (Miller and Lea 1972), rare north of Point Conception (Moyle 2002); and found in San Francisco Bay and as far north as Humboldt Bay (Wallace et al. 2015) during warm water years (e.g. El Niño). Categorized as occurring during warm-water events (e.g., El Niño).

#### Order Atheriniformes

##### Family ATHERINOPSIDAE – New World silversides

***Atherinopsaffinis*** (Ayres, 1860). **Topsmelt**. Museum specimens: CAS-ICH 45119 (southern Monterey Bay, D Wilson); CAS-SU 55244 (Elkhorn Slough, WC Freihofer); CAS-SU 58401 (Elkhorn Slough, JC Briggs). Publication: Yoklavich et al. (1991) (Hwy 1 Bridge, Dairy, Kirby Park, and Hudson’s Landing at Elkhorn Slough).

***Atherinopsiscaliforniensis*** Girard, 1854. **Jacksmelt**. Museum specimens: MLMLF0546 (near Dairy at Elkhorn Slough, GE Kukowski); CAS-SU 47508 (Pacific Grove, WC Freihofer); CAS-SU 58399 (Elkhorn Slough, JC Briggs). Publication: Yoklavich et al. (1991) (Hwy 1 Bridge, Dairy, and Kirby Park at Elkhorn Slough).

***Leuresthestenuis*** (Ayres, 1860). **California Grunion**. Museum specimens: CAS-ICH 42072 (New Brighton State Beach, Santa Cruz County, JD Spratt); CAS-ICH 54825 (Moss Landing during 1983, El Niño, GM Cailliet); SIO 45-56 (off Del Monte Beach, CL Hubbs). Publications: Thompson (1920) (Monterey); Phillips (1943a) (Monterey during 1942); Radovich (1961) (Monterey during 1959, El Niño); Spratt (1981) (New Brighton State Beach, Santa Cruz County, CAS-ICH 42072). Categorized as occurring during warm-water events (e.g., El Niño).

#### Order Beloniformes

##### Family EXOCOETIDAE – flyingfishes

***Cheilopogonpinnatibarbatus*** (Bennett, 1831). **Smallhead Flyingfish**. Publication: Cooper (1863) (to at least Santa Cruz). Visual records: Observed during Aug 1986 off Point Joe by John Stern (San Francisco State University graduate student, reported to Alan Baldridge, Hopkins Marine Station); three sightings observed from Cypress Point and Point Sur areas during Jun 1992 (warm-water event) by Richard Ternullo (skipper of PT SUR CLIPPER, reported to Alan Baldridge, Hopkins Marine Station); observed 2.5 miles off Cypress Point during Jul 1994 (warm-water event) by Nancy Black (Oceanic Society Expeditions, reported to RN Lea). Categorized as occurring during warm-water events (e.g., El Niño).

##### Family BELONIDAE – needlefishes

***Strongyluraexilis*** (Girard, 1854). **California Needlefish**. Museum specimen: CAS-ICH 64942 (San Francisco Bay, near Golden Gate Bridge during Apr 1986, moderate El Niño, identifier unknown). Publication: Phillips (1940) (“short distance off Monterey”). Categorized as occurring during warm-water events (e.g., El Niño).

##### Family SCOMBERESOCIDAE – sauries

***Cololabissaira*** (Brevoort, 1856). **Pacific Saury**. Museum specimens: CAS-ICH 18215 (Half Moon Bay, RR Harry); CAS-ICH 25629 (off Waddell Creek during 1950, WI Follett); MLMLF0555 (Monterey Bay during Nov 1970, L Talent). Visual record: SIMoN (2018) (Davidson Seamount during Jul 2018, EJ Burton). Publication: Phillips (1932a) (Monterey Bay during 1931, El Niño).

#### Order Stephanoberyciformes

##### Family MELAMPHAIDAE – bigscales

***Melamphaeslugubris*** Gilbert, 1890. **Highsnout Bigscale**. Museum specimens: CAS-ICH 36796 (Monterey Bay, ME Anderson); SIO 67-102 (within Davidson Seamount Management Zone, CL Hubbs); SWFSC uncatalogued (Davidson Seamount during May 2015, W Watson). We collected a larval specimen of *Melamphaes* sp. from Davidson Seamount during 2015, that was provisionally identified by W Watston (NOAA Fisheries, SWFSC) as Melamphaescf.parvus. Identification follows Sandknop and Watson (1996). Specimen may be *Melamphaesparvus*; however, not in good enough condition to be certain. This species is known to occur off California (Ebeling 1962, Sandknop and Watson 1996, Kotlyar 2004). This provisional identification of *M.parvus* is not included in the total number of species in MBNMS. Common name follows Hubbs et al. (1979).

***Poromitracrassiceps*** (Günther, 1878). **Crested Bigscale**. Museum specimens: CAS-ICH 36752 (Monterey Bay, ME Anderson); CAS-ICH 239361 (Davidson Seamount, RN Lea and EJ Burton); SIO 67-102 (within Davidson Seamount Management Zone, CL Hubbs). Common name follows Hubbs et al. (1979).

***Scopelogadusbispinosus*** (Gilbert, 1915). **Twospine Bigscale**. Museum specimens: CAS-ICH 36735 and CAS-ICH 36790 (Monterey Bay, ME Anderson); SIO 67-102 (within Davidson Seamount Management Zone, CL Hubbs). Common name follows Hubbs et al. (1979).

#### Order Beryciformes

##### Family ANOPLOGASTRIDAE – fangtooths

***Anoplogastercornuta*** (Valenciennes, 1833). **Fangtooth**. Museum specimens: CAS-ICH 55413 (off Point Sur, ME Anderson); SIO 88-99 (south of Point Sur, identifier unknown, NMFS).

#### Order Zeiformes

##### Family OREOSOMATIDAE – oreos

***Allocyttusfolletti*** Myers, 1960. **Oxeye Oreo**. Museum specimens and Publication: CAS-ICH 39087 (off Cypress Point, Anderson et al. 1979); CAS-ICH 39088 (off Point Sur, Anderson et al. 1979).

##### Family ZEIDAE – dories

***Zenopsisnebulosa*** (Temminck & Schlegel, 1845). **Mirror Dory**. Museum specimen: CAS-ICH 24164 (Monterey Bay, P Gregory).

#### Order Gasterosteiformes

##### Family AULORHYNCHIDAE – tubesnouts

***Aulorhynchusflavidus*** Gill, 1861. **Tubesnout**. Museum specimens: CAS-SU 35360 (Point Cabrillo at Monterey Bay, RL Bolin); CAS-SU 35361 (Monterey Bay, RL Bolin); CAS-SU 58405 (Carmel Beach, JC Briggs).

##### Family GASTEROSTEIDAE – sticklebacks

***Gasterosteusaculeatus*** Linnaeus, 1758. **Threespine Stickleback**. Museum specimens: OSUM 71680 (Elkhorn Slough, G Varney and G Varney, Jr); CAS-SU 15026 (upper Elkhorn Slough, HW Freeman and IL Firschein). Publication: Yoklavich et al. (1991) (station location from Barry 1983, Hudson’s Landing at Elkhorn Slough)

##### Family SYNGNATHIDAE – pipefishes

***Cosmocampusarctus*** (Jenkins & Evermann, 1889). **Snubnose Pipefish**. Museum specimens: SIO 11-335 (Elkhorn Slough, RN Lea); SIO 45-53 (Elkhorn Slough, CL Hubbs and RL Bolin).

***Syngnathuscaliforniensis*** Storer, 1845. **Kelp Pipefish**. Museum specimens: CAS-SU 35362 (Monterey Bay, RL Bolin); CAS-SU 36462 (Elkhorn Slough, ES Herald and WI Follett); CAS-SU 58461 (Hopkins Marine Station, JC Briggs).

***Syngnathusexilis*** (Osburn & Nichols, 1916). **Barcheek Pipefish**. Museum specimens: SIO 45-56 (Monterey Bay, Hubbs), SIO 92-135 (Elkhorn Slough, RN Lea and L Allen).

***Syngnathusleptorhynchus*** Girard, 1854. **Bay Pipefish**. Museum specimens: SIO 45-53 and SIO 62-510 (Elkhorn Slough, CL Hubbs); SIO 93-189 (Elkhorn Slough, RN Lea). Publication: Yoklavich et al. (1991) (Hwy 1 Bridge and Dairy at Elkhorn Slough).

#### Order Scorpaeniformes

##### Family SCORPAENIDAE – scorpionfishes

***Scorpaenaguttata*** Girard, 1854. **California Scorpionfish**. Described from Monterey Bay. Museum specimens: Holotype (USNM 350); MLMLF0613 (off Santa Cruz at Monterey Bay during 1970, L Talent and D Varoujean). Publications: Girard (1854) (original description); Varoujean (1972) (3 miles off Point Santa Cruz, MLMLF0613). Visual record: Observed and collected live at 2 miles from Moss Landing jetty on south side of Monterey Canyon during Dec 1984 by Bill Frogue (commercial fisherman, reported and delivered to Dave Powell for display at Monterey Bay Aquarium).

***Sebastesatrovirens*** (Jordan & Gilbert, 1880). **Kelp Rockfish**. Museum specimens: CAS-ICH 25901 (Monterey Bay, WI Follett); CAS-ICH 47396 (kelp bed off Lovers Point, RN Lea); CAS-ICH 56343 (Monterey Breakwater, W Laroche). Publications: Cailliet et al. (1977) (Dairy at Elkhorn Slough); Lea (1983) (Monterey Bay to San Simeon).

***Sebastesauriculatus*** Girard, 1854. **Brown Rockfish**. Museum specimens: CAS-ICH 14803 (Monterey Bay, DW Behrens); CAS-ICH 40643 (Elkhorn Slough, RL Bolin); CAS-ICH 56272 (Half Moon Bay, W Laroche). Publication: Yoklavich et al. (1991) (Hwy 1 Bridge, Dairy, and Kirby Park at Elkhorn Slough).

***Sebastesaurora*** (Gilbert, 1890). **Aurora Rockfish**. Museum specimens: CAS-ICH 37500 (west of Pigeon Point, T Iwamoto); CAS-ICH 39893 (off Yankee Point, WN Eschmeyer and T Iwamoto); CAS-ICH 40033 (NW of Point Pinos, WC Ruark).

***Sebastesbabcocki*** (Thompson, 1915). **Redbanded Rockfish**. Museum specimens: CAS-ICH 30468 (Monterey Bay, ME Anderson); CAS-ICH 30735 (west of Moss Landing Marine Laboratories, ME Anderson); CAS-ICH 38724 (Monterey Bay, WA Laroche).

***Sebastesborealis*** Barsukov, 1970. **Shortraker Rockfish**. Museum specimen: SIO 01-186 (between Carmel Bay and Big Sur, RN Lea).

***Sebastesbrevispinis*** (Bean, 1884). **Silvergray Rockfish**. Museum specimens: CAS-ICH 25997 (off Point Sur, identifier unknown); CAS-SU 47300 (off Point Sur, L Schultz).

***Sebastescarnatus*** (Jordan & Gilbert, 1880). **Gopher Rockfish**. Described from Monterey Bay. Museum specimens: Syntypes [ANSP 12190 (1), USNM 26993 (5)]; CAS-ICH 14735 (Carmel River Beach, L Hallacher); CAS-ICH 27697 (off Half Moon Bay, WN Eschmeyer). Publication: Jordan and Gilbert (1880c) (original description).

***Sebastescaurinus*** Richardson, 1844. **Copper Rockfish**. Museum specimens: CAS-ICH 14734 (Monastery Beach, L Hallacher); CAS-ICH 27356 (Del Monte kelp bed at Monterey Bay, DC Powell); CAS-ICH 40641 (Elkhorn Slough, WA Laroche). Publication: Cailliet et al. (1977) (Hwy 1 Bridge and Dairy at Elkhorn Slough).

***Sebasteschlorostictus*** (Jordan & Gilbert, 1880). **Greenspotted Rockfish**. Described from deep water at Monterey; obtained at San Francisco market. Museum specimens: Syntypes (numerous specimens, including: ANSP 12126, USNM 26964, USNM 27092); CAS-ICH 25991 (off Half Moon Bay, WI Follett); CAS-ICH 47400 (off Point Piedras Blancas, RN Lea). Publication: Jordan and Gilbert (1880i) (original description).

***Sebasteschrysomelas*** (Jordan & Gilbert, 1881). **Black-and-yellow Rockfish**. Described from Monterey Bay. Museum specimens: Syntypes [numerous specimens including: ANSP 12127 (1), USNM 26968 (4)]; CAS-SU 15122 (Soberanes Point, C Hubbs); CAS-ICH 51863 (just south of Point Sur, RN Lea). Publication: Jordan and Gilbert (1881) (original description).

***Sebastesconstellatus*** (Jordan & Gilbert, 1880). **Starry Rockfish**. Museum specimens: CAS-ICH 84359 (near Monterey, D Montgomery); CAS-SU 3236 (Pacific Grove, WW Thoburn); CAS-SU 11488 (Monterey, WW Thoburn).

***Sebastescrameri*** (Jordan, 1897). **Darkblotched Rockfish**. Museum specimens: CAS-ICH 37537 (west of Point Sur, WN Eschmeyer); CAS-ICH 47405 (Monterey Bay, WA Laroche and S Moreland); MLMLF0661 (Monterey Bay, ME Anderson).

***Sebastesdallii*** (Eigenmann & Beeson, 1894). **Calico Rockfish**. Described from Monterey; obtained at San Francisco market. Museum specimens: Lectotype [CAS-SU 3893 (drawn specimen), Lectotype established (as figured specimen) in Jordan 1896]; CAS-ICH 217649 (SSE of Point Santa Cruz at Monterey Bay, RN Lea). Publications: Eigenmann and Beeson (1894) (original description); Jordan (1896) (lectotype established); Cailliet et al. (1977) (Dairy at Elkhorn Slough).

***Sebastesdiaconus*** Frable, Wagman, Frierson, Aguilar, and Sidlauskas, 2015. **Deacon Rockfish**. Museum specimens: CAS-SU 11776 (Monterey, DS Jordan); CAS-SU 15112 (Pacific Grove, C Hubbs); UW 40703 (Monterey Bay, WI Follett). Publication: Frable et al. (2015) (new species description, non-type material examined from MBNMS, including Monterey and Pacific Grove listed above). Common name follows Frable et al. (2015).

***Sebastesdiploproa*** (Gilbert, 1890). **Splitnose Rockfish**. Museum specimens: CAS-ICH 37538 (west of Point Sur, K Hakanson); CAS-ICH 39846 (off Point Pinos, PA Gregory); CAS-SU 39916 (Monterey Bay, WA Laroche).

***Sebasteselongatus*** Ayres, 1859. **Greenstriped Rockfish**. Museum specimens: CAS-ICH 51203 (Ascension Canyon shelf and slope, S Moreland); CAS-SU 11490 (Monterey, WW Thoburn); CAS-SU 11774 (Monterey, DS Jordan).

***Sebastesemphaeus*** (Starks, 1911). **Puget Sound Rockfish**. Museum specimens: Monterey Bay Aquarium live specimens (Point Pinos, J Welsh and RN Lea). Two live specimens collected west of Point Pinos (Nov 2014, 225 feet; and Nov 2015, 220 feet) and kept alive on display at Monterey Bay Aquarium (pers. comm. Joe Welsh, Monterey Bay Aquarium).

***Sebastesensifer*** Chen, 1971. **Swordspine Rockfish**. Museum specimens: Paratype (SIO 55-97, Monterey); CAS-ICH 42548 (Monterey Canyon, RN Lea); MLMLF0671 (Carmel Bay, LL Smith).

***Sebastesentomelas*** (Jordan & Gilbert, 1880). **Widow Rockfish**. Described from deep water outside Monterey Bay. Museum specimens: Syntypes [USNM 27044 (orig. 3, now 2)]; CAS-ICH 14865 (off Half Moon Bay, L Hallacher); CAS-ICH 56949 (off Lovers Point, RN Lea); CAS-SU 15102 (Pacific Grove, C Hubbs). Publication: Jordan and Gilbert (1880f) (original description).

***Sebasteseos*** (Eigenmann & Eigenmann, 1890). **Pink Rockfish**. Museum specimens: CAS-ICH 39847 (off Point Pinos, PA Gregory); MLMLF0673 (Monterey Bay, ME Anderson); SIO 07-183 (Ascension Canyon, RN Lea).

***Sebastesflavidus*** (Ayres, 1862). **Yellowtail Rockfish**. Museum specimens: CAS-ICH 26761 (north of Bird Rock at Asilomar, WI Follett); CAS-SU 4125 (Monterey, DS Jordan); CAS-SU 11471 (Monterey, CH Gilbert).

***Sebastesgilli*** (Eigenmann, 1891). **Bronzespotted Rockfish**. Publication: Phillips (1957) (Monterey).

***Sebastesgoodei*** (Eigenmann & Eigenmann, 1890). **Chilipepper**. Museum specimens: CAS-ICH 27358 (off Santa Cruz, RL Bolin); CAS-ICH 37521 (west of Point Sur, WN Eschmeyer); CAS-ICH 40331 (SSW of Point Piedras Blancas, WC Ruark).

***Sebasteshelvomaculatus*** Ayres, 1859. **Rosethorn Rockfish**. Museum specimens: CAS-ICH 42549 (west edge of Monterey Canyon, RN Lea); SIO 00-83 (Bixby Bridge, Monterey County, RN Lea); SIO 98-116 (Carmel Bay, RN Lea).

***Sebasteshopkinsi*** (Cramer, 1895). **Squarespot Rockfish**. Described from Pacific Grove. Museum specimens: Lectotype (CAS-SU 3232, Lectotype established in Jordan 1896); Paralectotypes [CAS-SU 2282 (3, Pacific Grove); CAS-SU 3112 (Monterey Bay]; CAS-SU 4146 (Monterey, CH Gilbert). Publications: Cramer (1895) (original description); Jordan (1896) (lectotype established).

***Sebastesjordani*** (Gilbert, 1896). **Shortbelly Rockfish**. Museum specimens: CAS-ICH 25879 (Monterey Bay, WI Follett); CAS-ICH 37522 (west of Point Sur, KR Hakanson); CAS-ICH 37504 (west of Pigeon Point, T Iwamoto).

***Sebasteslevis*** (Eigenmann & Eigenmann, 1889). **Cowcod**. Museum specimens: CAS-ICH 37503 (west of Pigeon Point, WN Eschmeyer); CAS-ICH 79580 (Carmel Bay, D Ventresca, Hausehildt, and J Guererro); CAS-SU 11492 (Monterey, CH Gilbert); SWFSC uncatalogued (Davidson Seamount during May 2015, W Watson).

***Sebastesmacdonaldi*** (Eigenmann & Beeson, 1893). **Mexican Rockfish**. Museum specimen: CAS-ICH 217644 (Santa Cruz, Sept 2002, identifier unknown, CDFG). Publications: Phillips (1961) (off Point Sur during 1959, El Niño); Radovich (1961) (Point Sur during 1959, El Niño). Categorized as occurring during warm-water events (e.g., El Niño).

***Sebastesmaliger*** (Jordan & Gilbert, 1880). **Quillback Rockfish**. Museum specimens: CAS-ICH 39857 (north of Malpaso Creek, RN Lea); CAS-ICH 51181 (off Davenport, S Moreland); CAS-SU 11497 (Monterey, WW Thoburn).

***Sebastesmelanops*** Girard, 1856. **Black Rockfish**. Museum specimens: CAS-ICH 25885 (Pacific Grove, WI Follett); CAS-ICH 42368 (Soberanes Point tidepool, WA Laroche); CAS-SU 15133 (Del Monte Beach, C Hubbs). Publication: Cailliet et al. (1977) (Dairy at Elkhorn Slough).

***Sebastesmelanostictus*** (Matsubara, 1934). **Blackspotted Rockfish**. Publication: Orr and Hawkins (2008). Authors provide evidence to resurrect the species; distinguishing it from the more northern *S.aleutianus* (Rougheye Rockfish). *Sebastesmelanostictus* (Blackspotted Rockfish) ranges from central Japan, through Aleutian Islands and Bering Sea, to southern California, and collected at depths from 84 to at least 490 m. No museum specimens known from MBNMS. Because of previous confusion with species of the Rougheye Rockfish complex, museum specimens may be incorrectly identified. Because of the recent species description and geographic range of examined specimens (to the north and south of MBNMS, Orr and Hawkins 2008), we include the species on the list.

***Sebastesmelanostomus*** (Eigenmann & Eigenmann, 1890). **Blackgill Rockfish**. Museum specimens: CAS-ICH 37534 (off Yankee Point, WN Eschmeyer); CAS-ICH 37539 (west of Sur Ridge, WN Eschmeyer); SIO 00-46 (Carmel Bay, RN Lea).

***Sebastesminiatus*** (Jordan & Gilbert, 1880). **Vermilion Rockfish**. Described from Monterey and Santa Barbara. Museum specimens: Syntypes [several specimens including: USNM 26965 (orig. 17, now 3), ZMB 11710 (1)]; CAS-ICH 79586 (off Piedras Blancas, RN Lea); CAS-SU 5552 (Pacific Grove, EC Starks). Publication: Jordan and Gilbert (1880b) (original description).

***Sebastesmystinus*** (Jordan & Gilbert, 1881). **Blue Rockfish**. Museum specimens: Paralectotype [off Monterey, USNM 26971]; CAS-ICH 14806 (Monterey Bay, DW Behrens); CAS-ICH 25884 (Pacific Grove, WI Follett); LACM 50157.002 (north of San Simeon, BW Walker and class). Publications: Jordan and Gilbert (1881) (original description); Yoklavich et al. (1991) (Hwy 1 Bridge and Dairy at Elkhorn Slough); Frable et al. (2015) (re-described species, and established lectotype from San Francisco).

***Sebastesnebulosus*** Ayres, 1854. **China Rockfish**. Museum specimens: SIO 65-355 (1 mile off Point Joe, HG Moser); CAS-SU 4149 (Monterey, DS Jordan); CAS-SU 11549 (Monterey, WW Thoburn).

***Sebastesnigrocinctus*** Ayres, 1859. **Tiger Rockfish**. Museum specimen: SIO 91-113 (Monterey Bay, N Lai and J Butler).

***Sebastesovalis*** (Ayres, 1862). **Speckled Rockfish**. Museum specimens: CAS-ICH 47398 (off Point Piedras Blancas, RN Lea); CAS-ICH 54857 (off Carmel Bay, RN Lea); CAS-SU 4429 (Monterey, DS Jordan).

***Sebastespaucispinis*** Ayres, 1854. **Bocaccio**. Museum specimens: CAS-ICH 25878 (Monterey Bay, WI Follett); CAS-ICH 40639 (SW of Soberanes Point, WA Laroche); CAS-ICH 234248 (Elkhorn Slough, DW Behrens); SWFSC uncatalogued (Davidson Seamount during May 2015, W Watson). Publication: Yoklavich et al. (1991) (Hwy 1 Bridge and Dairy at Elkhorn Slough).

***Sebastesphillipsi*** (Fitch, 1964). **Chameleon Rockfish**. Publications: Phillips (1957) (“Hybrid A,” described in Fitch 1964, Monterey during 1939); Fitch (1964) (Monterey during 1939).

***Sebastespinniger*** (Gill, 1864). **Canary Rockfish**. Museum specimens: CAS-SU 10007 (Pacific Grove, WW Thoburn); CAS-SU 10015 (Monterey, WW Thoburn); CAS-SU 11469 (Monterey, CH Gilbert).

***Sebastesproriger*** (Jordan & Gilbert, 1880). **Redstripe Rockfish**. Described from Monterey Bay and Farallones. Museum specimens: Syntypes [USNM 26980 (3), USNM 27105 (4)]; CAS-ICH 66759 (off Yankee Point, RN Lea); CAS-ICH 217645 (off Point Sur, identifier unknown). Publication: Jordan and Gilbert (1880l) (original description).

***Sebastesrastrelliger*** (Jordan & Gilbert, 1880). **Grass Rockfish**. Museum specimens: CAS-ICH 40642 (Elkhorn Slough, WA Laroche); CAS-SU 11494 (Monterey, WW Thoburn); CAS-SU 15138 (Elkhorn Slough, C Hubbs). Publication: Cailliet et al. (1977) (Hwy 1 Bridge, Dairy, and Kirby Park at Elkhorn Slough).

***Sebastesrosaceus*** Girard, 1854. **Rosy Rockfish**. Museum specimens: CAS-ICH 33507 (kelp bed off Carmel River, L Hallcher); CAS-ICH 66327 (north of Bird Rock at Asilomar, WI Follett); CAS-SU 4154 (Monterey, DS Jordan).

***Sebastesrosenblatti*** Chen, 1971. **Greenblotched Rockfish**. Museum specimens: CAS-ICH 39848 (off Point Pinos, PA Gregory); MLMLF0772 (Monterey Bay, ME Anderson); SIO 09-194 (Monterey Canyon, RN Lea).

***Sebastesruberrimus*** (Cramer, 1895). **Yelloweye Rockfish**. Museum specimens: SIO 01-111 (Partington Canyon, RN Lea); SIO 09-194 (Montery Canyon, P Reilly); CAS-SU 4380 (Monterey, DS Jordan).

***Sebastesrubrivinctus*** (Jordan & Gilbert, 1880). **Flag Rockfish**. Described from Monterey and Santa Barbara. Museum specimens: Syntypes [USNM 26989 (5)]; MLMLF0776 (off Davenport, D Varoujean); MLMLF0777 (off Moss Landing, GM Cailliet). Publication: Jordan and Gilbert (1880i) (original description).

***Sebastesrufus*** (Eigenmann & Eigenmann, 1890). **Bank Rockfish**. Museum specimens: CAS-ICH 47379 (west of Point Sur, RN Lea); CAS-ICH 47394 (WNW of Pigeon Point, RN Lea); CAS-ICH 51854 (off Davenport, RN Lea).

***Sebastessaxicola*** (Gilbert, 1890). **Stripetail Rockfish**. Museum specimens: CAS-ICH 37481 (off Santa Cruz, T Iwamoto); CAS-ICH 51855 (off Monterey Coast Guard Pier, RN Lea); CAS-ICH 56273 (off Soberanes Point, W Laroche).

***Sebastessemicinctus*** (Gilbert, 1897). **Halfbanded Rockfish**. Museum specimens: CAS-ICH 42551 (Monterey Bay, RN Lea); SIO 85-154 (off Half Moon Bay, D Gibson); SIO 85-155 (Pigeon Point, D Gibson).

***Sebastesserranoides*** (Eigenmann & Eigenmann, 1890). **Olive Rockfish**. Museum specimens: CAS-ICH 40657 (Elkhorn Slough, WA Laroche); CAS-ICH 42366 (Half Moon Bay, WA Laroche); CAS-ICH 56954 (Carmel Bay, RN Lea); CAS-SU 15135 (Del Monte Beach, C Hubbs).

***Sebastesserriceps*** (Jordan & Gilbert, 1880). **Treefish**. Museum specimens: CAS-ICH 79585 (off Point Joe during 1980, identifier unknown); CAS-SU 11767 (Monterey during Dec 1895, DS Jordan); USNM 110781 (Monterey, DS Jordan).

***Sebastessimulator*** Chen, 1971. **Pinkrose Rockfish**. Museum specimens: SIO 00-58 (Carmel Bay, RN Lea); SIO 85-73 (Point Sur, W Wakefield); SIO 96-71 (Monterey Bay, S Melton).

***Sebastesumbrosus*** (Jordan & Gilbert, 1882). **Honeycomb Rockfish**. Museum specimen: CAS-ICH 14990 (Point Pinos during Sept 1971, RN Lea).

***Sebasteswilsoni*** (Gilbert, 1915). **Pygmy Rockfish**. Described from Monterey Bay. Museum specimens: Holotype (USNM 75811); Paratype (CAS-SU 22992); CAS-ICH 15052 (SW of Santa Cruz, JE Fitch); CAS-ICH 47386 (off Point Sur, RN Lea). Publication: Gilbert (1915) (original description).

***Sebasteszacentrus*** (Gilbert, 1890). **Sharpchin Rockfish**. Museum specimens: CAS-ICH 50020 (NW of Point Piedras Blancas, RN Lea); SIO 09-197 (Soquel Canyon, identifier unknown); SIO 96-71 (Monterey Bay, S Melton).

***Sebastolobusalascanus*** Bean, 1890. **Shortspine Thornyhead**. Museum specimens: CAS-ICH 37536 (west of Yankee Point, WN Eschmeyer); CAS-ICH 236649 (off Monterey Bay, RL Bolin); CAS-SU 65811 (Monterey Canyon, TN Fast). Publication: Burton and Lundsten (2008) (Davidson Seamount, based on imagery). Visual record: MBARI/NOAA T428-03 (Davidson Seamount, EJ Burton, GM Cailliet, T Trejo).

***Sebastolobusaltivelis*** Gilbert, 1896. **Longspine Thornyhead**. Museum specimens: SIO 67-102 (within Davidson Seamount Management Zone, CL Hubbs); CAS-SU 5270 (SW of Santa Cruz, identifier unknown, US Fish Commission); CAS-SU 63869 (off Carmel, RL Bolin); CAS-SU 65816 (Monterey Canyon, TN Fast).

##### Family TRIGLIDAE – searobins

***Prionotusstephanophrys*** Lockington, 1881. **Lumptail Searobin**. Museum specimens: KU 23710 (Monterey Bay during 1993, El Niño, M Domeier); MLMLF0818 (Monterey Bay during Aug 1999, B Banks). Publication: Lea and Rosenblatt (2000) (Monterey Bay during Oct 1998 and Aug 1999, El Niño). Categorized as occurring during warm-water events (e.g., El Niño).

##### Family ANOPLOPOMATIDAE – sablefishes

***Anoplopomafimbria*** (Pallas, 1814). **Sablefish**. Museum specimens: SIO 05-89 (off Lucia, D Kamikawa); SIO 67-108 (SW of Santa Cruz, CL Hubbs); SIO 85-154 (off Half Moon Bay, D Gibson); SIO 91-113 (Monterey Bay, N Lai and J Butler). Publication: Cox (1948) (Monterey).

***Erilepiszonifer*** (Lockington, 1880). **Skilfish**. Described from Monterey. Museum specimens: Holotype (USNM 27111); CAS-ICH 27079 (SW of Point Santa Cruz, JB Phillips); CAS-ICH 39596 (SSW of Point Santa Cruz, DJ Miller and RN Lea). Publications: Lockington (1880) (original description); Phillips (1966) (SW of Point Santa Cruz); Phillips (1967) (west of Moss Landing).

##### Family HEXAGRAMMIDAE – greenlings

***Hexagrammosdecagrammus*** (Pallas, 1810). **Kelp Greenling**. Museum specimens: MLMLF0830 (0.25 miles east of Hwy 1 Bridge at Moss Landing, B Antrim); CAS-SU 4147 (Monterey, DS Jordan); CAS-SU 58375 (off Yankee Point, JC Briggs).

***Hexagrammoslagocephalus*** (Pallas, 1810). **Rock Greenling**. Museum specimens: CAS-ICH 21233 (Moss Beach, J Quast); LACM 3109 (N San Simeon, B Walker); UMMZ 63675 (Point Joe, CL and LC Hubbs).

***Ophiodonelongatus*** Girard, 1854. **Lingcod**. Museum specimens: MLMLF0836 (north of Sandholdt Road Bridge at Moss Landing, LT Ackerman); SIO 62-269 (off Pigeon Point, EA Best); SIO 67-108 (Monterey Bay, CL Hubbs); SIO 84-46 (off Point Sur, W Wakefield). Publication: Yoklavich et al. (1991) (Hwy 1 Bridge and Dairy at Elkhorn Slough).

***Oxylebiuspictus*** Gill, 1862. **Painted Greenling**. Museum specimens: CAS-ICH 19581 (Soberanes Point tidepool, RR Harry); CAS-ICH 21234 (Monterey, WI Follett); CAS-ICH 39456 (Monastery Beach, L Hallacher).

***Pleurogrammusmonopterygius*** (Pallas, 1810). **Atka Mackerel**. Museum specimens: CAS-ICH 25733 (off Santa Cruz, identifier unknown); CAS-SU 47173 (off Monterey, RL Bolin); Publication: Bolin (1952) (outer coast of Monterey Peninsula).

***Zaniolepisfrenata*** Eigenmann and Eigenmann, 1889. **Shortspine Combfish**. Museum specimens: CAS-ICH 40333 (SSW of Point Piedras Blancas, WC Ruark); CAS-ICH 46371 (Monterey Bay, M Hearne); USNM 148501.5223298 (west of Cambria, identifier unknown, “Albatross” Expedition).

***Zaniolepislatipinnis*** Girard, 1858. **Longspine Combfish**. Museum specimens: CAS-ICH 12497 (off Point Montara, EC Johnston); CAS-ICH 42561 (Monterey Bay, RN Lea); CAS-SU 16001 (Monterey Bay, RL Bolin).

##### Family RHAMPHOCOTTIDAE – grunt sculpins

***Rhamphocottusrichardsonii*** Günther, 1874. **Grunt Sculpin**. Museum specimens: MLMLF0854 (Cannery Row, ME Anderson); SIO 00-49 (off Point San Simeon, RN Lea); SIO 01-79 (off Point Sur, RN Lea).

##### Family COTTIDAE – sculpins

***Artediuscorallinus*** (Hubbs, 1926). **Coralline Sculpin**. Museum specimens: CAS-ICH 39853 (off Lovers Point, RN Lea); CAS-ICH 51861 (south of Point Sur, RN Lea); CAS-SU 35364 (Point Lobos, RL Bolin).

***Artediusfenestralis*** Jordan and Gilbert, 1883. **Padded Sculpin**. Museum specimens: CAS-ICH 21726 and CAS-ICH 21914 (off Alameda, San Francisco Bay, JD Hopkirk); SIO 07-185 (near Alcatraz, San Francisco Bay, RN Lea). Other records occur to the north of MBNMS, including CAS-ICH 27315 (Duxbury Point, HB Dietrich). Specimens south of San Francisco Bay were re-examined. Yankee Point specimen (KU 12802) re-determined as *Clinocottusanalis* by William Leo Smith (University of Kansas, 26 Oct 2017). Diablo Canyon specimens (LACM 31700-004) re-determined as *Artediusharringtoni* (orbital cirrus present) by Rick Feeney (Natural History Museum Los Angeles County, Oct 2017). Southern-most records are from San Francisco Bay. We include here, because they presumably traversed MBNMS waters from the north.

***Artediusharringtoni*** (Starks, 1896). **Scalyhead Sculpin**. Museum specimens: CAS-ICH 41715 (Natural Bridges State Park, KM Howe); MLMLF0862 (main channel Elkhorn Slough, ME Anderson); CAS-SU 40884 (Point Lobos tidepool, RL Bolin).

***Artediuslateralis*** (Girard, 1854). **Smoothhead Sculpin**. Described from Monterey and San Luis Obispo. Museum specimen: Syntype (location of Monterey specimen unknown); MLMLF0864 and MLMLF0865 (Carmel Point tidepools, E Yarberry); MLMLF0866 (off Davenport, J Gates and R Banek). Publication: Girard (1854) (original description).

***Artediusnotospilotus*** Girard, 1856. **Bonyhead Sculpin**. Museum specimens: CAS-ICH 50403, 50411 (Moss Beach, D Begle); MLMLF0869 (Elkhorn Slough, B Antrim); CAS-SU 3852 (Monterey, RL Bolin).

***Ascelichthysrhodorus*** Jordan and Gilbert, 1880. **Rosylip Sculpin**. Museum specimens: CAS-ICH 17861 (Pillar Point, MG Bradbury); CAS-ICH 20237 (Moss Beach, RR Harry); CAS-SU 49539 (Moss Beach, RF Ford).

***Chitonotuspugetensis*** (Steindachner, 1876). **Roughback Sculpin**. Museum specimens: CAS-ICH 7360 (Monterey Bay, RL Bolin); CAS-ICH 12531 (west of Montara, EC Johnston); CAS-ICH 216330 (west of Santa Cruz, JD Hopkirk); MLMLF0881 (Kirby Park at Elkhorn Slough, ME Anderson).

***Clinocottusacuticeps*** (Gilbert, 1986). **Sharpnose Sculpin**. Museum specimens: CAS-ICH 216269 (Moss Beach, RR Harry); CAS-ICH 225290 (Half Moon Bay, DA Neely); LACM 35299.003 (Half Moon Bay, C Swift and K Howe).

***Clinocottusanalis*** (Girard, 1858). **Wooly Sculpin**. Described from Monterey. Museum specimens: Syntypes (FMNH 210, USNM 486); CAS-ICH 7371 (Point Lobos, RL Bolin); CAS-ICH 11964 (Moss Beach, RR Harry); CAS-ICH 32984 (north of San Simeon Beach, WN Eschmeyer and SG Poss). Publication: Girard (1858b) (original description).

***Clinocottusembryum*** (Jordan & Starks, 1895). **Calico Sculpin**. Museum specimens: CAS-ICH 78466 (Pigeon Point, D Catania); CAS-SU 35394 (Point Lobos, RL Bolin); CAS-SU 58467 (south of Yankee Point, JC Briggs).

***Clinocottusglobiceps*** (Girard, 1858). **Mosshead Sculpin**. Museum specimens: CAS-ICH 25280 (Moss Beach, WI Follett and L Dempster); CAS-ICH 216419 (Soberanes Point, RL Bolin); CAS-SU 58415 (south of Yankee Point, JC Briggs).

***Clinocottusrecalvus*** (Greeley, 1899). **Bald Sculpin**. Described from Pacific Grove. Museum specimens: Holotype (CAS-SU 6068); CAS-ICH 25279 (Moss Beach, WI Follett and L Dempster); CAS-ICH 216412 (Soberanes Point, AO Flechsig); CAS-SU 16013 (Point Lobos, RL Bolin). Publication: Greeley (1899) (original description).

***Enophrysbison*** (Girard, 1854). **Buffalo Sculpin**. Museum specimens: CAS-ICH 37545 (Moss Beach, RF Ford); MLMLF0903 (Hwy 1 Bridge at Elkhorn Slough, ME Anderson); CAS-SU 35329 (Del Monte Beach pier, RL Bolin).

***Enophrystaurina*** Gilbert, 1914. **Bull Sculpin**. Described from Monterey Bay, near Pacific Grove. Museum specimens: Holotype (USNM 75064); CAS-ICH 66760 (off Otter Point to Lovers Point, RN Lea); CAS-ICH 232701 (Monterey Bay, TN Fast). Publication: Gilbert (1914) (original description).

***Hemilepidotushemilepidotus*** (Tilesius, 1811). **Red Irish Lord**. Museum specimens: CAS-ICH 35284 (Mussel Point, RL Bolin); SIO 72-86 (north of Point San Simeon, R Rosenblatt); UMMZ 63379 (Carmel Bay, CL Hubbs and LC Hubbs).

***Hemilepidotusspinosus*** Ayres, 1854. **Brown Irish Lord**. Museum specimens: CAS-ICH 18855 (Moss Beach, RR Harry); CAS-ICH 28471 (Carmel Bay, RN Lea); CAS-ICH 36410 (Monterey, JE McCosker).

***Icelinusburchami*** Evermann and Goldsborough, 1907. **Dusky Sculpin**. Museum specimens: CAS-ICH 234831 (off Point Sur, RL Bolin and PL Budd); SIO 01-117 (Carmel Canyon, P Reilly); SIO 99-80 (Monterey Bay, P Reilly and J Spratt).

***Icelinuscavifrons*** Gilbert, 1890. **Pit-head Sculpin**. Museum specimens: LACM 52245.014 (off of San Simeon Point, E Hobson et al.); LACM 52277.008 and CAS-SU 64410 (Monterey Bay, TN Fast).

***Icelinusfilamentosus*** Gilbert, 1890. **Threadfin Sculpin**. Museum specimens: CAS-ICH 37505 (off Davenport, WN Eschmeyer); CAS-ICH 37518 (west of Point Sur, KR Hakanson); CAS-ICH 42547 (off Cypress Point, RN Lea); CAS-SU 23070 (Point Lobos, RL Bolin).

***Icelinusfimbriatus*** Gilbert, 1890. **Fringed Sculpin**. Museum specimens: CAS-ICH 16145 (off Point Pinos, identifier unknown); CAS-SU 40922 (Monterey Bay, A Peden); CAS-SU 58419 (Monterey Bay, JC Briggs).

***Icelinusoculatus*** Gilbert, 1890. **Frogmouth Sculpin**. Museum specimens: SIO 97-126 (off Cypress Point, J Field); SIO 99-41 (off Point Sur, RN Lea); SIO 99-81 (Monterey Bay, RN Lea).

***Icelinusquadriseriatus*** Lockington, 1880. **Yellowchin Sculpin**. Museum specimens: CAS-ICH 7364 (Monterey Bay, RL Bolin); CAS-ICH 12780 (off Point Montara, identifier unknown, US Fish Commission); CAS-SU 40925 (off Santa Cruz, RL Bolin).

***Icelinustenuis*** Gilbert, 1890. **Spotfin Sculpin**. Museum specimens: CAS-ICH 37520 (west of Point Sur, KR Hakanson); CAS-SU 21348 (Monterey Bay, RL Bolin); CAS-SU 58456 (Monterey Bay, JC Briggs).

***Jordaniazonope*** Starks, 1895. **Longfin Sculpin**. Museum specimens: LACM 52248.018 (Jade Cove, E Hobson); SIO 72-86 (north of Point San Simeon, R Rosenblatt); CAS-SU 35331 (off Asilomar, RL Bolin).

***Leptocottusarmatus*** Girard, 1854. **Pacific Staghorn Sculpin**. Museum specimens: CAS-ICH 48200 (Monterey Bay, S Richardson); CAS-SU 15028 (upper Elkhorn Slough, CL Hubbs); CAS-SU 47497 (Elkhorn Slough, WC Freihofer). Publication: Yoklavich et al. (1991) (Elkhorn Slough).

***Oligocottusmaculosus*** Girard, 1856. **Tidepool Sculpin**. Museum specimens: CAS-ICH 18832 (Moss Beach: RR Harry); CAS-ICH 25276 (Moss Beach, WI Follett and L Dempster); CAS-SU 4244 (Pacific Grove, identifier unknown).

***Oligocottusrimensis*** (Greeley, 1899). **Saddleback Sculpin**. Described from Point Lobos. Museum specimens: Holotype (CAS-SU 6067); CAS-ICH 47384 (Carmel kelp bed, RN Lea); CAS-ICH 48465 (Pigeon Point tidepools, D Catania); CAS-ICH 212726 (Soberanes Point tidepool, RR Harry). Publication: Greeley (1899) (original description).

***Oligocottusrubellio*** (Greeley, 1899). **Rosy Sculpin**. Described from Monterey Bay. Museum specimens: Holotype (CAS-SU 6066); CAS-ICH 212628 (Soberanes Point, S Weitzman); CAS-SU 35342 (Point Lobos, RL Bolin); CAS-SU 40944 (Pescadero Point, RL Bolin); CAS-SU 48926 (north of San Simeon, M Bradbury). Publication: Greeley (1899) (original description).

***Oligocottussnyderi*** Greeley, 1898. **Fluffy Sculpin**. Described from Pacific Grove. Museum specimens: Lectotype (CAS-SU 5846, Lectotype apparently established by Greeley 1899); Paralectotypes [CAS-SU 5847 (5)]; CAS-ICH 7361 (Point Lobos, RL Bolin); CAS-ICH 18628 (Moss Beach, RR Harry); CAS-ICH 27637 (Natural Bridges State Park, B Wesemann). Publications: Greeley in Jordan and Evermann (1898) (type specimen); Greeley (1899) (lectotype established).

***Orthonopiastriacis*** Starks and Mann, 1911. **Snubnose Sculpin**. Museum specimens: CAS-ICH 15793 (Carmel Bay, RN Lea); CAS-ICH 51862 (south of Point Sur, RN Lea); CAS-ICH 56947 (off Lovers Point, RN Lea). Publication: Gilbert (1914) (Monterey Bay).

***Radulinusasprellus*** Gilbert, 1890. **Slim Sculpin**. Museum specimens: CAS-ICH 37496 (off Half Moon Bay, T Iwamoto); SIO 60-430 (south of San Simeon Point, W Dahlstrom); SIO 98-113 (Monterey Bay, RN Lea).

***Radulinusboleoides*** Gilbert, 1898. **Darter Sculpin**. Museum specimen: CAS-ICH 40889 (SW of Soberanes Point, RN Lea); MLMLF0951 (Soberanes Point, B Antrim).

***Ruscariuscreaseri*** (Hubbs, 1926). **Roughcheek Sculpin**. Museum specimens: CAS-ICH 30460 (Monterey Breakwater, ME Anderson); LACM 52248.005 (Jade Cove, ES Hobson); SIO 72-86 (off San Simeon Point, R Rosenblatt).

***Scorpaenichthysmarmoratus*** (Ayres, 1854). **Cabezon**. Museum specimens: CAS-ICH 7365 (Elkhorn Slough, Bolin); CAS-ICH 11037 (Moss Beach, BW Halstead); CAS-ICH 32990 (north of San Simeon Beach State Park, WN Eschmeyer and SG Poss). Publication: Yoklavich et al. (1991) (Hwy 1 Bridge, Dairy, and Kirby Park at Elkhorn Slough).

***Synchirusgilli*** Bean, 1890. **Manacled Sculpin**. Museum specimens: CAS-ICH 47391 (Carmel kelp bed, RN Lea); CAS-ICH 216550 (Carmel Bay kelp canopy, ME Anderson); CAS-SU 15521 (Monterey, RL Bolin).

***Zesticelusprofundorum*** (Gilbert, 1896). **Flabby Sculpin**. Museum specimens: MLMLF0967 (Monterey Bay, identifier unknown); SIO 85-69 (off Point Sur, W Wakefield); CAS-SU 25277 (off Point Pinos, RL Bolin, “Albatross” collection). Publication: Gilbert (1915) (Monterey, “Albatross” collection).

##### Family HEMITRIPTERIDAE – searavens

***Blepsiascirrhosus*** (Pallas, 1814). **Silverspotted Sculpin**. Museum specimens: LACM 47917.001 (San Simeon, BW Walker, AO Flechsig, AB Rechnitzer); SIO 50-193A (San Simeon Bay, F Taylor and AA Allanson); SIO 73-220 (Point Piedras Blancas, R Rosenblatt).

***Nautichthysoculofasciatus*** (Girard, 1858). **Sailfin Sculpin**. Museum specimens: CAS-ICH 25890 and CAS-ICH 31066 (Pacific Grove, WI Follett); LACM 7939 (off San Simeon, B Walker); LACM 52248.025 (Jade Cove, ES Hobson).

##### Family AGONIDAE – poachers

***Agonomalusmozinoi*** Wilimovsky and Wilson, 1979. **Kelp Poacher**. Museum specimens: Paratype (CAS-ICH 40716, Monastery Beach); CAS-ICH 64652 (off Cannery Row, Monterey, ME Anderson); SIO 09-291 (Carmel Bay, RN Lea). Publication: Wilimovsky and Wilson (1979) (original description). Recognized by some researchers as *Hypsagonusmozinoi*. We follow Sheiko and Mecklenburg (2004), and current usage in "Catalog of Fishes" (Eschmeyer et al. 2017), with placement in *Agonomalus*.

***Agonopsissterletus*** (Gilbert, 1898). **Southern Spearnose Poacher**. Museum specimen: UCLA W 66-67 (San Simeon Point, identifier unknown; specimen pending accession at SIO, out on loan to Russia at time of manuscript preparation). Another specimen (SIO 94-123) collected from nearby Morro Bay; therefore, locality is probable.

***Agonopsisvulsa*** (Jordan & Gilbert, 1880). **Northern Spearnose Poacher**. Museum specimens: MLMLF0975 (off Waddell Creek, GE Kukowski); MLMLF0976 (Monterey Bay, D Varoujean); CAS-SU 63622 (Monterey Bay, ME Anderson).

***Bathyagonuspentacanthus*** (Gilbert, 1890). **Bigeye Poacher**. Museum specimens: SIO 91-115 (Monterey Bay, N Lai and J Butler); CAS-SU 16708 (off Point Sur, H Freeman); CAS-SU 64401 (off Pescadero Point, RL Bolin).

***Bothragonusswanii*** (Steindachner, 1876). **Rockhead**. Museum specimens: CAS-ICH 16152 (Pacific Grove, L Dempster); CAS-ICH 23946 (Carmel Bay, MG Bradbury); LACM 52245.006 (NW of San Simeon Point, E Hobson).

***Chesnoniaverrucosa*** (Lockington, 1880). **Warty Poacher**. Museum specimens: UCLA W 59-135 (4.5 miles west of Point Montara, identifier unknown; specimen pending accession at SIO, pers. comm. HJ Walker, Oct 2017); USNM 48731.5269927 (off Muir Beach, identifier unknown, “Albatross” Expedition).

***Odontopyxistrispinosa*** Lockington, 1880. **Pygmy Poacher**. Museum specimens: CAS-ICH 12347 (off Point Montara Light, EC Johnston); CAS-ICH 18651 (Pacific Grove, RR Harry); SIO 50-193A (San Simeon Bay, F Taylor and AA Allanson, SIO).

***Stellerinaxyosterna*** (Jordan & Gilbert, 1880). **Pricklebreast Poacher**. Described from Santa Cruz beach at Monterey Bay. Museum specimens: Holotype [USNM (not found)]; other material [USNM 27173 and USNM 27395 (Soquel during 1880, DS Jordan)]; CAS-ICH 216557 (San Simeon Bay, AO Flechsig); MLMLF0995 (off Pajaro River at Monterey Bay, GE Kukowski). Publication: Jordan and Gilbert (1880g) (original description).

***Xeneretmuslatifrons*** (Gilbert, 1890). **Blacktip Poacher**. Museum specimens: CAS-ICH 37497 (SSW of Half Moon Bay, T Iwamoto); LACM 30233.002 (off Cypress Point, W Dahlstrom); CAS-SU 3650 (west of Santa Cruz, H Freeman).

***Xeneretmusleiops*** Gilbert, 1915. **Smootheye Poacher**. Museum specimens: SIO 19-119 (Monterey Bay, RN Lea); SIO 97-122 (Carmel Bay, J Spratt and K Schlining); SIO 99-41 (off Point Sur, P Reilly, J Spratt and RN Lea).

***Xeneretmustriacanthus*** (Gilbert, 1890). **Bluespotted Poacher**. Museum specimens: CAS-ICH 14270 (Monterey Bay, RL Bolin); SIO 60-430 (off San Simeon Point, W Wakefield); CAS-SU 69025 (Santa Cruz, A Peden).

##### Family PSYCHROLUTIDAE – fathead sculpins

***Psychrolutesphrictus*** Stein and Bond, 1878. **Blob Sculpin**. Museum specimens: CAS-ICH 40740 (Santa Cruz, B Antrim); MLMLF0971 (off Santa Cruz, B. Antrim). Publications: Burton and Lundsten (2008), and Lundsten et al. (2009) (Davidson Seamount, based on imagery). Visual records: MBARI/NOAA T951-01 (Davidson Seamount: EJ Burton and L Lundsten).

##### Family LIPARIDAE – snailfishes

***Bathyphasmaovigerum*** Gilbert, 1896. **Abyssal Snailfish**. Publication and Visual record: Stein et al. (2006) (Monterey Canyon). Previously recognized as *Careproctusovigerus*. We follow naming convention of Balushkin (2012), and current usage in *Catalog of Fishes* (Eschmeyer et al. 2017). Common name follows Mecklenburg et al. (2002).

***Careproctusfilamentosus*** Stein, 1978. **snailfish**. Publication and Visual record: Stein et al. 2006 (Monterey Canyon). No official common name.

***Careproctusgilberti*** Burke, 1912. **Smalldisk Snailfish**. Museum specimen and Publication: CAS-ICH 29948 (Monterey Bay, Anderson et al. 1979).

***Careproctuskamikawai*** Orr, 2012. **Arbiter Snailfish**. Described from Monterey Bay. Museum specimen: Holotype [USNM 400885 (ex. UW 150324)]. Visual records: MBARI/NOAA T425-08, MBARI/NOAA T425-09 and MBARI/NOAA T425-10 (Davidson Seamount, L Kuhnz). Publication: Orr (2012) (original description). Common name follows Orr (2012).

***Careproctuslongifilis*** Garman, 1892. **Threadfin Snailfish**. Museum specimen and Publication: MBARI 2000321-HFSS11 (Monterey Canyon, Stein et al. 2006).

***Careproctusmelanurus*** Gilbert, 1892. **Blacktail Snailfish**. Museum specimens: CAS-ICH 55146 (west of Pigeon Point, ME Anderson); CAS-ICH 55399 (off Point Sur, ME Anderson); SIO 97-127 (Carmel Canyon, J Field); SIO 99-81 (Monterey Bay, RN Lea). Publication and Visual record: Stein et al. (2006) (Monterey Canyon).

***Elassodiscuscaudatus*** (Gilbert, 1915). **Humpback Snailfish**. Described from Monterey Bay. Museum specimens: Holotype (USNM 75815); CAS-ICH 29947 (Monterey Bay, E Anderson). Publications: Gilbert (1915) (original description); Anderson et al. (1979) (Monterey Bay, CAS-ICH 29947).

***Liparisflorae*** (Jordan & Starks, 1895). **Tidepool Snailfish**. Museum specimens: CAS-ICH 11649 (Moss Beach, RR Harry); CAS-ICH 19877 (Soberanes Point tidepool, RR Harry); CAS-ICH 47388 (Carmel kelp bed, RN Lea); CAS-ICH 30765 (north of San Simeon, AO Flechsig).

***Liparisfucensis*** Gilbert, 1896. **Slipskin Snailfish**. Museum specimens: CAS-ICH 45980 (Monterey, RL Bolin); LACM 7904 (off San Simeon, B Walker); LACM 52248.02 (Jade Cove, E Hobson).

***Liparismucosus*** Ayres, 1855. **Slimy Snailfish**. Museum specimens: CAS-ICH 15044 (Pigeon Point, RN Lea); CAS-ICH 59630 (Carmel Bay, RN Lea); SIO 67-298 (off Piedras Blancas Lighthouse, D Wilkie and CJ Farwell); SIO 87-99 (Soberanes Point, RN Lea).

***Liparispulchellus*** Ayres, 1855. **Showy Snailfish**. Museum specimen: MLMLF1022 (off Waddell Creek, GE Kukowski); CAS-SU 16021 (Monterey Bay, RL Bolin).

***Lipariscusnanus*** Gilbert, 1915. **Pygmy Snailfish**. Described from Monterey Bay. Museum specimens: Holotype (USNM 75817); Paratypes [CAS-SU 22993 (off Santa Cruz Lighthouse), CAS-SU 229994 (off Point Pinos Lighthouse)]; MLMLF1024 (Monterey Bay, ME Anderson). Publication: Gilbert (1915) (original description).

***Nectoliparispelagicus*** Gilbert and Burke, 1912. **Tadpole Snailfish**. Museum specimens: MBARI V3-13-90 (Monterey Canyon, Stein et al. 2006); CAS-SU 63860 (Monterey Bay, RL Bolin); CAS-SU 63904 (Monterey Bay, TN Fast). Publications: Gilbert (1915) (Monterey Bay); Stein et al. (2006) (Monterey Canyon, MBARI V3-13-90).

***Osteodiscuscascadiae*** Stein, 1978. **Bonydisk Snailfish**. Museum specimen and Publication: MBARI 2001276-HFSS6 (Monterey Canyon, Stein et al. 2006). Common name follows Love et al. (2005).

***Paraliparisalbescens*** Gilbert, 1915. **Phantom Snailfish**. Described from off Point Pinos. Museum specimens: Holotype (USNM 75816); CAS-ICH 29950 (Monterey Bay, ME Anderson); CAS-ICH 35952 (Monterey Canyon, ME Anderson); MLMLF1030 (Monterey Canyon, ME Anderson). Publications: Gilbert (1915) (original description); Anderson et al. (1979) (Monterey Bay/Canyon, CAS-ICH 29950, CAS-ICH 35952, and MLMLF1030).

***Paralipariscephalus*** Gilbert, 1892. **Swellhead Snailfish**. Museum specimens: CAS-SU 5232 (SW of Santa Cruz, identifier unknown, US Fish Commission); CAS-SU 5254 (west of Point Año Nuevo, identifier unknown, US Fish Commission).

***Paraliparisdactylosus*** Gilbert, 1896. **Polydactyl Snailfish**. Described from “off Santa Cruz” (station 3112, west of Point Año Nuevo). Museum specimens: Lectotype (USNM 48616, Lectotype established by Burke 1930); Paralectotypes [CAS-SU 3024 (1, west of Point Año Nuevo), USNM 53032 (1, west of Point Año Nuevo)]; MBARI 2002130-HFSS10 (Soquel Canyon; Stein et al. 2006). Publications: Gilbert (1896) (original description); Burke (1930); Stein et al. (2006) (Soquel Canyon, MBARI 2002130-HFSS10). Common name follows Mecklenburg et al. (2002).

***Paraliparisdeani*** Burke, 1912. **Prickly Snailfish**. Museum specimen: CAS-SU 22963 (Point Pinos, identifier unknown, US Fish Commission). Publication: Gilbert (1915) (Monterey Bay, US Fish Commission).

***Paraliparismento*** Gilbert, 1892. **Bulldog Snailfish**. Museum specimen: CAS-SU 22995 (Point Pinos, identifier unknown, US Fish Commission). Publication: Gilbert (1915) (Monterey Bay, US Fish Commission).

***Paraliparispectoralis*** Stein, 1978. **Pectoral Snailfish**. Museum specimen: SIO 92-92 (off Monterey Bay, R Dotson, NMFS). Common name follows Mecklenburg et al. (2002).

***Paraliparisrosaceus*** Gilbert, 1890. **Rosy Snailfish**. Museum specimens: CAS-ICH 31496 (Monterey Bay, ME Anderson); CAS-ICH 34960 (off Davenport, BS Antrim); MLMLF1034 (Ascension Canyon, ME Anderson).

***Paraliparisulochir*** Gilbert, 1896. **Broadfin Snailfish**. Museum specimen: CAS-SU 23000 (Point Pinos, identifier unknown, US Fish Commission).

***Rhinoliparisattenuatus*** Burke, 1912. **Slim Snailfish**. Museum specimens: CAS-SU 22960 and CAS-SU 22969 (off Point Pinos Lighthouse, identifier unknown, US Fish Commission). Publication: Gilbert (1915) (Monterey Bay, US Fish Commission).

***Rhinoliparisbarbulifer*** Gilbert, 1896. **Longnose Snailfish**. Museum specimens: MBARI 2002130-HFSS8 (Soquel Canyon, Stein et al. 2006); SIO 06-15 (east of Sur Ridge, KA Moots). Publication: Stein et al. (2006) (Soquel Canyon, MBARI 2002130-HFSS8).

#### Order Perciformes

##### Family MORONIDAE – temperate basses

***Moronesaxatilis*** (Walbaum, 1791). **Striped Bass**. Museum specimens: MLMLF1038 (Moss Landing Harbor mouth, LT Ackerman); SIO 54-76 (San Simeon Bay, A Rechnitzer); USNM 84567 (Monterey Bay, identifier unknown, “Albatross” Expedition). Publication: Yoklavich et al. (1991) (Hudson’s Landing at Elkhorn Slough). Introduced from New Jersey into San Francisco Bay (1879 and 1882, Stevens and Kohlhorst 2001).

##### Family POLYPRIONIDAE – wreckfishes

***Stereolepisgigas*** Ayres, 1859. **Giant Sea Bass**. Museum specimens: CAS-ICH 58785 (Monterey Bay during Jul 1986, RN Lea); MLMLF1039 (Monterey Bay during Aug 1978, El Niño, D Bedford). Publications: Phillips (1930b) (off Pacific Grove during 1930, El Niño); Phillips (1954) (off Pacific Grove during 1953). Categorized as occurring during warm-water events (e.g., El Niño).

##### Family EPINEPHELIDAE – groupers

***Hyporthodusniphobles*** (Gilbert & Starks, 1897). **Star-studded Grouper**. Museum specimens: LACM 38417.001 (off Point Piedras Blancas during Sept 1975, D Burge); SIO 95-24 (west of Hurricane Point during Nov 1994, RN Lea). Additional specimen captured west of Bixby Bridge during Oct 1992 and examined by RN Lea; providence unknown. This is a southern species that can move northward during warm-water events. Categorized as occurring during warm-water events (e.g., El Niño).

***Mycteropercaxenarcha*** Jordan, 1888. **Broomtail Grouper**. Museum specimen: CAS-ICH 58469 (Monterey Bay, RN Lea).

##### Family SERRANIDAE – sea basses and groupers

***Paralabraxclathratus*** (Girard, 1854). **Kelp Bass**. Museum specimens: MLMLF1049 (Cannery Row kelp beds during Nov 1973, El Niño, ME Anderson); CAS-SU 5556 (Pacific Grove, EC Starks, date unknown); USNM 110287 (Monterey, DS Jordan, date unknown, skull). Publications: Miller and Gotshall (1965) (Princeton west jetty during Sept 1960, after the warm water period, 1957-1959, small individuals appeared in skiff catch throughout Monterey Bay, larger fish landed from Santa Cruz to Monterey); Smith and Gotshall (1967) (Half Moon Bay during 1959). Categorized as occurring during warm-water events (e.g., El Niño).

***Paralabraxmaculatofasciatus*** (Steindachner, 1868). **Spotted Sand Bass**. Listed with reservation; possibly an historic record and/or occurring during warm-water events (e.g., El Niño). Publications: Boulenger (1895); Miller and Gotshall (1965); Miller and Lea (1972).

Miller and Lea (1972) reported the northern limit at Monterey and noted a record from San Francisco in the late 1800s. The 1800s record references Boulenger (1895, British Museum) who described the distribution of *P.maculatofasciatus* as “Coast of California and Mexico, from San Francisco to Mazatlan.” Boulenger (1895) listed three young specimens from San Francisco. However, no specimen currently exists at the British Natural History Museum (BMNH, Mar 2017) as indicated in 1895 publication. We assume these three young specimens were originally misidentified and subsequently removed from the collection. In addition, Jordan and Evermann (1896a, b) did not recognize the San Francisco record, and considered San Pedro as the northern limit (as did Jordan and Gilbert 1882, and Jordan and Eigenmann 1890).

The northern limit at Monterey, noted by Miller and Lea (1972), was likely in reference to Miller and Gotshall (1965) who suggested a range extension for *P.maculatofasciatus* per a sportfish party boat capture in Monterey during Feb 1963 (El Niño occurred during 1957-1959). The specimen was confirmed by Dan Miller (California Department of Fish and Game biologist), but not accessioned in a museum.

***Paralabraxnebulifer*** (Girard, 1854). **Barred Sand Bass**. Described from Monterey. Museum specimens: Syntypes [USNM 282 (2)]. Publication: Girard (1854) (original description).

##### Family PRIACANTHIDAE – bigeyes

***Pristigenysserrula*** (Gilbert, 1891). **Popeye Catalufa**. Museum specimens: CAS-ICH 52604 (off Point San Pedro, San Mateo County during 1983, El Niño, identifier unknown); CAS-ICH 54924 (off Davenport during 1983, El Niño, W Starnes). Publication: Starnes (1988) (Monterey Bay during 1983, El Niño). Categorized as occurring during warm-water events (e.g., El Niño).

##### Family MALACANTHIDAE – tilefishes

***Caulolatilusprinceps*** (Jenyns, 1840). **Ocean Whitefish**. Publications: Miller and Gotshall (1965); Miller and Lea (1972). Typically occurs in warmer water; however, can move north during El Niño years. During 1957-1961, Miller and Gotshall (1965) reported Ocean Whitefish catches as far north as San Francisco. Miller and Lea (1972) list the species as occurring as far north as Vancouver Island, British Columbia; common in southern California, rare north of Monterey. Categorized as occurring during warm-water events (e.g., El Niño).

##### Family CORYPHAENIDAE – dolphinfishes

***Coryphaenahippurus*** Linnaeus, 1758. **Dolphinfish**. Publication: Monterey County Herald (1997) (off Point Pinos). Fishermen caught two specimens off Point Pinos during 1997 El Niño; RN Lea confirmed catch (Monterey County Herald, 2 Sept 1997). Typically occurs in warmer water; however, can move north during El Niño years. Categorized as occurring during warm-water events (e.g., El Niño).

##### Family ECHENEIDAE – remoras

***Remoraaustralis*** (Bennett, 1840). **Whalesucker**. Museum specimen: CAS-ICH 26766 (off Santa Cruz, identifier unknown).

***Remoraremora*** (Linnaeus, 1758). **Remora**. Museum specimens: CAS-ICH 37868 (Monterey Bay, C Woodhill); SIO 93-197 (Monterey Bay, identifier unknown); UMMZ 64123 (off Santa Cruz, identifier unknown).

##### Family CARANGIDAE – jacks

***Caranxcaballus*** Günther, 1868. **Green Jack**. Museum specimen and Publication: CAS-ICH 54926 (Sea Cliff State Beach, Aptos during 1983, El Niño, Lea and Walker 1995). Categorized as occurring during warm-water events (e.g., El Niño).

***Decapterusmuroadsi*** (Temminck & Schlegel, 1844). **Amberstripe Scad**. Museum specimens: CAS-SU 58626 (Pacific Grove, WF Smith-Vaniz); CAS-SU 68836 (Monterey Bay, JR Rainey). Publication: Frey (1962) (Monterey Bay, CAS-SU 58626).

***Naucratesductor*** (Linnaeus, 1758). **Pilotfish**. Museum specimens: CAS-ICH 54922 (3 miles south of Cape San Martin, RN Lea); CAS-SU 15483 (Mussel Point, Monterey, H Miller).

***Serioladorsalis*** (Gill, 1863). **Yellowtail Jack**. Museum specimen: CAS-ICH 37523 (Monterey during 1976, WN Eschmeyer). Previously recognized as *Seriolalalandi*. We follow naming convention of Martinez-Takeshita et al. (2015), and current usage in *Catalog of Fishes* (Eschmeyer et al. 2017).

***Trachurussymmetricus*** (Ayres, 1855). **Jack Mackerel**. Museum specimens: CAS-ICH 7559 (Santa Cruz, B Halstead); CAS-ICH 66526 (Elkhorn Slough, DT Anderson); CAS-SU 48289 (Monterey Beach at Municipal Wharf, FH Berry); CAS-SU 58448 (Elkhorn Slough, JC Briggs). Publication: Fitch (1949) (Davidson Seamount).

##### Family BRAMIDAE – pomfrets

***Bramajaponica*** Hilgendorf, 1878. **Pacific Pomfret**. Museum specimen: CAS-ICH 39597 (Sea Cliff State Beach, Aptos, RN Lea).

***Pteraclisaesticola*** (Jordan & Snyder, 1901). **Pacific Fanfish**. Museum specimen: CAS-ICH 64200 (Sur Canyon during Aug 1987, RN Lea).

##### Family CARISTIIDAE – manefishes

***Caristiusmacropus*** (Bellotti, 1903). **Bigmouth Manefish**. Museum specimen: SIO 98-117 (Canyon off Davenport during 1998, RN Lea).

##### Family HAEMULIDAE – grunts

***Haemuloncaliforniensis*** (Steindachner, 1876). **Salema**. Museum specimens: UW 3172 (4, San Francisco Bay region during 1932, C. Wade). Publication: Phillips (1936) (southern Monterey Bay, 1935). During Mar 2017, RN Lea examined 4 museum specimens and new x-rays (UW 3172), and confirmed identifications. This is a southern species that can move northward during warm-water events. Categorized as historic and occurring during warm-water events (e.g., El Niño).

##### Family POLYNEMIDAE – threadfins

***Polydactylusapproximans*** (Lay & Bennett, 1839). **Blue Bobo**. Museum specimen: CAS-SU 35305 (Monterey Bay during 1941, warm-water event, RL Bolin). Publications: Follett (1948) (Monterey during Jan 1941, CAS-SU 35305); Lea and Rosenblatt (2000) (references Follett 1948 and museum specimen). Categorized as occurring during warm-water events (e.g., El Niño).

##### Family SCIAENIDAE – drums and croakers

***Atractoscionnobilis*** (Ayres, 1860). **White Seabass**. Publication: Radovich (1961) (Monterey during 1958 and 1959, El Niño). Categorized as occurring during warm-water events (e.g., El Niño).

***Genyonemuslineatus*** (Ayres, 1855). **White Croaker**. Museum specimens: CAS-ICH 19866 (Pacific Grove, DA Simpson); CAS-ICH 66670 (Monterey Bay, MG Bradbury); MLMLF1086 (near Sandholdt Road Bridge at Moss Landing, LT Ackerman). Publication: Phillips (1936) (southern Monterey Bay).

***Menticirrhusundulatus*** (Girard, 1854). **California Corbina**. Listed with reservation; possibly an historic record and/or occurring during warm-water events (e.g., El Niño). Publication: Starks (1919). In a review of the croakers (Sciaenidae) of California, Starks (1919) noted “This fish is rather common on sandy shores of southern California and is known southward into the Gulf of California, while individuals are sometimes taken as far northward as San Francisco.” Stark’s information may be based on CAS-SU 21232, from San Francisco, collected by Charles H. Gilbert. No date is listed, but it was likely taken in the late 1800s to early 1900s based on catalog number. We know of one recent record from north of Point Conception, a 474 mm TL fish caught off Morro Rock in Jan 1986 (CAS-ICH 58470); south of MBNMS. During warm-water events, occurrence of this species needs to be verified off the sandy beaches of central California.

***Seriphuspolitus*** Ayres, 1860. **Queenfish**. Museum specimens: ANSP 11543 (San Francisco before 1880, identifier unknown, US Fish Commission); MLMLF1092 (Kirby Park at Elkhorn Slough, E Yarberry); CAS-SU 4153 (San Francisco Market, DS Jordan). Publications: Ayres (1860b) (San Francisco); Phillips (1932a) (Monterey Bay during 1931, El Niño); Skogsberg (1939) (Monterey Bay); Yoklavich et al. (1991) (Kirby Park and Hudson’s Landing at Elkhorn Slough). Occurs in the coastal waters of the Northeast Pacific (Miller and Lea 1972). We include the San Francisco specimens here, because they presumably traversed MBNMS waters.

##### Family KYPHOSIDAE – sea chubs

***Girellanigricans*** (Ayres, 1860). **Opaleye**. Museum specimens: CAS-ICH 66680 (cove SE of Pigeon Point, identifier unknown, 1963); CAS-ICH 76622 (Mussel Point, Pacific Grove, C Limbaugh, Mead, and Patterson); CAS-SU 12083 (Pacific Grove, EC Starks). Publications: Limbaugh (1955) (Pacific Grove); Norris (1963) (Monterey).

***Hermosillaazurea*** Jenkins and Evermann, 1889. **Zebraperch**. Publication: Phillips (1965a) (Monterey). Visual record: SIMoN (2018) (Monterey Harbor during Apr 2012, C King). Based on molecular phylogenetic analysis, recent publications propose a taxonomic revision of the family Kyphosidae, including a name change from *Hermosillaazurea* to *Kyphosusazureus* (Knudsen and Clements 2013, 2016). We do not adopt the name change here and await further widespread acceptance of this proposed revision.

***Kyphosusvaigiensis*** (Quoy & Gaimard, 1825). **Blue-bronze Chub**. Museum specimen: CAS-ICH 56945 (Monterey Bay, Coast Guard Breakwater during 1984, El Niño, RN Lea, as *Kyphosusanalogus*). Categorized as occurring during warm-water events (e.g., El Niño). Knudsen and Clements (2013) determined *Kyphosusanalogus* (Gill, 1862) a junior synonym of *K.vaigiensis* (Quoy & Gaimard, 1825).

***Medialunacaliforniensis*** (Steindachner, 1876). **Halfmoon**. Museum specimens: CAS-SU 11871 (Monterey Bay during Sept 1896, SM Duarte); CAS-SU 35303 (vicinity of Half Moon Bay during Sept 1941, warm water year, RL Bolin); CAS-SU 35304 (off Point Sur, RL Bolin, date unknown). Publication: Radovich (1961) (Santa Cruz Municipal Wharf during 1958, El Niño). Categorized as occurring during warm-water events (e.g., El Niño).

##### Family PENTACEROTIDAE – armorheads

***Pseudopentaceroswheeleri*** (Hardy, 1983). **North Pacific Armorhead**. Museum specimens: CAS-ICH 26759 (off Pigeon Point during 1960, WI Follett, *as Pentacerosrichardsoni*); LACM 45682.001 (north of Piedras Blancas during 1991, moderate El Niño, identifier unknown, as *Pentacerospectoralis*). North Pacific species is *Pseudopentaceroswheeleri* and includes records of *Pentacerosrichardsoni* and *Pseudopentacerospectoralis* (Humphreys et al. 1989, Love et al. 2005).

##### Family OPLEGNATHIDAE – knifejaws

***Oplegnathusfasciatus*** (Temminck & Schlegel, 1844). **Barred Knifejaw**. Publication and Visual records: Ta et al. (2018) (southern Monterey Bay). Ta et al. (2018) reported the first records of the Western Pacific Ocean Barred Knifejaw in the Northeast Pacific Ocean, including at least two individual knifejaws at multiple sites in southern Monterey Bay between Dec 2014 and Sept 2015 (Del Monte Beach, San Carlos Beach, McAbee Pinnacle, and South Breakwater wall off Monterey Harbor entrance). These observations, along with others in Oregon and Washington, were spatially and temporally correlated with the arrival of Japanese tsunami marine debris. Additional observations by lead author Ta, of the assumedly lone fish, occurred between Oct and Dec 2018 (Monterey Herald, 10 Dec 2018). The Barred Knifejaw is native to Japan, Korea, and China in warm-temperate to tropical seas. These specimens observed from central California to Washington are unlikely to reproduce due to warmer temperature requirements; however, they can continue to grow in cold water. Transported with tsunami debris from the Western Pacific Ocean. Common name follows Ta et al. (2018).

##### Family EMBIOTOCIDAE – surfperches

***Amphistichusargenteus*** Agassiz, 1854. **Barred Surfperch**. Museum specimens: MLMLF1108 (Moss Landing Beach, D Varoujean); CAS-SU 33423 (Waddell Creek, WI Follett); CAS-SU 58374 (Carmel Beach, JC Briggs).

***Amphistichuskoelzi*** (Hubbs, 1933). **Calico Surfperch**. Museum specimens: MLMLF1110 (Marina Beach at Monterey Bay, D Varoujean); CAS-SU 5364 (San Simeon Bay, identified by “Gsm” likely George S. Myers, US Fish Commission); CAS-SU 34296 (Half Moon Bay, WI Follett).

***Amphistichusrhodoterus*** (Agassiz, 1854). **Redtail Surfperch**. Museum specimens: CAS-ICH 26972 (Muir Beach, Marin County, WI Follett); CAS-ICH (Sunset State Beach, K Oda, specimen pending accession at CAS, RN Lea, May 2018); MLMLF1111 (south jetty at Moss Landing, ME Anderson); CAS-SU 34289 (Half Moon Bay, WI Follett).

***Brachyistiusfrenatus*** Gill, 1862. **Kelp Perch**. Museum specimens: CAS-ICH 2191 (Stillwater Cove, identifier unknown); MLMLF1113 (off Sandholdt Pier at Moss Landing at Monterey Bay, L Talent); CAS-SU 58412 (Hopkins Marine Station, JC Briggs).

***Cymatogasteraggregata*** Gibbons, 1854. **Shiner Perch**. Museum specimens: CAS-ICH 25476 (San Simeon, CL Hubbs, LC Hubbs, WI Follett et al.); CAS-SU 49531 (Moss Beach, RF Ford); CAS-SU 51276 (Elkhorn Slough, D Cohen). Publication: Yoklavich et al. (1991) (Elkhorn Slough).

***Embiotocacaryi*** Agassiz, 1853. **Rainbow Seaperch**. Museum specimens: CAS-ICH 18204 (Elkhorn Slough, M Morten); CAS-ICH 25426 (Santa Cruz Wharf, WI Follett); CAS-SU 58379 (Hopkins Marine Station, JC Briggs). Previously recognized as *Hypsuruscaryi* (Agassiz, 1853). We follow naming convention of Longo et al. (2018).

***Embiotocajacksoni*** Agassiz, 1853. **Black Perch**. Museum specimens: MLMLF1117 (Kirby Park at Elkhorn Slough, E Yarberry); CAS-SU 22477 (Pacific Grove, John O. Snyder); CAS-SU 47496 (Elkhorn Slough, WC Freihofer). Publication: Yoklavich et al. (1991) (Hwy 1 Bridge, Dairy, and Kirby Park at Elkhorn Slough).

***Embiotocalateralis*** Agassiz, 1854. **Striped Seaperch**. Museum specimens: CAS-SU 16783 (Martin's Beach, south Half Moon Bay, GS Myers); CAS-SU 25783 (Moss Beach, WI Follett); CAS-SU 58427 (south of Carmel, JC Briggs).

***Hyperprosoponargenteum*** Gibbons, 1854. **Walleye Surfperch**. Museum specimens: CAS-SU 58388 (Carmel Beach, JC Briggs); CAS-SU 58389 (Hopkins Marine Station, JC Briggs); CAS-SU 58447 (Elkhorn Slough, JC Briggs). Publication: Yoklavich et al. (1991) (Hwy 1 Bridge, Dairy, Kirby Park, and Hudson’s Landing at Elkhorn Slough).

***Hyperprosoponellipticum*** (Gibbons, 1854). **Silver Surfperch**. Museum specimens: MLMLF1123 (off Skipper’s dock at Elkhorn Slough, B Antrim); CAS-SU 34294 (Half Moon Bay, WI Follett); CAS-SU 58386 (Carmel Beach, JC Briggs).

***Hypocritichthysanalis*** (Agassiz, 1861). **Spotfin Surfperch**. Museum specimens: CAS-ICH 25471 (San Simeon, CL Hubbs, LC Hunns, WI Follett et al.); CAS-ICH 27641 (Natural Bridges State Park, Santa Cruz, RR Rofen et al.); CAS-SU 34299 (Princeton Pier at Half Moon Bay, WI Follett). Previously recognized as *Hyperprosoponanale* Agassiz, 1861. We follow naming convention of Longo et al. (2018).

***Micrometrusaurora*** (Jordan & Gilbert, 1880). **Reef Perch**. Described from Monterey Bay; obtained from San Francisco market. Museum specimens: Syntypes [numerous specimens, including: ANSP 9272 (1), USNM 26996 (16)]; CAS-ICH 27642 (Natural Bridges State Park, Santa Cruz, RR Rofen et al.); CAS-ICH 212354 (Carmel Beach, BW Walker, A Flechsig); CAS-SU 48890 (east of Point Piedras Blancas, M Bradbury). Publication: Jordan and Gilbert (1880j) (original description).

***Micrometrusminimus*** (Gibbons, 1854). **Dwarf Perch**. Museum specimens: CAS-SU 15995 (Monterey Bay, K Stanton); CAS-SU 19316 (Elkhorn Slough, D Cohen); CAS-SU 58411 (Hopkins Marine Station, JC Briggs). Publication: Yoklavich et al. (1991) (Hwy 1 Bridge and Dairy at Elkhorn Slough).

***Phanerodonatripes*** (Jordan & Gilbert, 1880). **Sharpnose Seaperch**. Described from Monterey Bay and Santa Cruz; obtained from San Francisco market. Museum specimens: Syntypes [numerous specimens, including: ANSP 9203 (1), USNM 26987 (orig. 80, now 7)]; CAS-ICH 51865 (Big Sur, RN Lea). Publications: Jordan and Gilbert (1880k) (original description); Lea (1972) (Monterey area).

***Phanerodonfurcatus*** Girard, 1854. **White Seaperch**. Museum specimens: CAS-ICH 20377 (San Simeon, CL Hubbs, LC Hubbs, WI Follett et al.); CAS-ICH 25427 (Santa Cruz, WI Follett); CAS-SU 51269 (Elkhorn Slough, WC Freihofer). Publication: Yoklavich et al. (1991) (Hwy 1 Bridge, Dairy, and Kirby Park at Elkhorn Slough).

***Phanerodonvacca*** (Girard, 1855). **Pile Perch**. Museum specimens: LACM 4370 (Moss Beach, BW Halstead); LACM 8252 (San Simeon Point, BW Walker); CAS-SU 58393 (Carmel Beach, JC Briggs); UMMZ 142380 (Elkhorn Slough, R Bolin and CL Hubbs). Publication: Yoklavich et al. (1991) (Hwy 1 Bridge, Dairy, and Kirby Park at Elkhorn Slough). Previously recognized as *Damalichthysvacca* Girard, 1855. We follow naming convention of Longo et al. (2018).

***Rhacochilustoxotes*** Agassiz, 1854. **Rubberlip Seaperch**. Museum specimens: LACM 52230.008 (off San Simeon Point, JE Bleck); MLMLF1136 (Kirby Park at Elkhorn Slough, B Antrim); CAS-SU 12034 (Pacific Grove, EC Starks). Publication: Yoklavich et al (1991) (Hwy 1 Bridge and Dairy at Elkhorn Slough).

***Zalembiusrosaceus*** (Jordan & Gilbert, 1880). **Pink Seaperch**. Museum specimens: CAS-ICH 37178 (off Santa Cruz, T Iwamoto); CAS-ICH 37530 (west of Point Año Nuevo, KR Hakanson); CAS-SU 19135 (Monterey Bay, D Cohen).

##### Family POMACENTRIDAE – damselfishes

***Chromispunctipinnis*** (Cooper, 1863). **Blacksmith**. Museum specimens: MLMLF1148 (Monastery Beach during Nov 1970, E Starks); CAS-SU 15997 (Monterey Bay during 1937, RL Bolin). Publications: Starks (1919) (Monterey Bay during 1918); Phillips (1961) (Carmel Bay during 1959, El Niño); Radovich (1961) (Monterey Bay during 1959, El Niño). Categorized as occurring during warm-water events (e.g., El Niño).

***Hypsypopsrubicundus*** (Girard 1854). **Garibaldi**. Described from Monterey. Museum specimens: Syntypes [MCZ 14825 (1, San Diego), USNM 484 (2, San Diego)]. Publications: Girard (1854) (original description); Girard (1858a) (2 adults from “Monterey, Cal.”, refers to type specimens). Original description (and Girard 1858a) states “From Monterey, Cal.”; however, only specimens from San Diego apparently exist (USNM 484). Observations are extremely rare in MBNMS; no other museum specimens are known. During Jun 2016 and Feb 2017, a single juvenile Garibaldi was observed in Monterey Bay Aquarium’s (MBA) outdoor Great Tide Pool (MBA video imagery, pers. comm. Andrew Morgan, MBA Dive Officer/Exhibit Dive Coordinator). We cannot rule out the possibility the Garibaldi specimen was released from the aquarium kelp tank by way of the filtration system (i.e., as larvae). We categorize the listing here as Historic, and possibly occurring during warm-water events (e.g., El Niño).

##### Family LABRIDAE – wrasses

***Oxyjuliscalifornica*** Günther, 1861. **Señorita**. Described from Monterey. Museum specimens: Syntypes [USNM 706-707 (2,1)]; LACM 4922 (San Simeon, ES Hobson); MLMLF1164 (off Skipper’s Dock at Elkhorn Slough, ME Anderson); CAS-SU 3895 (Pacific Grove, JO Snyder); CAS-SU 4156 (Monterey, CH Gilbert). Publication: Günther (1861) (original description).

***Semicossyphuspulcher*** (Ayres, 1854). **California Sheephead**. Museum specimen: USNM 110755 (Monterey, DS Jordan, date unknown). Publications: Phillips (1932a) (Monterey Bay during 1931, El Niño); Radovich (1961) (Point Pinos during 1958, El Niño). Categorized as occurring during warm-water events (e.g., El Niño).

##### Family BATHYMASTERIDAE – ronquils

***Rathbunellaalleni*** Gilbert, 1904. **Stripefin Ronquil**. Described from Monterey Bay. Museum specimens: Holotype (CAS-SU 8415); Paratype [CAS-SU 8416 (Monterey Bay)]; CAS-ICH 57636 (Monterey Bay, AC Matarese); CAS-SU 23016 (Pacific Grove, AC Matarese). Publications: Gilbert (1904) (original description); Stevenson and Matarese (2005) (Monterey Bay and other).

***Ronquilusjordani*** (Gilbert, 1889). **Northern Ronquil**. Museum specimens: MLMLF1169 (Monterey Bay, G McDonald); OS 7632 (off Monterey Bay, J Cobb); CAS-SU 36071 (Monterey Bay, J Waler and RL Bolin).

##### Family ZOARCIDAE – eelpouts

***Bothrocarabrunneum*** (Bean, 1890). **Twoline Eelpout**. Museum specimens: CAS-ICH 25990 (off Pescadero Point, ME Anderson); CAS-ICH 47395 (Monterey Canyon, RN Lea); CAS-ICH 55411 (off Point Sur, ME Anderson). Publication: Burton and Lundsten (2008) (Davidson Seamount, based on imagery). Visual records: MBARI/NOAA T427-02, MBARI/NOAA D943-07, and MBARI/NOAA T946-05 (Davidson Seamount, EJ Burton, L Kuhnz, and L Lundsten).

***Bothrocaramolle*** Bean, 1890. **Soft Eelpout**. Museum specimen: CAS-ICH 60291 (west of Point Año Nuevo, ME Anderson); UW 46821 (east of Sur Ridge, RC Harrison); UW 113723 (Smooth Ridge at Monterey Bay, JR Hoff).

***Eucryphycuscalifornicus*** (Starks & Mann, 1911). **Persimmon Eelpout**. Museum specimens: CAS-ICH 17623 (Monterey Canyon, GM Cailliet and RN Lea); CAS-ICH 38671 (Monterey Bay, RG Kliever). Publication: Cailliet and Lea (1977) (Monterey Bay).

***Lycenchelyscallista*** Anderson, 1995. **eelpout**. Described from off Point Sur. Museum specimens: Holotype (CAS-ICH 55412); Paratypes [CAS-ICH 55062 (2, off Point Sur, ME Anderson), CAS-ICH 80630 (29, off Point Sur, ME Anderson); SIO 84-249 (21, off Point Sur), SIO 85-51 (22, off Point Sur)]. Publication: Anderson (1995) (original description). No official common name.

***Lycenchelyscamchatica*** (Gilbert & Burke, 1912). **Kamchatka Eelpout**. Museum specimens: CAS-ICH 31495 (Monterey Canyon, ME Anderson); CAS-ICH 55406 and CAS-ICH 56234 (off Point Sur, ME Anderson). Publication: Anderson et al. (1979) (Monterey Canyon, CAS-ICH 31495).

***Lycenchelyscrotalinus*** (Gilbert, 1890). **Snakehead Eelpout**. Museum specimens: CAS-ICH 55063 and CAS-ICH 55407 (off Point Sur, ME Anderson); CAS-ICH 57467 (Monterey Canyon, ME Anderson). Previously recognized as *Embryxcrotalinus*. We follow Anderson (1995), Anderson and Fedorov (2004), and current usage in *Catalog of Fishes* (Eschmeyer et al. 2017); with placement in *Lycenchelys*.

***Lycenchelysjordani*** (Evermann & Goldsborough, 1907). **Shortjaw Eelpout**. Museum specimen: CAS-ICH 78979 (Point Sur, ME Anderson).

***Lycenchelysmicropora*** Andriashev, 1955. **Manytoothed Eelpout**. Museum specimens: CAS-ICH 234380 (off Monterey Bay, ME Anderson); UW 40727 (off Point Sur, W Wakefield and ME Anderson). Common name follows Mecklenburg et al. (2002).

***Lycenchelysmonstrosa*** Anderson, 1982. **eelpout**. Museum specimen: CAS-ICH 233977 (off Monterey Bay, ME Anderson). No official common name.

***Lycodapusdermatinus*** Gilbert, 1896. **Looseskin Eelpout**. Museum specimens: CAS-ICH 31493 (Monterey Canyon, ME Anderson); CAS-ICH 55403 (off Point Sur, ME Anderson); MLMLF1187 (off Point Sur, ME Anderson).

***Lycodapusfierasfer*** Gilbert, 1890. **Blackmouth Eelpout**. Museum specimens: CAS-ICH 35989 (Monterey Canyon, ME Anderson); CAS-ICH 58480 (SW of Point Sur, RN Lea); CAS-ICH 88443 (Monterey Bay, G Cailliet). Publication: Burton and Lundsten (2008) (Davidson Seamount, based on imagery). Visual record: MBARI/NOAA T426-05 (Davidson Seamount, L Kuhnz).

***Lycodapusmandibularis*** Gilbert, 1915. **Pallid Eelpout**. Described from Monterey Bay. Museum specimens: Holotype (USNM 75823); Paratypes [CAS-SU 22990 (1, Monterey Bay), CAS-SU 25765 (5, now 4, Monterey Bay); USNM 149514 (2, Monterey Bay)]. Publications: Gilbert (1915) (original description); Burton and Lundsten (2008) (Davidson Seamount, based on imagery). Visual record: MBARI/NOAA T946-01 (Davidson Seamount, L Lundsten).

***Lycodapuspachysoma*** Peden and Anderson, 1981. **Stout Eelpout**. Museum specimen: UW 47335 (within Davidson Seamount Management Zone, J Hoff). Common name follows Mecklenburg et al. (2002).

***Lycodapuspsarostomatus*** Peden and Anderson, 1981. **Specklemouth Eelpout**. Museum specimen: CAS-ICH 58902 (Point Pinos, ME Anderson). Common name follows Mecklenburg et al. (2002).

***Lycodescortezianus*** (Gilbert, 1890). **Bigfin Eelpout**. Museum specimens: CAS-ICH 37485 (west of Santa Cruz, T Iwamoto); CAS-ICH 39892 (west of Point Lobos, WC Ruark); MLMLF1171 (Monterey Bay, ME Anderson).

***Lycodesdiapterus*** Gilbert, 1892. **Black Eelpout**. Museum specimens: CAS-ICH 29299 and CAS-ICH 29945 (Monterey Bay, ME Anderson); CAS-ICH 37489 (Santa Cruz, T Iwamoto).

***Lycodespacificus*** (Collett, 1879). **Blackbelly Eelpout**. Museum specimens: CAS-ICH 28873 and CAS-ICH 30881 (Monterey Bay, ME Anderson); USNM 227179 (Monterey, ME Anderson).

***Lyconemabarbatum*** Gilbert, 1896. **Bearded Eelpout**. Described from outside Monterey Bay. Museum specimens: Lectotype (USNM 48582, Lectotype established in Jordan and Evermann 1900, and traced to USNM 48582 by Springer and Anderson 1997); Paralectotypes including [CAS-SU 3627 (1), CAS-SU 69673 (4), USNM 53036 (1)]. Publications: Gilbert (1896) (original description); Jordan and Everman (1900) (lectotype established); Springer and Anderson (1997).

***Melanostigmapammelas*** Gilbert, 1896. **Midwater Eelpout**. Described from Monterey Bay. Museum specimens: Holotype (USNM 53034, missing); Paratypes [USNM 48599 (nearly disintegrated), USNM 53034, CAS-SU 4000]; MLMLF1212 (Monterey Bay, ME Anderson). Publications: Gilbert (1896) (original description); Gilbert (1915) (Monterey Bay).

***Pachycarabulbiceps*** (Garman, 1899). **Snubnose Eelpout**. Publications: Anderson and Peden (1988) (off Queen Charlotte Islands, British Columbia, to the Gulf of Panama, North Pacific, at depths of 2,601 to 4,000 m); Burton and Lundsten (2008), and Lundsten et al. (2009) (Davidson Seamount, based on imagery). Visual records: MBARI/NOAA D947-05 (Davidson Seamount, Jacobson Stout et al. 2017); MBARI D990-02 and MBARI D438-05 (Monterey Canyon, Jacobson Stout et al. 2017). Common name follows Mecklenburg et al. (2002).

***Pachycarakarenae*** Anderson, 2012. **eelpout**. Described from Monterey Bay. Museum specimens: Holotype (CAS-ICH 233971); Paratypes (CAS-ICH 233972, USNM 405384). Publication: Anderson (2012) (original description). No official common name.

##### Family STICHAEIDAE – pricklebacks

***Anoplarchuspurpurescens*** Gill, 1861. **High Cockscomb**. Museum specimens: CAS-ICH 7507 (Moss Beach, RR Harry); CAS-ICH 25196 (Carmel Bay, WI Follett); CAS-ICH 232759 (Point Lobos, RL Bolin); CAS-ICH 32985 (north of San Simeon Beach, WN Eschmeyer and SG Poss).

***Cebidichthysviolaceus*** (Girard, 1854). **Monkeyface Prickleback**. Museum specimens: CAS-ICH 18297 (Moss Beach, CL Hubbs); CAS-ICH 18662 (Pacific Grove, RR Harry); CAS-ICH 33004 (north of San Simeon, WN Eschmeyer and SG Poss); CAS-SU 47498 (Elkhorn Slough, WC Freihoffer).

***Chirolophisnugator*** (Jordan & Williams, 1895). **Mosshead Warbonnet**. Museum specimens: CAS-ICH 25893 (Pacific Grove, WI Follett); LACM 6608.005 (off San Simeon, ES Hobson); LACM 52248.012 (Jade Cove, ES Hobson); CAS-SU 64363 (Point Lobos tidepool, RL Bolin).

***Ernogrammuswalkeri*** Follett and Powell, 1988. **Masked Prickleback**. Described from west of San Simeon Point. Museum specimens: Holotype (CAS-ICH 56198); numerous Paratypes including [CAS-ICH 27156 (Monterey), CAS-ICH 48094 (off Pacific Grove), CAS-ICH 57410 (US Coast Guard breakwater, Monterey), CAS-ICH 57635 (Monterey Bay), SIO 72-86 (north of San Simeon Point)]. Publication: Follett and Powell (1988) (original description).

***Esselenichthyscarli*** (Follett & Anderson, 1990). **Threeline Prickleback**. Described from Santa Barbara (holotype), Monterey, and south to Baja California. Museum specimens: Paratype (CAS-ICH 25892); SIO 53-193 (Pacific Grove, WI Follett). Publication: Follett and Anderson (1990) (original publication).

***Kasatkiaseigeli*** Posner and Lavenberg, 1999. **Sixspot Prickleback**. Museum specimen: SIO 03-92 (off Pacific Grove at Monterey Bay, RN Lea).

***Phytichthyschirus*** (Jordan & Gilbert, 1880). **Ribbon Prickleback**. Described from Point Pinos, Pacific Grove. Museum specimens: Lectotype (USNM 27175, Lectotype selected by Springer and Anderson 1997); Paralectotypes [USNM 200384 (1), 336453 (2)]; CAS-SU 48291 (Soberanes Point, RL Bolin); CAS-SU 48301 (Soberanes Point, WC Freihofer). Publications: Jordan and Gilbert (1880e) (original description); Springer and Anderson (1997).

***Plagiogrammushopkinsii*** Bean, 1894. **Crisscross Prickleback**. Described from Monterey Bay. Museum specimens: Holotype (USNM 44721); CAS-ICH 54642 (Pescadero Point, RL Bolin); CAS-SU 21199 (Pacific Grove, JO Snyder); CAS-SU 35302 (Point Lobos, RL Bolin and WE Ripley). Publication: Bean (1894) (original description).

***Plectobranchusevides*** Gilbert, 1890. **Bluebarred Prickleback**. Museum specimen and Publication: USNM 77460 (off Point Pinos, “Albatross” Explorations on the California Coast during 1904, Gilbert 1915).

***Xiphisteratropurpureus*** (Kittlitz, 1858). **Black Prickleback**. Museum specimens: CAS-ICH 14266 (between Yankee and Soberanes Points, RL Bolin and Marr); CAS-SU 16037 (Monterey Bay tidepool, A Calhoun); CAS-SU 58371 (south of Yankee Point, JC Briggs).

***Xiphistermucosus*** (Girard, 1858). **Rock Prickleback**. Museum specimens: CAS-ICH 18705 (Carmel Bay, RR Harry); CAS-ICH 18842 (Moss Beach, RR Harry); CAS-SU 16038 (Monterey Bay tidepool, A Calhoun); CAS-SU 48902 (east of Point Piedras Blancas, M Bradbury).

##### Family PHOLIDAE – gunnels

***Apodichthysflavidus*** Girard, 1854. **Penpoint Gunnel**. Museum specimens: CAS-ICH 11948 (Moss Beach, RR Harry); CAS-ICH 25894 (Pacific Grove, WI Follett); MLMLF1240 (off Skipper’s Dock at Elkhorn Slough, ME Anderson); CAS-SU 47505 (Monterey, WC Freihofer); CAS-SU 58470 (Carmel Beach, JC Briggs).

***Apodichthysfucorum***. Jordan and Gilbert, 1880. **Rockweed Gunnel**. Described from Point Pinos. Museum specimens: Lectotype (USNM 26994, Lectotype selected by Springer and Anderson 1997); numerous Paralectotypes including [ANSP 10501 (Monterey); USNM 335151 (Monterey, 37)]; CAS-ICH 18693 (Moss Beach, RR Harry); CAS-ICH 26108 (Pacific Grove, WI Follett); CAS-ICH 47390 (Carmel kelp bed, RN Lea); CAS-ICH 32995 (north of San Simeon, WN Eschmeyer and SG Poss). Publication: Jordan and Gilbert (1880e) (original description).

***Pholisclemensi*** Rosenblatt, 1964. **Longfin Gunnel**. Publication and Visual record: (Point Lobos, Pillar Point, Kline et al. 2013).

***Pholisornata*** (Girard, 1854). **Saddleback Gunnel**. Museum specimens: CAS-ICH 20238 and CAS-ICH 20239 (Moss Beach tidepool, identifier unknown); CAS-SU 63688 (Carmel Beach, RL Bolin).

***Pholisschultzi*** Schultz, 1931. **Red Gunnel**. Museum specimens: LACM 6608.006 (off San Simeon Point, ES Hobson); LACM 7942 (off San Simeon Point, B Walker); SIO 72-86 (north of San Simeon Point, R Rosenblatt).

***Ulvicolasanctaerosae*** Gilbert and Starks, 1897. **Kelp Gunnel**. Museum specimens: CAS-ICH 14989 (Pacific Grove, RN Lea); CAS-ICH 39860 (Carmel Bay, RN Lea); UCM 6639 (Half Moon Bay, TP Maslin).

##### Family ANARHICHADIDAE – wolffishes

***Anarrhichthysocellatus*** Ayres, 1855. **Wolf-eel**. Museum specimens: ANSP 25047 (Pacific Grove, H Heath); CAS-ICH 31229 (Monterey Bay, WC Freihofer); CAS-SU 12610 (Pacific Grove, EC Starks).

##### Family ZAPRORIDAE – prowfishes

***Zaprorasilenus*** Jordan, 1896. **Prowfish**. Museum specimens: CAS-ICH 30693 (Monterey Bay, ME Anderson and GM Cailliet); CAS-ICH 47403 (between Bixby Creek and Carmel Bay, RN Lea); MLMLF1254 (Monterey Bay, GM Cailliet and ME Anderson). Publication: Cailliet and Anderson (1975) (Monterey Bay, CAS-ICH 30693).

##### Family SCYTALINIDAE – graveldivers

***Scytalinacerdale*** Jordan and Gilbert, 1880. **Graveldiver**. Museum specimens: CAS-ICH 52435 (Moss Beach, D Catania); CAS-SU 58431 (south of Carmel, JC Briggs); UMMZ 36955 (Point Lobos, CL Hubbs).

##### Family CHIASMODONTIDAE – swallowers

***Chiasmodonniger*** Johnson, 1864. **Black Swallower**. Museum specimens: UW 48697 (outside MBNMS, west of Ragged Point during Oct 1999, RN Clark, NMFS, 1034 m, included here due to proximity); UW 48713 (west of Carmel during Oct 1999, RN Clark, NMFS, 1084 m). Publication: Lauth (2000) (UW specimens). Visual records: MBARI D241-06 and MBARI D0451-07 (Monterey Canyon, Jacobson Stout et al. 2017). Previously recognized as *Chiasmodonsubniger* Garman, 1899. We follow naming convention of Prokofiev and Kukuev (2009), and Prokofiev (2014) where *Chiasmodonsubniger* Garman, 1899 is considered a synonym of *Chiasmodonniger* Johnson, 1864.

***Kaliindica*** Lloyd, 1909. **Pacific Sandfish**. Museum specimen: UW 48679 (west of Carmel during Oct 1999, D Kamikawa, 1146 m).

##### Family TRICHODONTIDAE – sandfishes

***Trichodontrichodon*** (Tilesius, 1813). **Pacific Sandfish**. Publication: Ayres 1860a. *Trichodonlineatus* Ayres 1860 is considered a synonym of *Trichodontrichodon* (Tilesius 1813). The type description of *Trichodonlineatus* by Ayres (1860) was based on one specimen from a “market in San Francisco.” The holotype is presumably lost (Eschmeyer et al. 2017). The capture location is unknown; however, in 1860 captures were probably fairly close to San Francisco, and sold within 1 day of capture. Specimen likely occurred in, or traversed, MBNMS boundary. No other records found within MBNMS. Categorized as Historic.

##### Family AMMODYTIDAE – sand lances

***Ammodyteshexapterus*** Pallas, 1814. **Pacific Sand Lance**. Museum specimens: CAS-ICH 14284 (Rodeo Beach, Marin County, L Dempster); MLMLF1288 (Elkhorn Slough main channel between jetties, ME Anderson); MLMLF1290 (Monterey Bay, M Stevenson).

##### Family URANOSCOPIDAE – stargazers

***Kathetostomaaverruncus*** Jordan and Bollman, 1890. **Smooth Stargazer**. Museum specimens: CAS-ICH 56960 (Monterey Bay during 1984, GM Cailliet); CAS-ICH 57637 (Monterey Bay during 1985, RN Lea); LACM 32181.001 (off Point Piedras Blancas during 1960, L Pinkas); MLMLF1293 (off Soquel Point at Monterey Bay during 1984, GM Cailliet). Typically a southern species, museum specimens collected shortly after warm water events (i.e., 1983-1984, 1957-1959). Categorized as occurring during warm-water events (e.g., El Niño).

##### Family BLENNIIDAE – combtooth blennies

***Hypsoblenniusgentilis*** (Girard, 1854). **Bay Blenny**. Described from Monterey. Museum specimens: Holotype (USNM 489); CAS-ICH 13710 (Monterey Breakwater during 1970, CL Hubbs); CAS-ICH 243620 (Elkhorn Slough during 2016, G Longo); SIO 93-189 (Elkhorn Slough during 1993, RN Lea). Publication: Girard (1854) (original description).

***Hypsoblenniusgilberti*** (Jordan, 1882). **Rockpool Blenny**. Museum specimen: LACM 38727.001 (Carmel Bay during 1976, RN Lea).

***Hypsoblenniusjenkinsi*** (Jordan & Evermann, 1896). **Mussel Blenny**. Museum specimens: CAS-ICH 56607 (Santa Cruz Municipal Wharf during 1985, G. McDonald, examined by RN Lea); CAS-ICH 242460 (Monterey Commercial Wharf during 2016, El Niño, G Longo). Publication: Nelson (1986) (Santa Cruz Municipal Wharf during 1985, CAS-ICH 56607). Typically a southern species, museum specimens collected shortly after warm water events (i.e., 1983-1984). Categorized as occurring during warm-water events (e.g., El Niño).

##### Family CLINIDAE – kelp blennies

***Gibbonsiaelegans*** (Cooper, 1864). **Spotted Kelpfish**. Museum specimens: ANSP 16302 (Pacific Grove during 1897, El Niño, H Heath); CAS-ICH 2190 (Pebble Beach, Monterey County during 1928, RR Harry); LACM 9950.038 (San Simeon during 1963, BW Walker). Typically a southern species, south of Point Piedras Blancas (Miller and Lea 1972). Categorized as occurring during warm-water events (e.g., El Niño).

***Gibbonsiametzi*** Hubbs, 1927. **Striped Kelpfish**. Described from Pacific Grove. Museum specimens: Holotype (UMMZ 55004); CAS-ICH 7554 (Monterey, RR Harry); CAS-ICH 27644 (Natural Bridges State Park, E Byron); CAS-ICH 232453 (Soberanes Point, RN Lea); CAS-SU 15488 (Elkhorn Slough, CL Hubbs and RL Bolin). Publication: Hubbs (1927) (original description).

***Gibbonsiamontereyensis*** Hubbs, 1927. **Crevice Kelpfish**. Described from Pacific Grove. Museum specimens: Holotype (UMMZ 55003); CAS-ICH 25194 (Carmel Bay, WI Follett); CAS-ICH 37922 (NW of San Simeon, W Boyd); CAS-SU 48898 (1.75 miles east of Point Piedras Blancas, M Bradbury). Publication: Hubbs (1927) (original description).

***Heterostichusrostratus*** Girard, 1854. **Giant Kelpfish**. Museum specimens: MLMLF1307 (east of Hwy 1 Bridge at Elkhorn Slough, B Antrim); CAS-SU 5555 (Pacific Grove, EC Starks); CAS-SU 15136 (Del Monte Beach, Monterey, CL Hubbs).

##### Family CHAENOPSIDAE – tube blennies

***Neoclinusblanchardi*** Girard, 1858. **Sarcastic Fringehead**. Museum specimens: CAS-ICH 42556 (Carmel Bay, D Gotshall); MLMLF1314 (Elkhorn Slough between jetties, R Helm); CAS-SU 2288 (Pacific Grove, C Hubbs); CAS-SU 19183 (Monterey Bay, D Cohen and WC Freihofer).

***Neoclinusstephensae*** Hubbs, 1953. **Yellowfin Fringehead**. Museum specimens: CAS-ICH 14403 (Monterey Harbor during 1964, DC Powell). Visual record: SIMoN (2018) (Monterey Harbor during Mar 2007 and 2010, El Niños, SI Lonhart). Categorized as occurring during warm-water events (e.g., El Niño).

***Neoclinusuninotatus*** Hubbs, 1953. **Onespot Fringehead**. Museum specimens: Paratypes [Pacific Grove: CAS-ICH 7414, CAS-SU 2285, CAS-SU 23022]; CAS-ICH 26298 (Monterey, WI Follett). Publications: Hubbs (1953) (original description); Yoklavich et al. (1991) (Hwy 1 Bridge at Elkhorn Slough).

##### Family ICOSTEIDAE – ragfishes

***Icosteusaenigmaticus*** Lockington, 1880. **Ragfish**. Museum specimens: CAS-ICH 47399 (Monterey Bay, RN Lea); CAS-ICH 53094 (Santa Cruz Municipal Wharf during 1948, WI Follett); LACM 32682.001 (San Simeon Bay during 1972, identifier unknown); CAS-SU 58300 (off Point Sur during 1961, JB Phillips). Publications: Snyder (1913) (Pacific Grove); Thompson (1921b) (Monterey).

##### Family GOBIESOCIDAE – clingfishes

***Gobiesoxmaeandricus*** (Girard, 1858). **Northern Clingfish**. Museum specimens: CAS-SU 1681 (Monterey, EC Starks); CAS-SU 15212 (Pacific Grove tidepool, JC Briggs); CAS-SU 58434 (south of Carmel, JC Briggs).

***Rimicolamuscarum*** (Meek & Pierson, 1895). **Kelp Clingfish**. Described from Monterey Bay. Museum specimens: Holotype (CAS-SU 3030); Paratype [USNM 48875 (1, Monterey Bay)]; MLMLF0540 (Carmel Point tidepools, GE Kukowski); MLMLF0542 (Monterey Bay drift kelp, ME Anderson). Publication: Meek and Pierson (1895) (original description).

##### Family GOBIIDAE – gobies

***Acanthogobiusflavimanus*** (Temminck & Schlegel, 1845). **Yellowfin Goby**. Museum specimens: MLMLF1328 (Elkhorn Slough, D Varoujean); MLMLF1329 (Kirby Park at Elkhorn Slough, D Varoujean); SIO 10-96 (Elkhorn Slough, RN Lea). Publication: Yoklavich et al. (1991) (station location from Barry 1983, Hudson’s Landing at Elkhorn Slough). Species “inadvertently introduced from the Orient” (Miller and Lea 1972).

***Clevelandiaios*** (Jordan & Gilbert, 1882). **Arrow Goby**. Museum specimens: CAS-ICH 19629 (Elkhorn Slough, CL Hubbs, RL Bolin et al.); CAS-SU 15029 (Elkhorn Slough, C Hubbs); CAS-SU 58450 (Elkhorn Slough, JC Briggs). Publication: Yoklavich et al. (1991) (Hwy 1 Bridge and Hudson’s Landing at Elkhorn Slough).

***Gillichthysmirabilis*** Cooper, 1864. **Longjaw Mudsucker**. Museum specimens: MLMLF1340 (near yacht club at Elkhorn Slough, E Yarberry); MLMLF1341 (Kirby Park at Elkhorn Slough, LT Ackerman); MLMLF1342 (Kirby Park at Elkhorn Slough, B Stewart). Publications: Cailliet et al. (1977) (larvae at Elkhorn Slough); Yoklavich et al. (1991) (station location from Barry 1983, Hudson’s Landing at Elkhorn Slough).

***Lepidogobiuslepidus*** (Girard, 1858). **Bay Goby**. Museum specimens: CAS-ICH 56232 (Monterey Bay, ME Anderson); MLMLF1344 (Kirby Park at Elkhorn Slough, GE Kukowski). Publications: Yoklavich et al. (1991) (Hwy 1 Bridge, Dairy, and Kirby Park at Elkhorn Slough).

***Lethopsconnectens*** Hubbs, 1926. **Halfblind Goby**. Described from Carmel Bay. Museum specimens: Holotype (UMMZ 63281); Paratypes [UMMZ 63282 (2)]; LACM 52277.01 (west of San Simeon Point, ES Hobson, J Bleck, T Chess, L Richards, J Kastendiek, A Harrington, J Morin). Publication: Hubbs (1926) (original description).

***Lythrypnusdalli*** (Gilbert, 1890). **Bluebanded Goby**. Museum specimen: CAS-ICH 54911 (Monterey Coast Guard Breakwater during 1983, warm-water event, RN Lea). Categorized as occurring during warm-water events (e.g., El Niño).

***Lythrypnuszebra*** (Gilbert, 1890). **Zebra Goby**. Museum specimen: CAS-ICH 31994 (Monastery Beach during 1975, ME Anderson).

***Rhinogobiopsnicholsii*** (Bean, 1882). **Blackeye Goby**. Museum specimens: MLMLF1350 (Monterey Breakwater, ME Anderson); MLMLF1352 (Elkhorn Slough, ME Anderson); CAS-SU 15116 (south of Malpaso Creek, C Hubbs).

***Typhlogobiuscaliforniensis*** Steindachner, 1879. **Blind Goby**. Museum specimen: UCLA W 62-92 (cove north of San Simeon Point, identifier unknown, specimen pending accession at SIO, pers. comm. HJ Walker, Oct 2017).

##### Family LUVARIDAE – louvars

***Luvarusimperialis*** Rafinesque, 1810. **Louvar**. Museum specimens: CAS-ICH 13245 (Santa Cruz during Sept 1945, WM Chapman); CAS-SU 14224 (Monterey Bay during Nov 1939, RL Bolin).

##### Family SPHYRAENIDAE – barracudas

***Sphyraenaargentea*** Girard, 1854. **Pacific Barracuda**. Museum specimen: USNM 109985 (Monterey, DS Jordan, date unknown). Publications: Phillips (1932a) (Monterey Bay during 1931, El Niño); Radovich (1961) (Monterey during 1958, El Niño). Common south of Morro Bay (Miller and Lea 1972). Categorized as occurring during warm-water events (e.g., El Niño).

##### Family GEMPYLIDAE – snake mackerels

***Ruvettuspretiosus*** Cocco, 1833. **Oilfish**. Museum specimen: CAS-ICH 54919 (40 miles west of Monterey during 1983, J Hardwick). Typically occurs in warmer water; however, can move north during El Niño years. Categorized as occurring during warm-water events (e.g., El Niño).

##### Family TRICHIURIDAE – cutlassfishes

***Aphanopusintermedius*** Parin, 1983. **Intermediate Scabbardfish**. Museum specimens (previously recognized as *A.carbo*): CAS-ICH 40254 (west of Pigeon Point, B Antrim and ME Anderson); CAS-ICH 42570 (off Pigeon Point, RN Lea); CAS-ICH 57643 (west of Santa Cruz, RN Lea). Common name follows Love et al. (2005).

***Benthodesmuspacificus*** Parin and Becker, 1970. **North Pacific Frostfish**. Museum specimen: CAS-ICH 30692 (Moss Landing during 1968, ME Anderson).

***Lepidopusfitchi*** Rosenblatt and Wilson, 1987. **Pacific Scabbardfish**. Museum specimen: CAS-ICH 67930 (off Montara during 1962, identifier unknown).

##### Family SCOMBRIDAE – mackerels

***Auxisrochei*** (Risso, 1810). **Bullet Mackerel**. Museum specimen: CAS-ICH 56944 (between Half Moon Bay and San Francisco Bay during 1984, El Niño, RN Lea). Eastern Pacific population of *A.rochei* has been described as a subspecies (*Auxisrocheieudorax* Collette and Aadland, 1996), including record within MBNMS (CAS-ICH 56944). Typically occurs in warmer seas; however, can move north during El Niño years. Categorized as occurring during warm-water events (e.g., El Niño).

***Katsuwonuspelamis*** (Linnaeus, 1758). **Skipjack Tuna**. Publications: Radovich (1961) (off Davidson Seamount); Lea (1997) (off Lopez Point). Found off Davidson Seamount (distance not specified) during 1957 El Niño (Radovich 1961). School observed surface feeding on Pacific sardine off Lopez Point on 25 Oct 1997; four captured (El Niño, Lea 1997). Typically occurs in warmer seas; however, can move north during El Niño years. Categorized as occurring during warm-water events (e.g., El Niño). There are no museum specimens collected within MBNMS; however, large fish are less commonly stored in museums.

***Sardachiliensis*** (Cuvier, 1832). **Pacific Bonito**. Publications: Phillips (1932a) (Monterey Bay during 1931, El Niño); Fast (1957) (Monterey Bay during 1956, warm-water event); Radovich (1961) (Monterey during 1959, El Niño). Categorized as occurring during warm-water events (e.g., El Niño).

***Scomberjaponicus*** Houttuyn, 1782. **Pacific Chub Mackerel**. Museum specimen: UMMZ 176337 (Pacific Grove, 1959, El Niño, RM Bailey). Publications: Phillips (1932a) (Monterey Bay during 1931 El Nino); Phillips (1937) (near Pacific Grove). Categorized as occurring during warm-water events (e.g., El Niño).

***Scomberomorusconcolor*** (Lockington 1879). **Gulf Sierra**. Described from San Francisco Market, and “probably taken…in Monterey Bay.” Museum specimens: Holotype (CAS-ICH lost specimen in 1906); USNM 27205 (Soquel, DS Jordan, date unknown). Publications: Lockington (1879) (original description); Phillips (1932b) (Monterey during 1931, El Niño). Categorized as occurring during warm-water events (e.g., El Niño).

***Thunnusalalunga*** (Bonnaterre, 1788). **Albacore**. Museum specimen: USNM 270550 (San Simeon during 1941, identifier unknown). Publications: Andrews (1927) (off Point Sur during Sept 1925 and fall 1926, El Niño); Phillips (1931a) (SW of Point Pinos during 1930, El Niño); Phillips (1932a) (south of Monterey Bay during 1931, El Niño); Fitch (1949) (Davidson Seamount); Fast (1957) (Monterey Bay area during Sept 1956, warm-water event); Radovich (1961) (Monterey during 1931, El Niño); CDFW (2017) [Sport Fishing Record (Santa Cruz during 1997, El Niño)]. Categorized as occurring during warm-water events (e.g., El Niño).

***Thunnusalbacares*** (Bonnaterre, 1788). **Yellowfin Tuna**. Publication: Fast (1957) (Monterey Bay during 1956, warm-water event). Categorized as occurring during warm-water events (e.g., El Niño). There are no catalogued records collected within MBNMS; however, large fish are less commonly stored in museums.

***Thunnusobesus*** (Lowe, 1839). **Bigeye Tuna**. Publication: Radovich (1961). Published range is Peru to Iron Springs, Washington; considered rare off California (Miller and Lea 1972). Considered a tropical species but occurs north during warm-water events. Radovich (1961) noted movement north during 1959 El Niño (off Cape Mendocino, CA and Iron Springs, WA). Categorized as occurring during warm-water events (e.g., El Niño). There are no catalogued records collected within MBNMS; however, large fish are less commonly stored in museums.

***Thunnusorientalis*** (Temminck & Schlegel, 1844). **Pacific Bluefin Tuna**. Museum specimen: CAS-ICH 213885 (as synonym *Thunnusthynnus*; between Point Pinos and Point Joe during 1958, El Niño, WI Follett). Publications: Fast (1957) (Monterey Bay during 1956, warm-water event); Radovich (1961) (Monterey during 1957, El Niño). Categorized as occurring during warm-water events (e.g., El Niño).

##### Family XIPHIIDAE – swordfishes

***Xiphiasgladius*** Linnaeus, 1758. **Swordfish**. Publication: Radovich (1961) (Monterey and Davidson Seamount area during 1957, El Niño). Categorized as occurring during warm-water events (e.g., El Niño). There are no catalogued records collected within MBNMS; however, large fish are less commonly stored in museums.

##### Family ISTIOPHORIDAE – billfishes

***Kajikiaaudax*** (Philippi, 1887). **Striped Marlin**. Publication: Hill and Haight (1985) (32 km SW of Santa Cruz during 1983, El Niño, examined by RN Lea). Hill and Haight (1985) reported a striped marlin capture 32 km southwest of Santa Cruz by sport fisherman on 5 Sept 1983 (during an extensive El Niño event, specimen examined and identified by RN Lea). Categorized as occurring during warm-water events (e.g., El Niño). There are no catalogued records collected within MBNMS; however, large fish are less commonly stored in museums.

##### Family CENTROLOPHIDAE – medusafishes

***Icichthyslockingtoni*** Jordan and Gilbert, 1880. **Medusafish**. Museum specimens: CAS-ICH 37491 (Santa Cruz, T Iwamoto); CAS-ICH 218179 (Monterey Canyon, EH Ahlstrom); CAS-ICH 218182 (Pacific Grove, WI Follett); SWFSC uncatalogued (Davidson Seamount during May 2015, RN Lea and EJ Burton).

##### Family TETRAGONURIDAE – squaretails

***Tetragonuruscuvieri*** Risso, 1810. **Smalleye Squaretail**. Museum specimens: CAS-ICH 14272 (SW of Santa Cruz during 1959, El Niño, identifier unknown); LACM 9888.001 (off Big Sur during Oct 1967, K Mais, California Department of Fish and Game). Publications: Fitch (1949, 1951) (Davidson Seamount during 1948); Radovich (1961) (Monterey Bay during 1959, El Niño). Usually occurs in deep water, well offshore (Miller and Lea 1972). Categorized as occurring during warm-water events (e.g., El Niño).

##### Family STROMATEIDAE – butterfishes

***Peprilussimillimus*** (Ayres, 1860). **Pacific Pompano**. Museum specimens: CAS-ICH 79889 (Monterey Bay, Freihofer); MLMLF1387 (Kirby Park at Elkhorn Slough, E Yarberry); MLMLF1388 (Moss Landing Harbor, E Yarberry); UMMZ 63402 (Monterey Bay, WL Scofield).

#### Order Pleuronectiformes

##### Family PARALICHTHYIDAE – sand flounders

***Citharichthyssordidus*** (Girard, 1854). **Pacific Sanddab**. Museum specimens: CAS-ICH 238635 (Monterey Bay, RR Rofen); CAS-SU 23735 (Santa Cruz, D Villadolid); SWFSC uncatalogued (Davidson Seamount during May 2015, W Watson); UAM 730 (Elkhorn Slough, identifier unknown).

***Citharichthysstigmaeus*** Jordan and Gilbert, 1882. **Speckled Sanddab**. Museum specimens: CAS-ICH 26296 (Monterey, WI Follett); MLMLF1397 (Kirby Park at Elkhorn Slough, LT Ackerman); CAS-SU 23688 (Pacific Grove, D Villadolid); SWFSC uncatalogued (Davidson Seamount during May 2015, W Watson). Publications: Yoklavich et al. (1991) (Hwy 1 Bridge and Dairy at Elkhorn Slough); Brown (2006) (Elkhorn Slough).

***Citharichthysxanthostigma*** Gilbert, 1890. **Longfin Sanddab**. Museum specimens: CAS-ICH 228405 (Monterey Bay, R Bolin); NCSM 80806 (34, Monterey Bay, FJ Schwartz). Publication: Phillips (1967) (SSW of Point Santa Cruz).

***Hippoglossinastomata*** Eigenmann and Eigenmann, 1890. **Bigmouth Sole**. Museum specimen: SIO 05-88 (Lucia Canyon, D Kamikawa). Publication: Phillips (1965b) (off Salinas River at Monterey Bay).

***Paralichthyscalifornicus*** (Ayres, 1859). **California Halibut**. Museum specimens: MLMLF1404 (Kirby Park at Elkhorn Slough, E Yarberry); MLMLF1405 (Kirby Park at Elkhorn Slough, LT Ackerman); CAS-SU 12176 (Pacific Grove, EC Starks). Publications: Phillips (1945) (off Moss Landing); Yoklavich et al. (1991) (Hwy 1 Bridge, Kirby Park, and Hudson’s Landing at Elkhorn Slough).

***Xystreurysliolepis*** Jordan and Gilbert, 1880. **Fantail Sole**. Museum specimen: MLMLF1406 (Monterey Bay during 1987, J Brennan); Publication: Phillips (1963) (off Salinas River at Monterey Bay during Jul 1962).

##### Family PLEURONECTIDAE – righteye flounders

***Atheresthesevermanni*** Jordan and Starks, 1904. **Kamchatka Flounder**. Museum specimen and Publication: SIO 96-72 (off Davenport, Lea 2013).

***Atheresthesstomias*** (Jordan & Gilbert, 1880). **Arrowtooth Flounder**. Museum specimen: CAS-ICH 26232 (off Half Moon Bay during 1957, identifier unknown).

***Clidodermaasperrimum*** (Temminck & Schlegel, 1846). **Roughscale Sole**. Museum specimen: SIO 95-23 (west of Half Moon Bay, RN Lea). Common name follows Lea et al. (1989).

***Embassichthysbathybius*** (Gilbert, 1890). **Deepsea Sole**. Museum specimens: CAS-ICH 5617 (off Point Sur, ME Anderson); CAS-ICH 15075 (off Montara, identifier unknown); CAS-ICH 34349 (Monterey Canyon, ME Anderson); CAS-ICH 42565 (off Cypress Point, RN Lea).

***Eopsettajordani*** (Lockington, 1879). **Petrale Sole**. Museum specimens: ANSP 16325 (Pacific Grove, Harold Heath); CAS-ICH 45961 (Point Montara, E Johnston and F Sumner, US Fish Commission); USNM 77420 (off Santa Cruz Lighthouse, identifier unknown, “Albatross” Explorations on the California Coast, 1904).

***Glyptocephaluszachirus*** Lockington, 1879. **Rex Sole**. Museum specimens: CAS-ICH 48187 (Monterey Bay, S Richardson); CAS-ICH 233952 (off Point Pinos, CL Hubbs); SIO 67-102 (within Davidson Seamount Management Zone, CL Hubbs).

***Hippoglossoideselassodon*** Jordan and Gilbert, 1880. **Flathead Sole**. Museum specimens: MLMLF1420 (Monterey Bay, T Dimitre); SIO 11-32 (Monterey Bay, HC McWilliams, NMFS). Publication: Allen and Smith (1988) (survey data indicate southern boundary at Monterey).

***Hippoglossusstenolepis*** Schmidt, 1904. **Pacific Halibut**. Publications: Starks (1919) (Monterey Bay); Phillips (1935) (Monterey); Phillips (1958) (4 miles south of Point Piedras Blancas during Sept 1957, a warm-water year).

***Isopsettaisolepis*** (Lockington, 1880). **Butter Sole**. Museum specimens: CAS-ICH 27646 (Natural Bridges State Park, B Wesemann); CAS-ICH 39768 (Monterey Bay, RL Bolin).

***Lepidopsettabilineata*** (Ayres, 1855). **Rock Sole**. Museum specimens: CAS-ICH 40341 (NW of Point Pinos, T Iwamoto); MLMLF1422 (off Waddell Creek, E Yarberry); CAS-SU 3606 (off Point Piedras Blancas, identifier unknown, US Fish Commission). Publication: Gilbert (1915) (Monterey Bay).

***Lyopsettaexilis*** (Jordan & Gilbert, 1880). **Slender Sole**. Museum specimens: CAS-SU 16043 and CAS-SU 16044 (Monterey Bay, RL Bolin); CAS-SU 23698 (Monterey Bay, D Villadolid). Publication: Gilbert (1915) (Monterey Bay).

***Microstomuspacificus*** (Lockington, 1879). **Dover Sole**. Museum specimens: CAS-ICH 37533 (west of Point Año Nuevo, WN Eschmeyer); SIO 67-102 (within Davidson Seamount Management Zone, CL Hubbs, 35 mm); CAS-SU 18680 (off Monterey Bay, RL Bolin); CAS-SU 25653 (Santa Cruz, EC Starks).

***Parophrysvetulus*** Girard, 1854. **English Sole**. Museum specimens: CAS-ICH 26233 (off Point Montara, identifier unknown); CAS-SU 16047 (Monterey Bay, RL Bolin); CAS-SU 58452 (Elkhorn Slough, JC Briggs). Publications: Gilbert (1915) (Monterey Bay); Yoklavich et al. (1991) (Hwy 1 Bridge, Dairy, Kirby Park, and Hudson’s Landing at Elkhorn Slough); Brown (2006) (Elkhorn Slough).

***Platichthysstellatus*** (Pallas, 1787). **Starry Flounder**. Museum specimens: CAS-SU 12166 (Pacific Grove, RL Bolin); CAS-SU 16428 (Monterey Bay, RL Bolin); CAS-SU 34279 (Elkhorn Slough, WI Follett). Publication: Yoklavich et al (1991) (Hwy 1 Bridge, Dairy, Kirby Park, and Hudson’s Landing at Elkhorn Slough).

***Pleuronichthyscoenosus*** Girard, 1854. **C-O Sole**. Museum specimen: CAS-SU 58398 (Monterey Bay, JC Briggs).

***Pleuronichthysdecurrens*** Jordan and Gilbert, 1881. **Curlfin Turbot**. Museum specimens: CAS-ICH 26081 (Half Moon Bay, identifier unknown); CAS-SU 3605 (offshore north of Cambria, identifier unknown, US Fish Commission); CAS-SU 51270 (Monterey Bay, WC Freihofer). Publication: Yoklavich et al. (1991) (Hwy 1 Bridge and Dairy at Elkhorn Slough).

***Pleuronichthysguttulatus*** Girard, 1856. **Diamond Turbot**. Museum specimens: CAS-ICH 23877 (Sea Cliff State Park, WI Follett); CAS-SU 4426 (Monterey, identifier unknown); CAS-SU 58381 (Elkhorn Slough, JC Briggs). Publication: Yoklavich et al. (1991) (Dairy, Kirby Park, and Hudson’s Landing at Elkhorn Slough).

***Pleuronichthysverticalis*** Jordan and Gilbert, 1880. **Hornyhead Turbot**. Museum specimens: KU 1620 (Monterey, RL Bolin); SIO 85-155 (Pigeon Point, D Gibson); UF 79823 (Monterey Bay, G Burgess).

***Psettichthysmelanostictus*** Girard, 1854. **Sand Sole**. Museum specimens: MLMLF1445 (Moss Landing Harbor mouth, LT Ackerman); CAS-SU 4379 (Monterey, DS Jordan); CAS-SU 12164 (Pacific Grove, EC Starks); CAS-SU 58471 (Carmel Beach: JC Briggs). Publication: Gilbert (1915) (Monterey Bay).

##### Family CYNOGLOSSIDAE – tonguefishes

***Symphurusatricaudus*** (Jordan & Gilbert, 1880). **California Tonguefish**. Museum specimens: CAS-ICH 24050 (Monterey Bay, RR Harry); MLMLF1449 (Kirby Park at Elkhorn Slough, E Yarberry). Publications: Phillips (1942b) (Monterey area during 1942); Yoklavich et al. (1991) (Hwy 1 Bridge and Hudson’s Landing at Elkhorn Slough).

#### Order Tetraodontiformes

##### Family BALISTIDAE – triggerfishes

***Balistespolylepis*** Steindachner, 1876. **Finescale Triggerfish**. Museum specimens: CAS-ICH 33303 (off Manresa State Beach during 1975, ME Anderson); CAS-ICH 50060 (off Hopkins Marine Station during 1982, D Powell); CAS-ICH 52482 (outside Pillar Point Harbor during 1983, El Niño, L Dempster); CAS-SU 18100 (Monterey Harbor during Jun 1951, RL Bolin). Publications: Bolin (1952) (Monterey Harbor during Jun 1951); Radovich (1961) (Monterey Bay during 1958, El Niño). Categorized as occurring during warm-water events (e.g., El Niño).

##### Family DIODONTIDAE – porcupinefishes

***Diodonholocanthus*** Linnaeus, 1758. **Balloonfish**. Museum specimen: CAS-SU 52706 (San Gregorio Beach during 1958, El Niño, JM Leis). Typically occurs in warm seas; rare off California (Miller and Lea 1972). Categorized as occurring during warm-water events (e.g., El Niño).

##### Family MOLIDAE – molas

***Molamola*** (Linnaeus, 1758). **Ocean Sunfish**. Museum specimens: LACM 55986.001 (Monterey, J O'Sullivan); MLMLF1457 (Monterey Bay, ME Anderson). Publication: Gotshall (1961) (Monterey Bay).

#### Extralimital species

The geographic ranges for the following 18 species encompass MBNMS boundaries. They are likely to occur within MBNMS; however, no verifiable records occur from within MBNMS. Until further evidence is found, we consider these extralimital species.

##### Order Rajiformes

###### Family ARHYNCHOBATIDAE – softnose skates

***Bathyrajamicrotrachys*** (Osburn & Nichols, 1916). **Fine-spined Skate**. Known to occur from Washington to “southern California” (Last et al. 2016d). Records occur to the north (Farallon Islands, CAS) and south (Guadalupe Island, Baja California, holotype, USNM). Common name follows Ebert (2003).

***Bathyrajaspinosissima*** (Beebe and Tee-Van, 1941). **Pacific White Skate**. Known to occur from Oregon to Ecuador (Last et al. 2016d). Records occur to the north (off Oregon, CAS), west (Gumdrop Seamount, CAS), and south (Ecuador). Common name follows Ebert (2003).

##### Order Stomiiformes

###### Family STERNOPTYCHIDAE – marine hatchetfishes

***Sternoptyxpseudobscura*** Baird, 1971. **Highlight Hatchetfish**. Records occur to the north (British Columbia: Peden 1974, Milkova et al. 2016) and south (southern California, SIO).

###### Family STOMIIDAE – dragonfishes

***Opostomiasmitsuii*** Imai, 1941. **Pitgum Dragonfish**. Records occur to the west, north, and south (USNM, UW, Peden 1974, Milkova et al. 2016). Ranges across the temperate north Pacific; rare in the CalCOFI sampling area (Moser 1996).

##### Order Myctophiformes

###### Family NEOSCOPELIDAE – blackchins

***Scopelengystristis*** Alcock, 1890. **Pacific Blackchin**. Records occur to the north (British Columbia, Milkova et al. 2016; Oregon, UW records) and south (southern California, SIO).

###### Family MYCTOPHIDAE – lanternfishes

***Lampadenaurophaos*** Paxton, 1963. **Sunbeam Lampfish**. Type locality is southern California. Occurs between 25° N and 42° N in the central and eastern Pacific (Nafpaktitis and Paxton 1968).

##### Order Scorpaeniformes

###### Family SCORPAENIDAE – scorpionfishes

***Sebastesalutus*** (Gilbert, 1890). **Pacific Ocean Perch**. Literature indicates the species occurs from central Baja to Bering Sea and Japan (Miller and Lea 1972, Love et al. 2002, Butler et al. 2012). Records nearest to MBNMS occur off Point Arena (north) and SE of Santa Cruz Island (south).

###### Family COTTIDAE – sculpins

***Paricelinushopliticus*** Eigenmann and Eigenmann, 1889. **Thornback Sculpin**. Records occur to the north (Cordell Bank, CAS; and Farallon Islands, SIO) and south (Point Loma, SIO). Considered a rare fish, occurring in deep, rocky bottom habitats.

##### Order Perciformes

###### Family ZOARCIDAE – eelpouts

***Lycodapusendemoscotus*** Peden and Anderson, 1978. **Deepwater Eelpout**. Known from lower continental slope waters between British Columbia to northern Mexico and the Gulf of California in depths from 933 to 2,225 m (Peden and Anderson 1978). Those habitats are found in MBNMS; and based on published range, is likely to occur in MBNMS (personal communication, ME Anderson). Common name follows Mecklenburg et al. (2002).

***Pachycaragymninium*** Anderson and Peden, 1988. **Nakednape Eelpout**. Known from off Queen Charlotte Islands, British Columbia to off Guadalupe Island, Mexico and in the Gulf of California in depths from 1,829 to 3,219 m over brown and green mud bottoms (Anderson and Peden 1988). Those habitats are found in MBNMS; and based on published range, is likely to occur in MBNMS (pers. comm. ME Anderson). Records off California include Mendocino Ridge (3,225 m) and off Cordell Bank (2,707-3,219 m). Common name follows Mecklenburg et al. (2002).

***Pachycaralepinium*** Anderson and Peden, 1988. **Scalynape Eelpout**. Known from off Queen Charlotte Islands, British Columbia to off Guadalupe Island, Mexico in depths from 1,728 to 2,970 m over brown and green mud bottoms (Anderson and Peden 1988). Those habitats are found in MBNMS; and based on published range, is likely to occur in MBNMS (pers. comm. ME Anderson). Records off California include off Cape Mendocino (2,940 m), off Farallon Islands (1,800 m), and San Clemente Basin (1,829-2,027 m). Common name follows Mecklenburg et al. (2002).

###### Family STICHAEIDAE – pricklebacks

***Esselenichthyslaurae*** (Follett & Anderson, 1990). **Twoline Prickleback**. Follett and Anderson (1990) described the species from 15 juvenile specimens captured between Southeast Farallon Island, California, and Punta Banda, Baja California Norte, Mexico. The Southeast Farallon Island specimens (CAS) were collected from pigeon guillemot nests, but their condition indicates they were taken locally (Follett and Anderson 1990).

***Poroclinusrothrocki*** Bean, 1890. **Whitebarred Prickleback**. Northeast Pacific records include Alaska, Washington, Oregon, northern California (Crescent City, Eureka, Fort Bragg; LACM), and one record in San Diego (CAS-SU).

###### Family GOBIIDAE – gobies

***Ilypnusgilberti*** (Eigenmann & Eigenmann, 1889). **Cheekspot Goby**. Occurs in mud flats of bays (Miller and Lea 1972); considered an estuarine species. No records from Elkhorn Slough; however, found to the north and south of MBNMS (e.g., Tomales Bay, San Francisco Bay, southern California).

###### Family GEMPYLIDAE – snake mackerels

***Lepidocybiumflavobrunneum*** (Smith, 1843). **Escolar**. Although range occurs from Peru to Washington (Miller and Lea 1972), it is rare, found deep, and is a more tropical species.

###### Family ISTIOPHORIDAE – billfishes

***Tetrapturusangustirostris*** Tanaka, 1915. **Shortbill Spearfish**. Found in eastern Pacific from Chile to 40 miles west of Cape Mendocino (Miller and Lea 1972). Radovich 1961 reported a sport-catch record off Morro Bay in Aug 1959 (El Niño). In addition, two collected outside of MBNMS: 100 miles offshore, halfway between San Francisco and Morro Bay (SIO, Jul 1959); and ~120 miles west of Monterey (CAS, K Peterson, Sept 1981).

##### Order Pleuronectiformes

###### Family PLEURONECTIDAE – righteye flounders

***Reinhardtiushippoglossoides*** (Walbaum, 1792). **Greenland Halibut**. Found in the northern part of the Pacific, from Sagami Bay northward, in the Sea of Japan, the Okhotsk Sea, the Bering Sea, and off the Pacific coast of North America south to Mexico (Dyck et al. 2007). It is uncommon off California (Miller and Lea 1972); several records off Eureka. The Mexican record (SIO) was captured during 1962; a cold-water year.

##### Order Tetradontiformes

###### Family TETRAODONTIDAE – puffers

***Lagocephaluslagocephalus*** (Linnaeus, 1758). **Oceanic Puffer**. Published range in eastern Pacific is Galapagos Islands to Alder Creek Beach, Mendocino County; rare off California (Miller and Lea 1972). Five California museum specimens exist (4 at southern CA, 1 at Mendocino during 1958 El Niño, CAS). Typically occurs in warmer seas; however, can move north during El Niño years.

## References

[B1] Ahlstrom EH Moser HG Cohen DM (1984) Argentinoidei: Development and relationships. In: Moser HG Richards WJ Cohen DM Fahay MP Kendall AW Jr Richardson SL (Eds) Ontogeny and Systematics of Fishes.American Society of Ichthyologists and Herpetologists, Special Publication No 1: 155–169. http://swfsc.noaa.gov/publications/CR/1984/8402.PDF

[B2] Allen MJ Smith GB (1988) Atlas and zoogeography of common fishes in the Bering Sea and northeastern Pacific. US Department of Commerce, NOAA Technical Report NMFS 66, 151 pp. https://spo.nmfs.noaa.gov/sites/default/files/legacy-pdfs/tr66.pdf

[B3] Anderson ME (1995) The eelpout genera *Lycenchelys* Gill and *Taranetzella* Andriashev (Teleostei: Zoarcidae) in the eastern Pacific, with descriptions of nine new species. Proceedings of the California Academy of Sciences (Series 4) 49(2): 55–113. https://www.biodiversitylibrary.org/part/52965

[B4] Anderson ME (2012) A new species of *Pachycara* Zugmayer (Teleostei: Zoarcidae) from off Monterey Bay, California, USA, with comments on two North Pacific *Lycenchelys* species. Zootaxa 3559: 39–43. 10.11646/zootaxa.3559.1.3

[B5] Anderson ME Peden AE (1988) The eelpout genus *Pachycara* (Teleostei: Zoarcidae) in the northeastern Pacific Ocean, with descriptions of two new species. Proceedings of the California Academy of Sciences (Series 4) 46(3): 83–94. https://www.biodiversitylibrary.org/part/53679

[B6] Anderson ME Cailliet GM Antrim BS (1979) Notes on some uncommon deep-sea fishes from the Monterey Bay Area, California. California Fish and Game 65(4): 256–264. https://www.biodiversitylibrary.org/item/61897

[B7] Anderson ME Fedorov VV (2004) Family Zoarcidae Swainson 1839 eelpouts. California Academy of Sciences Annotated Checklists of Fishes 34: 1–58. https://www.calacademy.org/sites/default/files/assets/docs/zoarcidae.pdf

[B8] Andrews CB (1927) Albacore caught near Monterey. California Fish and Game 13(2): 141–142. https://www.biodiversitylibrary.org/item/70558

[B9] Ayres WO (1854) Description of new fishes from California. (Minutes of Academy meetings were printed in "The Pacific" (a newspaper) shortly after each meeting. New species date to publication in The Pacific. Dates of publication are given in each species account). The Pacific [newspaper] vol. 3 and 4 (thru no. 6). [Also as Proceedings of the California Academy of Natural Sciences vol. 1 (nos. 3-22).] https://www.biodiversitylibrary.org/item/54191

[B10] Ayres WO (1860a) Descriptions of new species of fishes. Proceedings of the California Academy of Sciences (Series 1) 2: 60–64. http://www.biodiversitylibrary.org/page/3836984

[B11] Ayres WO (1860b) [Description of Fishes]. Proceedings of the California Academy of Sciences (Series 1) 2: 77–86. http://www.biodiversitylibrary.org/page/3837001

[B12] Balanov A Savinykh VF (1999) Redescription of *Scopelosaurusharryi* and *S.adleri* (Notosudidae): two valid mesopelagic species inhabiting the northern part of the Pacific Ocean. Journal of Ichthyology 39(8): 616–625.

[B13] Balushkin AV (2012) *Volodichthys* gen. nov. new species of the primitive snailfish (Liparidae: Scorpaeniformes) of the Southern Hemisphere. Description of new species *V.solovjevae* sp. nov. (Cooperation Sea, the Antarctic). Journal of Ichthyology 52(1): 1–10. 10.1134/S0032945212010018

[B14] Barry JP (1983) Utilization of shallow marsh habitats by fishes in Elkhorn Slough, California. MSc Thesis, San Jose State University, California. http://islandora.mlml.calstate.edu/islandora/object/islandora%3A461

[B15] Bean TH (1894) Description of a new blennioid fish from California. Proceedings of the United States National Museum 16(967): 699–701. 10.5479/si.00963801.16-967.699

[B16] Bolin RL (1938) *Bathylaguswesethi*, a new Argentinid fish from California. California Fish and Game 24(1): 66–68. https://www.biodiversitylibrary.org/item/70695

[B17] Bolin RL (1939) A new stomiatoid fish from California. Copeia 1939(1): 39–41. 10.2307/1436014

[B18] Bolin RL (1952) Two unusual records of marine fishes at Monterey, California. California Fish and Game 38(2): 209–210. https://www.biodiversitylibrary.org/item/61382

[B19] Boulenger GA (1895) Catalogue of the fishes in the British Museum. Catalogue of the perciform fishes in the British Museum. Second edition. Vol. I. Catalogue of the fishes in the British Museum (2^nd^ edn) Vol. 1, 394 pp. [pls. 1–15] https://babel.hathitrust.org/cgi/pt?id=uc1.31822032980716;view=1up;seq=5

[B20] Bradbury MG Cohen DM (1958) An illustration and a new record of the North Pacific bathypelagic fish *Macropinnamicrostoma*. Stanford Ichthyological Bulletin 7(3): 57–59.

[B21] Brown JA (2006) Classification of juvenile flatfishes to estuarine and coastal habitats based on the elemental composition of otoliths. Estuarine, Coastal and Shelf Science 66: 594–611. 10.1016/j.ecss.2005.11.005

[B22] Burton EJ Kuhnz LA DeVogelaere AP Barry JP (2017) Sur Ridge Field Guide: Monterey Bay National Marine Sanctuary. Marine Sanctuaries Conservation Series ONMS-17-10. US Department of Commerce, National Oceanic and Atmospheric Administration, Office of National Marine Sanctuaries, Silver Spring, 122 pp. https://sanctuaries.noaa.gov/science/conservation/sur-ridge-field-guide-monterey-bay-national-mairne-sanctuary.html

[B23] Burton EJ Lundsten L (2008) Davidson Seamount Taxonomic Guide. Marine Sanctuaries Conservation Series ONMS-08-08. United States Department of Commerce, National Oceanic and Atmospheric Administration, Office of National Marine Sanctuaries, Silver Spring, 145 pp. Available at: https://sanctuaries.noaa.gov/science/conservation/pdfs/taxonomic.pdf

[B24] Butler JL Love MS Laidig TE (2012) A guide to the rockfishes, thornyheads, and scorpionfishes of the Northeast Pacific. University of California Press, Berkeley, 185 pp.

[B25] Caffrey JM Broenkow WW (2002) Hydrography. In: Caffrey JM Brown M Tyler WB (Eds) Changes in a California Estuary: An Ecosystem Profile of Elkhorn Slough.Monterey Bay Aquarium Foundation. Monterey, California, 29–42. http://digital.mlml.calstate.edu/islandora/object/islandora%3A2447

[B26] Cailliet GM Anderson ME (1975) Occurrence of the prowfish *Zaprorasilenus* Jordan, 1896 in Monterey Bay, California. California Fish and Game 61(1): 60–62. https://www.biodiversitylibrary.org/item/61825

[B27] Cailliet GM Lea RN (1977) Abundance of the “rare” zoarcid, *Mayneacalifornica* Gilbert, 1915, in the Monterey Canyon, Monterey Bay, California. California Fish and Game 63(4): 253–261. https://www.biodiversitylibrary.org/item/61890

[B28] Cailliet GM Antrim B Ambrose D Pace S Stevenson M (1977) Species composition, abundance and ecological studies of fishes, larval fishes, and zooplankton in Elkhorn Slough. In: Nybakken JW Cailliet GM Broenkow WW (Eds) Ecological and hydrographic studies of Elkhorn Slough, Moss Landing Harbor and nearshore coastal waters, July 1974 to June 1976.Moss Landing Marine Laboratories, Moss Landing, California, 216–386. http://aquaticcommons.org/2607/

[B29] Carlisle AB Litvin SY Hazen EL Madigan DJ Goldman KJ Lea RN Block BA (2015) Reconstructing habitat use by juvenile salmon sharks links upwelling to strandings in the California Current. Marine Ecology Progress Series 525: 217–228. 10.3354/meps11183

[B30] Carvalho MR de Last PR Séret B (2016) Torpedo Rays. Family Torpedinidae. In: Last PR White WT Carvalho MR de Séret B Stehmann MFW Naylor GJP (Eds) Rays of the World.Cornell University Press, Ithaca, New York, 184–203. http://www.publish.csiro.au/book/7053/

[B31] Castro JI (2011) The Sharks of North America. Oxford University Press, New York, 613 pp.

[B32] CDFW [California Department of Fish and Wildlife] (2017) Fishing and Diving Records. https://www.wildlife.ca.gov/Fishing/Records [accessed 9 Feb 2017]

[B33] Cohen DM Inada T Iwamoto T Scialabba N (1990) Gadiform Fishes of the World (Order Gadiformes). An annotated and illustrated catalogue of cods, hakes, grenadiers and other gadiform fishes known to date. FAO Fisheries Synopsis 125(10): 1–442.

[B34] Collette BB Aaland CR (1996) Revision of the frigate tunas (Scombridae, *Auxis*), with descriptions of two new subspecies from the eastern Pacific. Fishery Bulletin 94(3): 423–441. https://www.st.nmfs.noaa.gov/spo/FishBull/943/collette.pdf

[B35] Compagno LJV (1988) Sharks of the Order Carcharhiniformes. Princeton University Press, Princeton, New Jersey, 572 pp.

[B36] Cooper JG (1863) On new genera and species of Californian fishes - No. II. Proceedings of the California Academy of Sciences (Series 1) 3: 93–97. http://www.biodiversitylibrary.org/page/3148002

[B37] Cox KW (1948) Sablefish run at Monterey Bay. California Fish and Game 34(1): 37. https://www.biodiversitylibrary.org/item/70838

[B38] Cramer F (1895) On the cranial characters of the genus *Sebastodes* (rock-fish). Proceedings of the California Academy of Sciences (Series 2) 5: 573–610. http://www.biodiversitylibrary.org/page/32283261

[B39] Didier DA Séret B (2002) Chimaeroid fishes of New Caledonia with description of a new species of *Hydrolagus* (Chondrichthyes, Holocephali). Cybium 26(3): 225–233.

[B40] Dill WA Cordone AJ (1997) History and status of introduced fishes in California, 1871-1996. California Department of Fish and Game, Fish Bulletin 178: 1–414. https://escholarship.org/uc/item/5rm0h8qg

[B41] Dyck M Warkentin PH Treble MA (2007) A bibliography on the Greenland halibut, *Reinhardtiushippoglossoides* (a.k.a. Greenland turbot) 19;6-2005. Canadian Technical Report of Fisheries and Aquatic Sciences 2683, 309 pp. http://publications.gc.ca/site/eng/422643/publication.html

[B42] Ebeling AW (1962) Melamphaidae I. Systematics and zoogeography of the species in the bathypelagic fish genus *Melamphaes* Günther. Dana-Report 58(1): 1–164.

[B43] Ebert DA (2003) The Sharks, Rays and Chimaeras of California. University of California Press, Berkeley, 284 pp. https://www.ucpress.edu/book.php?isbn=9780520234840

[B44] Ebert DA Mollet HF Baldridge A Thomas T Forney KA Ripley WE (2004) Occurrence of the whale shark, *Rhincodontypus* Smith 1828, in California waters. Northwestern Naturalist 85(1): 26–28. 10.1898/1051-1733(2004)085<0026:OOTWSR>2.0.CO;2

[B45] Eigenmann CH Beeson CH (1894) *Pteropodusdallii* sp. nov. American Naturalist 28(325): 66. 10.1086/275871

[B46] Eschmeyer WN Fricke R van der Laan R (2017) Catalog of Fishes: Genera, Species, References. http://researcharchive.calacademy.org/research/ichthyology/catalog/fishcatmain.asp [accessed 31 May 2017]

[B47] Eschmeyer WN Herald ES Hammann H (1983) A Field Guide to Pacific Coast Fishes. Houghton Mifflin Company, Boston, 336 pp.

[B48] Fast TN (1957) The occurrence of the deep-sea anglerfish, *Cryptopsarascouesii*, in Monterey Bay, California. Copeia 1957(3): 237–240. 10.2307/1439375

[B49] Fitch JE (1949) Some unusual occurrences of fish on the Pacific coast. California Fish and Game 35(1): 41–49. https://www.biodiversitylibrary.org/item/61373

[B50] Fitch JE (1951) Notes on the squaretail, *Tetragonuruscuvieri*. California Fish and Game 37(1): 55–59. https://www.biodiversitylibrary.org/item/61378

[B51] Fitch JE (1963) A review of the fishes of the genus *Pleuronichthys*. Los Angeles County Museum Contributions in Science No. 76: 33 pp. https://www.biodiversitylibrary.org/part/241024

[B52] Fitch JE (1964) *Sebastodesphillipsi*, a new scorpaenid fish from Californian waters. Copeia 1964(3): 525–529. 10.2307/1441517

[B53] Fitch JE Lavenberg RJ (1968) Deep-Water Fishes of California. University of California Press, Berkeley, 155 pp. https://archive.org/details/deepwaterteleost00fitc

[B54] Follett WI (1948) A northerly record of *Polydactylusapproximans* (Lay and Bennett), a polynemid fish of the Pacific Coast of tropical America. Copeia 1948(1): 34–40. 10.2307/1438788

[B55] Follett WI (1966) Man-eater of the California coast. Pacific Discovery 19(1): 18–22.

[B56] Follett WI Anderson ME (1990) *Esselenia*, a new genus of pricklebacks (Teleostei: Stichaeidae), with two new species from California and Baja California Norte. Copeia 1990(1): 147–163. 10.2307/1445831

[B57] Follett WI Powell DC (1988) *Ernogrammuswalkeri*, a new species of prickleback (Pisces: Stichaeidae) from south-central California. Copeia 1988(1): 135–152. 10.2307/1445933

[B58] Frable BW Wagman DW Frierson TN Aguilar A Sidlauskas BL (2015) A new species of *Sebastes* (Scorpaeniformes: Sebastidae) from the northeastern Pacific, with a redescription of the blue rockfish, *S.mystinus* (Jordan and Gilbert, 1881). Fishery Bulletin 113(4): 355–377. 10.7755/FB.113.4.1

[B59] Frey HW (1962) A range extension for the Mexican scad to Monterey Bay, California. California Fish and Game 48(3): 210–211. https://www.biodiversitylibrary.org/item/63636

[B60] Gaither MR Violi B Gray HWI Neat F Drazen JC Grubbs RD Roa-Varón A Sutton T Hoelzel AR (2016) Depth as a driver of evolution in the deep sea: Insights from grenadiers (Gadiformes: Macrouridae) of the genus Coryphaenoides. Molecular Phylogenetics and Evolution 104: 73–82. 10.1016/j.ympev.2016.07.027 27475496

[B61] Gibbs MA (1991) Notes on the distribution and morphology of the rubynose brotula (*Cataetyxrubrirostris*) off central California. California Fish and Game 77(3): 149–152. https://www.biodiversitylibrary.org/item/72536

[B62] Gilbert CH (1896) The ichthyological collections of the steamer Albatross during the years 1890 and 1891. United States Commission of Fish and Fisheries, Report of the Commissioner 19(1893): 393–476. https://babel.hathitrust.org/cgi/pt?id=hvd.hwfibi;view=1up;seq=9

[B63] Gilbert CH (1904) Notes on fishes from the Pacific coast of North America. Proceedings of the California Academy of Sciences (Series 3) 3(9): 255–271. http://www.biodiversitylibrary.org/page/31548105

[B64] Gilbert CH (1914) Two cottoid fishes from Monterey Bay, California. Proceedings of the United States National Museum 47(2049): 135–137. 10.5479/si.00963801.47-2049.135

[B65] Gilbert CH (1915) Fishes collected by the United States Fisheries steamer "Albatross" in southern California in 1904. Proceedings of the United States National Museum 48(2075): 305–380. 10.5479/si.00963801.48-2075.305

[B66] Girard CF (1854) Observations upon a collection of fishes made on the Pacific coast of the United States, by Lieut. W.P. Trowbridge, U.S.A., for the museum of the Smithsonian Institution. Proceedings of the Academy of Natural Sciences of Philadelphia 7: 142–156. https://repository.si.edu/handle/10088/34410

[B67] Girard CF (1855) Characteristics of some cartilaginous fishes of the Pacific coast of North America. Proceedings of the Academy of Natural Sciences of Philadelphia 7(6): 196–197. https://www.biodiversitylibrary.org/part/7975

[B68] Girard CF (1858a) Fishes. In: Part IV: General report upon zoology of the several Pacific railroad routes, 1857. In: Reports of explorations and surveys, to ascertain the most practicable and economical route for a railroad from the Mississippi River to the Pacific Ocean, v. 10. Beverley Tucker, Washington, DC, 400 pp. https://www.biodiversitylibrary.org/item/57065

[B69] Girard CF (1858b) Notice upon new genera and new species of marine and fresh-water fishes from western North America. Proceedings of the Academy of Natural Sciences of Philadelphia 9(15): 200–202. https://www.biodiversitylibrary.org/item/30013

[B70] Gotshall DW (1961) Observations on a die-off of molas (*Molamola*) in Monterey Bay. California Fish and Game 47(4): 339–341. https://www.biodiversitylibrary.org/item/61609

[B71] Greeley AW (1898) *Oligocottussnyderi.* In: Jordan DS, Evermann BW (Eds) The fishes of North and Middle America: a descriptive catalogue of the species of fish-like vertebrates found in the waters of North America north of the Isthmus of Panama. Part III. Bulletin of the United States National Museum No. 47: 2871. https://www.biodiversitylibrary.org/item/32368

[B72] Greeley AW (1899) Notes on the tide-pool fishes of California, with a description of four new species. Bulletin of the United States Fish Commission 19: 7–20. https://www.biodiversitylibrary.org/item/211768

[B73] Guerrero J Kvitek RG (1996) Monterey Bay National Marine Sanctuary Site Characterization. https://montereybay.noaa.gov/sitechar/welcome.html

[B74] Günther A (1861) A preliminary synopsis of the labroid genera. Annals and Magazine of Natural History (Series 3) 8(47): 382–389. 10.1080/00222936108697435

[B75] Herald ES (1953) The 1952 shark derbies at Elkhorn Slough, Monterey Bay, and at Coyote Point, San Francisco Bay. California Fish and Game 39(2): 237–243. https://www.biodiversitylibrary.org/item/61449

[B76] Herald ES Dempster RP (1952) The 1951 shark derby at Elkhorn Slough, California. California Fish and Game 38(1): 133–134. https://www.biodiversitylibrary.org/item/61381

[B77] Hill KT Haight DR (1985) Northward range extension for the striped marlin. California Fish and Game 71(3): 185–187. https://www.biodiversitylibrary.org/item/63729

[B78] Hoff GR (1999) Range extensions of 3 species of macrourids from the west coast of North America. California Fish and Game 85(3): 113–117.

[B79] Hoff GR Buckley TW Drazen JC Duncan KM (2000) Biology and ecology of *Nezumialiolepis* and *N.stelgidolepis* from the west coast of North America. Journal of Fish Biology 57(3): 662–680. 10.1111/j.1095-8649.2000.tb00267.x

[B80] Hopkirk JD (1965) Records of yellow and spotted snake-eels (Genus *Ophichthus*) from San Francisco Bay, California. California Fish and Game 51(3): 183–186.

[B81] Horn MH Allen LG Lea RN (2006) Biogeography. In: Allen LG Pondella DJ II Horn MH (Eds) The Ecology of Marine Fishes: California and Adjacent Waters.University of California Press, Berkeley, California, 3–25. 10.1525/california/9780520246539.003.0001

[B82] Hubbs CL (1926) Notes on the gobioid fishes of California, with descriptions of two new genera. Occasional Papers of the Museum of Zoology University of Michigan 169: 1–6. https://deepblue.lib.umich.edu/handle/2027.42/56608

[B83] Hubbs CL (1927) Notes on the blennioid fishes of western North America. Papers of the Michigan Academy of Science Arts and Letters 7(1926): 351–394. https://archive.org/details/in.ernet.dli.2015.26778

[B84] Hubbs CL (1953) Revision and systematic position of the blenniid fishes of the genus *Neoclinus*. Copeia 1953(1): 11–23. 10.2307/1440237

[B85] Hubbs CL Follett WI Dempster LJ (1979) List of the fishes of California. Occasional Papers of the California Academy of Sciences, San Francisco, California 133: 1–51. https://www.biodiversitylibrary.org/item/35725

[B86] Humphreys RL Jr Winans GA Tagami DT (1989) Synonomy and life history of the North Pacific Pelagic Armorhead, *Pseudopentaceroswheeleri* Hardy (Pisces: Pentacerotidae). Copeia 1989(1): 142–153. 10.2307/1445615

[B87] Hyde JR Underkoffler KE Sundberg MA (2014) DNA barcoding provides support for a cryptic species complex within the globally distributed and fishery important opah (*Lamprisguttatus*). Molecular Ecology Resources 14: 1239–1247. 10.1111/1755-0998.12268 24751335

[B88] Iglesias SP Lecointre G Sellos DY (2005) Extensive paraphylies within sharks of the order Carcharhiniformes inferred from nuclear and mitochondrial genes. Molecular Phylogenetics and Evolution 34: 569–583. 10.1016/j.ympev.2004.10.022 15683930

[B89] Jacobson Stout N Kuhnz L Lundsten L Schlining B Schlining K von Thun S (Eds) (2017) The Deep-Sea Guide. Monterey Bay Aquarium Research Institute. http://dsg.mbari.org [accessed 8 Jun 2017]

[B90] Jordan DS (1887a) The Fisheries of the Pacific Coast. United States Commission of Fish and Fisheries, The Fisheries and Fishery Industries of the United States, Section II, Part XVI, 589–629. https://www.biodiversitylibrary.org/item/77475

[B91] Jordan DS (1887b) Coast of California. In: The Whale Fishery. 1. History and present condition of the fishery. United States Commission of Fish and Fisheries, The Fisheries and Fishery Industries of the United States, Section V, Volume II, Part XV, 52–61. https://www.biodiversitylibrary.org/item/77552

[B92] Jordan DS (1896) Notes on fishes, little known or new to science. Proceedings of the California Academy of Sciences (Series 2) 6: 201–244. http://www.biodiversitylibrary.org/page/16072474

[B93] Jordan DS Eigenmann CH (1890) A review of the genera and species of Serranidae found in the waters of America and Europe. Bulletin of the United States Fish Commission 8(1888): 329–441. https://hdl.handle.net/2027/inu.30000112165737

[B94] Jordan DS Evermann BW (1896a) A check-list of the fishes and fish-like vertebrates of North and Middle America. United States Commission of Fish and Fisheries, Report of the Commissioner, vol. 21 (for 1895), Appendix 5: 207–584. https://archive.org/details/achecklistfishe00evergoog

[B95] Jordan DS Evermann BW (1896b) The fishes of North and Middle America: a descriptive catalogue of the species of fish-like vertebrates found in the waters of North America, north of the Isthmus of Panama. Part I. Bulletin of the United States National Museum No. 47, 1240 pp. https://www.biodiversitylibrary.org/item/32405

[B96] Jordan DS Evermann BW (1900) The fishes of North and Middle America: a descriptive catalogue of the species of fish-like vertebrates found in the waters of North America, north of the Isthmus of Panama. Part IV. Bulletin of the United States National Museum No. 47, 3137–3313. [pls. 1–392] 10.5962/bhl.title.39720

[B97] Jordan DS Gilbert CH (1880a) Notes on sharks from the coast of California. Proceedings of the United States National Museum 3(118): 51–52. 10.5479/si.00963801.3-118.51

[B98] Jordan DS Gilbert CH (1880b) Description of a new species of *Sebastichthys* (*Sebastichthysminiatus*), from Monterey Bay, California. Proceedings of the United States National Museum 3(125): 70–72. 10.5479/si.00963801.3-125.70

[B99] Jordan DS Gilbert CH (1880c) Description of a new species of "rock-fish" (*Sebastichthyscarnatus*), from the coast of California. Proceedings of the United States National Museum 3(126): 73–75. 10.5479/si.00963801.3-126.73

[B100] Jordan DS Gilbert CH (1880d) Description of a new species of ray (*Raiastellulata*) from Monterey, California. Proceedings of the United States National Museum 3(129): 133–135. 10.5479/si.00963801.3-129.133

[B101] Jordan DS Gilbert CH (1880e) Descriptions of new species of *Xiphister* and *Apodichthys*, from Monterey, California. Proceedings of the United States National Museum 3(130): 135–140. 10.5479/si.00963801.3-130.135

[B102] Jordan DS Gilbert CH (1880f) Description of two new species of *Sebastichthys* (*Sebastichthysentomelas* and *Sebastichthysrhodochloris*), from Monterey Bay, California. Proceedings of the United States National Museum 3(132): 142–146. 10.5479/si.00963801.3-132.142

[B103] Jordan DS Gilbert CH (1880g) Description of a new agonoid fish (*Brachyopsisxyosternus*), from Monterey Bay, California. Proceedings of the United States National Museum 3(135): 152–154. 10.5479/si.00963801.3-135.152

[B104] Jordan DS Gilbert CH (1880h) Description of a new species of ray, *Raiarhina*, from the coast of California. Proceedings of the United States National Museum 3(141): 251–253. 10.5479/si.00963801.3-141.251

[B105] Jordan DS Gilbert CH (1880i) Description of seven new species of sebastoid fishes, from the coast of California. Proceedings of the United States National Museum 3(150): 287–298. 10.5479/si.00963801.3-150.287

[B106] Jordan DS Gilbert CH (1880j) Description of a new embiotocoid (*Abeonaaurora*), from Monterey, California, with notes on a related species. Proceedings of the United States National Museum 3(151): 299–301. 10.5479/si.00963801.3-151.299

[B107] Jordan DS Gilbert CH (1880k) Description of a new embiotocoid fish (*Ditremaatripes*), from the coast of California. Proceedings of the United States National Museum 3(156): 320–322. 10.5479/si.00963801.3-156.320

[B108] Jordan DS Gilbert CH (1880l) Description of a new scorpaenoid fish (*Sebastichthysproriger*), from Monterey Bay, California. Proceedings of the United States National Museum 3(161): 327–329. 10.5479/si.00963801.3-161.327

[B109] Jordan DS Gilbert CH (1881) Description of a new species of "rock-fish" (*Sebastichthyschrysomelas*), from the coast of California. Proceedings of the United States National Museum 3(176): 465–466. 10.5479/si.00963801.3-176.465

[B110] Jordan DS Gilbert CH (1882) Notes of the fishes of the Pacific Coast of the United States. Proceedings of the United States National Museum 4(1881): 29–70. 10.5479/si.00963801.4-191.29

[B111] Kamikawa DJ Stevenson DE (2010) New records of *Aldrovandiaoleosa* (Notacanthiformes: Halosauridae) from the eastern North Pacific Ocean. California Fish and Game 96(3): 216–220. https://nrm.dfg.ca.gov/FileHandler.ashx?DocumentID=47311&inline=1

[B112] Kells V Rocha LA Allen LG (2016) A field guide to coastal fishes from Alaska to California. Johns Hopkins University Press, Baltimore, 366 pp. https://jhupbooks.press.jhu.edu/content/field-guide-coastal-fishes-0

[B113] Kline DE Donlou NE Lea RN Lindholm JB Shester GG (2013) Records of the longfin gunnel, *Pholisclemensi*, from California with a southern range extension to Point Lobos, California. Marine Biodiversity Records 6(e10): 1–4. 10.1017/S1755267212001169

[B114] Kotlyar AN (2004) Family Melamphaidae Gill 1893 – bigscales. California Academy of Sciences Annotated Checklists of Fishes 29: 1–11. https://www.calacademy.org/sites/default/files/assets/docs/melamphaidae.pdf

[B115] Knudsen SW Clements KD (2013) Revision of the fish family Kyphosidae (Teleostei: Perciformes). Zootaxa 3751(1): 1–101. 10.11646/zootaxa.3751.1.1 29097648

[B116] Knudsen SW Clements KD (2016) World-wide species distributions in the family Kyphosidae (Teleostei: Perciformes). Molecular Phylogenetics and Evolution 101: 252–266. 10.1016/j.ympev.2016.04.037 27143240

[B117] Last PR Séret B Naylor GJP (2016a) A new species of guitarfish, *Rhinobatosborneensis* sp. nov. with a redefinition of the family-level classification in the order Rhinopristiformes (Chondrichthyes: Batoidea). Zootaxa 4117(4): 451–475. 10.11646/zootaxa.4117.4.1 27395187

[B118] Last PR Stehmann MF Séret B Weigmann S (2016b) Softnose Skates. Family Arhynchobatidae. In: Last PR White WT de Carvalho MR Séret B Stehmann MFW Naylor GJP (Eds) Rays of the World.Cornell University Press, Ithaca, New York, 364–472. 10.1071/9780643109148

[B119] Last PR Weigmann S Yang L (2016c) Changes to the nomenclature of the skates (Chondrichthyes: Rajiformes). In: Last PR Yearsley GK (Eds) Rays of the World: Supplementary Information.CSIRO Special Publication, 11–34. http://www.publish.csiro.au/book/7053/

[B120] Last PR White WT de Carvalho MR Séret B Stehmann MFW Naylor GJP (Eds) (2016d) Rays of the World. Cornell University Press, Ithaca, 800 pp. 10.1071/9780643109148

[B121] Lauth RR (2000) The 1999 Pacific west coast upper continental slope trawl survey of groundfish resources off Washington, Oregon, and California: Estimates of distribution, abundance, and length composition. US Department of Commerce, NOAA Technical Memorandum NMFS-AFSC-115, 287 pp. https://www.afsc.noaa.gov/techmemos/nmfs-afsc-115.htm

[B122] Lauth RR (2001) The 2000 Pacific west coast upper continental slope trawl survey of groundfish resources off Washington, Oregon, and California: Estimates of distribution, abundance, and length composition. US Department of Commerce, NOAA Technical Memorandum NMFS-AFSC-120, 284 pp. https://www.afsc.noaa.gov/techmemos/nmfs-afsc-120.htm

[B123] Lay GT Bennett ET (1839) Fishes. In: Bechey FW (Ed.) The zoology of Captain Beechey's voyage, compiled from the collections and notes made by Captain Beechey, the officers and naturalist of the expedition, during a voyage to the Pacific and Behring's Straits performed in his Majesty’s Ship Blossom, under the command of Captain F.W. Beechey, R.N., F.R.S., &c. &c. in the years 1825, 26, 27, and 28. H.G. Bohn, London, 41–75. https://archive.org/details/zoologyofcaptain00beec

[B124] Lea RN (1972) Southern geographical records for four surfperches, family Embiotocidae, with notes on a population resurgence of the sharpnose seaperch. California Fish and Game 58(1): 27–31. https://www.biodiversitylibrary.org/item/61759

[B125] Lea RN (1983) *Sebastodesatrorubens* Gilbert, l898, a junior synonym of *Sebastesatrovirens* (Jordan and Gilbert, l880), with notes on individual variation in the species. Bulletin Southern California Academy of Sciences 82(3): 147–149. http://biodiversitylibrary.org/page/34407012

[B126] Lea RN (1988) Family Himantolophidae added to the ichthyofauna of the temperate eastern north Pacific. California Fish and Game 74(3): 172–185. https://www.biodiversitylibrary.org/item/72474

[B127] Lea RN (1997) Central California Marine Sport Fish Project: Marine Biodiversity Study and CPFV Sampling Study. California Department of Fish and Game Cruise Report 97-M-9, prepared 24 November 1997, 7 pp.

[B128] Lea RN (2013) Record of the Kamchatka Flounder, *Atheresthesevermanni*, in California waters. Northwestern Naturalist 94(3): 244–246. 10.1898/13-01.1

[B129] Lea RN Karpov KA Quirollo LF (1989) Record of the roughscale sole, *Clidodermaasperrimum*, from northern California with a note on the Pacific lined sole, *Achirusmaxatlanus*. California Fish and Game 75(4): 239–241.

[B130] Lea RN Keating T Van Dykhuizen G Lehtonen PB (1984) Records of goosefishes (family: Lophiidae, genus *Lophiodes*) from Californian waters. California Fish and Game 70(4): 250–251. https://www.biodiversitylibrary.org/item/72410

[B131] Lea RN Rosenblatt RH (1987) Occurrence of the family Notacanthidae (Pisces) from marine waters of California. California Fish and Game 73(1): 51–53. https://www.biodiversitylibrary.org/item/72468

[B132] Lea RN Rosenblatt RH (2000) Observations on fishes associated with the 1997-98 El Niño off California. CalCOFI Report 41: 117–129. http://www.calcofi.org/publications/calcofireports/v41/Vol_41_Lea___Rosenblatt.pdf

[B133] Lea RN Walker HJ Jr (1995) Record of the bigeye trevally, *Caranxsexfasciatus*, and Mexican lookdown, *Selenebrevoorti*, with notes on other carangids from California. California Fish and Game 81(3): 89–95. https://www.wildlife.ca.gov/Publications/Journal/Contents

[B134] Limbaugh C (1955) Fish life in the kelp beds and the effects of kelp harvesting. University of California, Institute of Marine Resources, IMR Reference 55-9: 1–158. https://escholarship.org/uc/item/4w36x6mb

[B135] Lockington WN (1879) On a new genus and species of Scombridae. Proceedings of the Academy of Natural Sciences of Philadelphia 31: 133–136. https://www.biodiversitylibrary.org/part/84801

[B136] Lockington WN (1880) Description of a new chiroid fish, *Myriolepiszonifer*, from Monterey Bay, California. Proceedings of the United States National Museum 3(140): 248–251. 10.5479/si.00963801.140.248

[B137] Lonhart SI (2009) Natural and Climate Change Mediated Invasions. In: Rilov G Crooks JA (Eds) Biological Invasions in Marine Ecosystems.Ecological Studies 204, Springer-Verlag, Berlin/Heidelberg, 57–69. 10.1007/978-3-540-79236-9_3

[B138] Longo GC Bernardi G Lea RN (2018) Taxonomic revisions within Embiotocidae (Teleostei, Perciformes) based on molecular phylogenetics. Zootaxa 4482(3): 591–596. 10.11646/zootaxa.4482.3.10 30313817

[B139] Love MS Mecklenburg CW Mecklenburg TA Thorsteinson LK (2005) Resource Inventory of Marine and Estuarine Fishes of the West Coast and Alaska: A Checklist of North Pacific and Arctic Ocean Species from Baja California to the Alaska–Yukon Border. U. S. Department of the Interior, U. S. Geological Survey, Biological Resources Division, Seattle, Washington, 98104, OCS Study MMS 2005-030 and USGS/NBII 2005-001. http://www.lovelab.id.ucsb.edu/checklist.html.

[B140] Love MS Yoklavich MM Thorsteinson LK (2002) The rockfishes of the Northeast Pacific. University of California Press, Berkeley, California, 405 pp. https://www.ucpress.edu/op.php?isbn=9780520234383

[B141] Lundsten L Johnson SB Cailliet GM DeVogelaere AP Clague DA (2012) Morphological, molecular, and in situ behavioral observations of the rare deep-sea anglerfish *Chaunacopscoloratus* (Garman 1899), order Lophiiformes, in the eastern North Pacific. Deep Sea Research I 68: 46–53. 10.1016/j.dsr.2012.05.012

[B142] Lundsten L McClain CR Barry JP Cailliet GM Clague DA DeVogelaere AP (2009) Ichthyofauna on three seamounts off southern and central California, USA. Marine Ecology Progress Series 389: 223–232. 10.3354/meps08181

[B143] Martinez-Takeshita N Purcell CM Chabot CL Craig MT Paterson CN Hyde JR Allen LG (2015) A tale of three tails: cryptic speciation in a globally distributed marine fish of the genus *Seriola*. Copeia 103(2): 357–368. 10.1643/CI-124-224

[B144] Mecklenburg CW Mecklenburg TA Thorsteinson LK (2002) Fishes of Alaska. American Fisheries Society, Bethesda, Maryland, 1116 pp.

[B145] Meek SE Pierson CJ (1895) Description of a new species of *Gobiesox* from Monterey Bay, California. Proceedings of the California Academy of Sciences (Series 2) 5: 571–572. http://www.biodiversitylibrary.org/page/32283259

[B146] Milkova V Hanke G Gillespie G Fong K Boutillier J Bedard J (2016) Range records for ten species of stomiiform, aulopiform, and myctophiform fishes in British Columbia, Canada. Northwestern Naturalist 97(2): 113–123. 10.1898/NWN15-11.1

[B147] Miller DJ Gotshall D (1965) Ocean Sportfish Catch and Effort from Oregon to Point Arguello, California, July 1, 1957–June 30, 1961. California Department of Fish and Game, Fish Bulletin 130, 135 pp. http://content.cdlib.org/ark:/13030/kt1g5001fm

[B148] Miller DJ Lea RN (1972) Guide to the coastal marine fishes of California. California Department of Fish and Game, Fish Bulletin 157: 1–249. http://content.cdlib.org/ark:/13030/kt896nb2qd

[B149] Mollet HF (2002) Distribution of the pelagic stingray, *Dasyatisviolacea* (Bonaparte, 1832), off California, Central America, and worldwide. Marine and Freshwater Research 53: 525–530. 10.1071/MF02010

[B150] Monterey Bay Aquarium (1999) Natural History of the Monterey Bay National Marine Sanctuary. Monterey Bay Aquarium Foundation, Monterey, 299 pp.

[B151] Monterey County Herald (1997) Unusual catch in local waters: Fishermen take mahi mahi off Point Pinos. [2 Sept 1997; Section B; Michelle Maitre]

[B152] Monterey Herald (2018) ‘Tsunami’ fish shows up in Monterey Bay. [10 Dec 2018; Rodrigo Pérez Ortega] https://www.montereyherald.com/2018/12/10/tsunami-fish-shows-up-in-monterey-bay/

[B153] Morita T (1999) Molecular phylogenetic relationships of the deep-sea fish genus Coryphaenoides (Gadiformes: Macrouridae) based on mitochondrial DNA. Molecular Phylogenetics and Evolution 13(3): 447–454. 10.1006/mpev.1999.0661 10620402

[B154] Moser HG (1996) Melanostomiidae: Scaleless dragonfishes. In: Moser HG (Ed.) The early stages of fishes in the California Current region.California Cooperative Oceanic Fisheries Investigations, Atlas 33, Allen Press, Lawrence, Kansas, 308–319. http://calcofi.org/publications/atlases/CalCOFI_Atlas_33.pdf

[B155] Moser HG Butler JL (1996) Microstomatidae: Argentines and pencilfishes. In: Moser HG (Ed.) The early stages of fishes in the California Current region.California Cooperative Oceanic Fisheries Investigations, Atlas 33, Allen Press, Lawrence, Kansas, 208–215. http://calcofi.org/publications/atlases/CalCOFI_Atlas_33.pdf

[B156] Moyle PB (2002) Inland Fishes of California. University of California Press, Berkeley, California, 502 pp. https://www.ucpress.edu/book.php?isbn=9780520227545

[B157] Nafpaktitis BG Paxton JR (1968) Review of the lanternfish genus *Lampadena* with a description of a new species. Los Angeles County Museum Contributions in Science No. 138, 29 pp. https://www.biodiversitylibrary.org/part/241127

[B158] Nelson JS (2006) Fishes of the World (4^th^ edn). John Wiley & Sons, New York, 624 pp. https://www.wiley.com/en-us/Fishes+of+the+World%2C+4th+Edition-p-9780471250319

[B159] Nelson JS Crossman EJ Espinosa-Pérez H Findley LT Gilbert CR Lea RN Williams JD (2004) Common and scientific names of fishes from the United States, Canada, and Mexico (6^th^ edn). American Fisheries Society, Special Publication 29, Bethesda, 386 pp. https://fisheries.org/bookstore/all-titles/special-publications/x51029xm/

[B160] Nelson JS Grande TC Wilson MVH (2016) Fishes of the World (5^th^ edn). John Wiley & Sons, New Jersey, 752 pp. 10.1002/9781119174844

[B161] Nelson L (1986) A range extension of *Phidianasternsi* (Cockerell, 1901) (Gastropoda: Nudibranchia). The Veliger 29(2): 240. https://www.biodiversitylibrary.org/part/96977

[B162] NOAA Earth System Research Laboratory (2019) El Niño Southern Oscillation (ENSO): ENSO Index Dashboard. https://www.esrl.noaa.gov/psd/enso/dashboard.html [accessed 2016-2019]

[B163] NOAA Fisheries (2016) Species in the Spotlight, Priority Actions: 2016-2020, Central California Coast Coho Salmon *Oncorhynchuskisutch*. Central California Coast Coho 5-Year Action Plan, 17 pp. http://www.nmfs.noaa.gov/stories/2015/07/spotlight_central_ca_cohosalmon.html

[B164] Norris KS (1963) The functions of temperature in the ecology of the percoid fish *Girellanigricans* (Ayres). Ecological Monographs 33(1): 23–62. 10.2307/1948476

[B165] Null J (2019) El Niño and La Niña years and intensities based on Oceanic Niño Index (ONI). https://www.ggweather.com/enso/oni.htm [accessed 2016-2019]

[B166] Orr JW (2012) Two new species of snailfishes of the genus *Careproctus* (Scorpaeniformes: Liparidae) from the Bering Sea and eastern North Pacific Ocean, with a redescription of *Careproctusovigerus*. Copeia 2012(2): 257–265. 10.1643/CI-11-046

[B167] Orr JW Hawkins S (2008) Species of the rougheye rockfish complex: resurrection of *Sebastesmelanostictus* (Matsubara, 1934) and a redescription of *Sebastesaleutianus* (Jordan and Evermann, 1898) (Teleostei: Scorpaeniformes). Fishery Bulletin 106(2): 111–134. https://www.st.nmfs.noaa.gov/spo/FishBull/1062/orr.pdf

[B168] Page LM Espinosa-Pérez H Findley LT Gilbert CR Lea RN Mandrak NE Mayden RL Nelson JS (2013) Common and scientific names of fishes from the United States, Canada, and Mexico (7^th^ edn). American Fisheries Society, Special Publication 34, Bethesda, 384 pp. https://fisheries.org/bookstore/all-titles/special-publications/51034c/

[B169] Paulin CD (1989) Review of the morid genera *Gadella*, *Physiculus*, and *Salilota* (Teleostei: Gadiformes) with descriptions of seven new species. New Zealand Journal Zoology 16: 93–133. 10.1080/03014223.1989.10423706

[B170] Peden AE (1974) Rare fishes including first records of thirteen species from British Columbia. Syesis 7: 47–62. https://publications.royalbcmuseum.bc.ca/product/syesis-vol-7/

[B171] Peden AE Anderson ME (1978) A systematic review of the fish genus *Lycodapus* (Zoarcidae) with descriptions of two new species. Canadian Journal of Zoology 56(9): 1925–1961. 10.1139/z78-262

[B172] Phillips JB (1930a) Wolf fish captured at Monterey. California Fish and Game 16(3): 267–268. https://www.biodiversitylibrary.org/item/70562

[B173] Phillips JB (1930b) Large black sea bass caught in Monterey Bay. California Fish and Game 16(3): 268. https://www.biodiversitylibrary.org/item/70562

[B174] Phillips JB (1931a) Albacore at Monterey. California Fish and Game 17(1): 85. https://www.biodiversitylibrary.org/item/70691

[B175] Phillips JB (1931b) Another wolf fish taken at Monterey. California Fish and Game 17(1): 85–86. https://www.biodiversitylibrary.org/item/70691

[B176] Phillips JB (1932a) Unusually good fishing in and off Monterey Bay. California Fish and Game 18(1): 21–24. https://www.biodiversitylibrary.org/item/70692

[B177] Phillips JB (1932b) Monterey Spanish mackerel landed in Monterey. California Fish and Game 18(1): 99. https://www.biodiversitylibrary.org/item/70692

[B178] Phillips JB (1932c) Wolf-fish taken at Monterey. California Fish and Game 18(1): 99. https://www.biodiversitylibrary.org/item/70692

[B179] Phillips JB (1932d) Ribbon-fish taken at Monterey. California Fish and Game 18(1): 99–100. https://www.biodiversitylibrary.org/item/70692

[B180] Phillips JB (1935) A large northern halibut in Monterey. California Fish and Game 21(3): 262. https://www.biodiversitylibrary.org/item/70693

[B181] Phillips JB (1936) Big-eyed bass taken at Monterey. California Fish and Game 22(1): 48–49. https://archive.org/details/californiafishga22_1936cali

[B182] Phillips JB (1937) Record mackerel taken at Monterey. California Fish and Game 23(4): 337. https://archive.org/details/californiafishga231937cali

[B183] Phillips JB (1940) A California needlefish recorded at Monterey. California Fish and Game 26(3): 289.

[B184] Phillips JB (1942a) Wall-eyed pollock caught in Monterey Bay. California Fish and Game 28(3): 155–156. https://www.biodiversitylibrary.org/item/70701

[B185] Phillips JB (1942b) Tongue Sole in Monterey Bay. California Fish and Game 28(3): 156. https://www.biodiversitylibrary.org/item/70701

[B186] Phillips JB (1943a) Grunion in Monterey Bay. California Fish and Game 29(2): 82. https://www.biodiversitylibrary.org/item/70703

[B187] Phillips JB (1943b) Another wall-eyed pollock at Monterey. California Fish and Game 29(2): 83. https://www.biodiversitylibrary.org/item/70703

[B188] Phillips JB (1945) Two unusual flatfishes from Monterey Bay. California Fish and Game 31(4): 210–211. https://www.biodiversitylibrary.org/item/70831

[B189] Phillips JB (1948) Basking shark fishery revived in California. California Fish and Game 34(1): 11–23. https://www.biodiversitylibrary.org/item/70838

[B190] Phillips JB (1951a) Pacific cod off central California. California Fish and Game 37(3): 351. https://www.biodiversitylibrary.org/item/61379

[B191] Phillips JB (1951b) Round herring off central California. California Fish and Game 37(4): 512. https://www.biodiversitylibrary.org/item/61380

[B192] Phillips JB (1953) Additional Pacific cod taken off central California. California Fish and Game 39(4): 559. https://www.biodiversitylibrary.org/item/61450

[B193] Phillips JB (1954) Another large black sea bass caught in Monterey Bay. California Fish and Game 40(3): 339. https://www.biodiversitylibrary.org/item/61453

[B194] Phillips JB (1957) A review of the rockfishes of California (Family Scorpaenidae). California Department of Fish and Game, Fish Bulletin 104: 1–158. http://content.cdlib.org/ark:/13030/kt729005d8

[B195] Phillips JB (1958) Southerly occurrences of three northern species of fish during 1957, a warmwater year on the California coast. California Fish and Game 44(4): 349–350. https://www.biodiversitylibrary.org/item/61532

[B196] Phillips JB (1961) Range extensions for two California fishes, with a note on a rare fish. California Fish and Game 47(4): 418. https://www.biodiversitylibrary.org/item/61609

[B197] Phillips JB (1963) A fantail sole, *Xystreurysliolepis*, in Monterey Bay. California Fish and Game 49(3): 209. https://www.biodiversitylibrary.org/item/61613

[B198] Phillips JB (1965a) Northern range extension for the zebraperch, *Hermosillaazurea* Jenkins and Evermann. California Fish and Game 51(1): 55–56. https://www.biodiversitylibrary.org/item/61618

[B199] Phillips JB (1965b) Range extension for the bigmouth sole, *Hippoglossinastomata*. California Fish and Game 51(2): 125–126. https://www.biodiversitylibrary.org/item/61619

[B200] Phillips JB (1966) Skilfish, *Erilepiszonifer* (Lockington), in Californian and Pacific Northwest waters. California Fish and Game 52(3): 151–156. https://www.biodiversitylibrary.org/item/61681

[B201] Phillips JB (1967) A longfin sanddab, *Citharichthysxanthostigma*, and a skilfish, *Erilepiszonifer*, taken in Monterey Bay. California Fish and Game 53(4): 297–298. https://www.biodiversitylibrary.org/item/61686

[B202] Prokofiev AM (2014) Swallowers (Chiasmodontidae) of the East Pacific. Journal of Ichthyology 54(9): 631–641. 10.1134/S0032945214060137

[B203] Prokofiev AM Kukuev EI (2009) Systematics and distribution of black swallowers of the genus *Chiasmodon* (Perciformes: Chiasmodontidae). Journal of Ichthyology 49(10): 899–939. 10.1134/S0032945209100063

[B204] Quirollo LF Dinnel PA (1975) Latitudinal range extensions for yellow and spotted snake eels (genus *Opichthus*). California Fish and Game 61(3): 156–157. https://www.biodiversitylibrary.org/item/61827

[B205] Radovich J (1961) Relationships of some marine organisms of the Northeast Pacific to water temperatures particularly during 1957 through 1959. Fish Bulletin 112: 1–61. http://content.cdlib.org/ark:/13030/kt4r29n72m

[B206] Randall JE DiBattista JD (2012) *Etrumeusmakiawa*, a new species of round herring (Clupeidae: Dussumierinae) from the Hawaiian Islands. Pacific Science 66(1): 97–110. 10.2984/66.1.6

[B207] Reichert AN Lundsten L Ebert DA (2016) First North Pacific records of the pointy nosed blue chimaera, Hydrolaguscf.trolli (Chondrichthyes: Chimaeriformes: Chimaeridae). Marine Biodiversity Records 9: 90. 10.1186/s41200-016-0095-5

[B208] Robison BH Reisenbichler KR (2008) *Macropinnamicrostoma* and the paradox of its tubular eyes. Copeia 2008(4): 780–784. 10.1643/CG-07-082

[B209] Russell BC (2003) Synodontidae and Bathysauridae. In: Carpenter KE (Ed.) , The living marine resources of the Western Central Atlantic.Volume 2: Bony fishes part 1 (Acipenseridae to Grammatidae). FAO species identification guide for fishery purposes and American Society of Ichthyologist and Herpetologists Special Publication No. 5, FAO, Rome, 923–932. http://www.fao.org/docrep/009/y4161e/y4161e00.htm

[B210] Sabaj MH (2016) Standard symbolic codes for institutional resource collections in herpetology and ichthyology: an Online Reference. Version 6.5 (16 August 2016). American Society of Ichthyologists and Herpetologists, Washington, DC. http://www.asih.org/resources/standard-symbolic-codes-institutional-resource-collections-herpetology-ichthyology

[B211] Sandknop EM Watson W (1996) Melamphaidae: Bigscales. In: Moser HG (Ed.) The early stages of fishes in the California Current region.California Cooperative Oceanic Fisheries Investigations, Atlas 33, Allen Press, Lawrence, Kansas, 692–711. http://calcofi.org/publications/atlases/CalCOFI_Atlas_33.pdf

[B212] Sato T Nakabo T (2002) Paraulopidae and *Paraulopus*, a new family and genus of aulopiform fishes with revised relationships within the order. Ichthyological Research 49(1): 25–46. 10.1007/s102280200004

[B213] Scofield NB (1916) The humpback and dog salmon taken in San Lorenzo River. California Fish and Game 2: 41. https://www.biodiversitylibrary.org/item/70694

[B214] Scofield NB (1917) Basking Shark taken in Monterey Bay. California Fish and Game 3(3): 137. https://www.biodiversitylibrary.org/item/63626

[B215] Séret B Last PR Naylor GJP (2016) Guitarfishes. Family Rhinobatidae. In: Last PR White WT de Carvalho MR Séret B Stehmann MFW Naylor GJP (Eds) Rays of the World.Cornell University Press, Ithaca, 77–109. 10.1071/9780643109148

[B216] Sheiko BA Mecklenburg CW (2004) Family Agonidae Swainson 1839 - poachers. California Academy of Sciences Annotated Checklists of Fishes No. 30: 1–27. https://www.calacademy.org/sites/default/files/assets/docs/agonidae.pdf

[B217] SIMoN (Sanctuary Integrated Monitoring Network) (2018) Photo Library. http://www.sanctuarysimon.org/photos/index.php [accessed 8 Aug 2018]

[B218] SIMoN (Sanctuary Integrated Monitoring Network) (2019) Species Database. https://sanctuarysimon.org/dbtools/species-database/

[B219] Skogsberg T (1939) The fishes of the family Sciaenidae (croakers) of California. Fish Bulletin 54: 1–62. http://content.cdlib.org/ark:/13030/kt0d5n97jz

[B220] Smith JG Gotshall DW (1967) Northerly occurrences of kelp bass, *Paralabraxclathratus* (Girard), since 1959. California Fish and Game 53(1): 63. https://www.biodiversitylibrary.org/item/61683

[B221] Snyder JO (1913) Notes on *Ranzaniamakua* Jenkins and other species of fishes of rare occurrence on the California coast. Proceedings of the United States National Museum 44(1961): 455–460. 10.5479/si.00963801.44-1961.455

[B222] Spratt JD (1981) California grunion, Leuresthestenuis, spawn in Monterey Bay, California. California Fish and Game 67(2): 134. https://www.biodiversitylibrary.org/item/72400

[B223] Springer VG Anderson ME (1997) Catalog of type specimens of recent fishes in the National Museum of Natural History, Smithsonian Institution, 8: Suborder Zoarcoidei (Anarhichadidae, Bathymasteridae, Pholidae, Ptilichthyidae, Scytalinidae, Stichaeidae, Zoarcidae). Smithsonian Contributions to Zoology No. 589, 1–27. 10.5479/si.00810282.589

[B224] Starks EC (1919) Rare fish from Monterey Bay. California Fish and Game 5(1): 43. https://www.biodiversitylibrary.org/item/53563

[B225] Starks EC (1921) The Basking Shark at Monterey. California Fish and Game 7(3): 178. https://www.biodiversitylibrary.org/item/72466

[B226] Starnes WC (1988) Revision, phylogeny and biogeographic comments on the circumtropical marine percoid fish family Priacanthidae. Bulletin of Marine Science 43(2): 117–203. http://www.ingentaconnect.com/content/umrsmas/bullmar/1988/00000043/00000002/art00001

[B227] Starr RM Cope JM Kerr LA (2002) Trends in fisheries and fishery resources associated with the Monterey Bay National Marine Sanctuary From 1981–2000. Publication No. T-046, California Sea Grant College Program, La Jolla, 169 pp. https://montereybay.noaa.gov/research/techreports/trstarr2002b.html

[B228] Stein DL Drazen JC Schlining KL Barry JP Kuhnz L (2006) Snailfishes of the central California coast: video, photographic and morphological observations. Journal of Fish Biology 69(4): 970–986. 10.1111/j.1095-8649.2006.01167.x

[B229] Stevens DE Kohlhorst DW (2001) Striped Bass. In: Leet WS Dewees CM Klingbeil R Larson EJ (Eds) California’s Living Marine Resources: A Status Report.University of California, 460–464. https://www.wildlife.ca.gov/Conservation/Marine/Status/2001

[B230] Stevenson DE Matarese AC (2005) The ronquils: a review of the North Pacific fish family Bathymasteridae (Actinopterygii: Perciformes: Zoarcoidei). Proceedings of the Biological Society of Washington 118(2): 367–406. 10.2988/0006-324X(2005)118[367:TRAROT]2.0.CO;2

[B231] Ta N Miller JA Chapman JW Pleus AE Calvanese T Miller-Morgan T Burke J Carlton JT (2018) The Western Pacific barred knifejaw, *Oplegnathusfasciatus* (Temminck & Schlegel, 1844) (Pisces: Oplegnathidae), arriving with tsunami debris on the Pacific coast of North America. Aquatic Invastions 13(1): 179–186. 10.3391/ai.2018.13.1.14

[B232] Thompson WF (1920) The grunion at Monterey. California Fish and Game 6(3): 130. https://www.biodiversitylibrary.org/item/53566

[B233] Thompson WF (1921a) The basking shark at Monterey. California Fish and Game 7(3): 178. https://www.biodiversitylibrary.org/item/72466

[B234] Thompson WF (1921b) A rag fish at Monterey. California Fish and Game 7(3): 179. https://www.biodiversitylibrary.org/item/72466

[B235] Underkoffler KE Luers MA Hyde JR Craig MT (2018) A taxonomic review of *Lamprisguttatus* (Brünnich 1788) (Lampridiformes; Lampridae) with descriptions of three new species. Zootaxa 4413(3): 551–565. 10.11646/zootaxa.4413.3.9 29690102

[B236] Varoujean DH (1972) The reoccurrence of the California scorpionfish, *Scorpaenaguttata* Girard, in Monterey Bay. California Fish and Game 58(3): 238–239. https://www.biodiversitylibrary.org/item/61760

[B237] Voskoboinikova OS Nazarkin MV (2017) Osteology of the Southern Ocean daggertooth (*Anotopterusvorax*) and status of the *Anotopterus* genus in the Aulopiformes order. Journal of Ichthyology 57(1): 10–19. 10.1134/S0032945217010131

[B238] Wallace M Ojerholm EW Scheiff AJ Kinziger AP (2015) First record of striped mullet (*Mugilcephalus*) in Humboldt Bay, California. California Fish and Game 101(4): 286–288. https://nrm.dfg.ca.gov/FileHandler.ashx?DocumentID=113248&inline

[B239] Weigmann S (2016) Annotated checklist of the living sharks, batoids and chimaeras (Chondrichthyes) of the world, with a focus on biogeographical diversity. Journal of Fish Biology 88(3): 837–1281. 10.1111/jfb.12874 26860638

[B240] Weinberg KL Wilkins ME Shaw FR Zimmerman M (2002) The 2001 Pacific west coast bottom trawl survey of groundfish resources: Estimates of distribution, abundance, and length and age composition. US Department of Commerce, NOAA Technical Memorandum NMFS-AFSC-128, 140 pp. https://www.afsc.noaa.gov/techmemos/nmfs-afsc-128.htm

[B241] Wilimovsky NJ Wilson DE (1979) A new species of Agonidae, *Agonomalusmozinoi*, from the west coast of North America. Syesis 11: 73–79. https://publications.royalbcmuseum.bc.ca/product/syesis-vol-11/

[B242] Wilson EO (2014) The Meaning of Human Existence. Liveright Publishing Corporation, New York, 207 pp.

[B243] Wilson RR Jr (1994) Interrelationships of the subgenera of *Coryphaenoides* (Gadiformes: Macrouridae): Comparison of protein electrophoresis and peptide mapping. Copeia 1994(1): 42–50. 10.2307/1446669

[B244] Wilson RR Jr Attia P (2003) Interrelationships of the subgenera of *Coryphaenoides* (Teleostei: Gadiformes: Macrouridae): synthesis of allozyme, peptide mapping, and DNA sequence data. Molecular Phylogenetics and Evolution 27: 343–347. 10.1016/S1055-7903(02)00419-0 12695096

[B245] Wisner RL (1976) The taxonomy and distribution of lanternfishes (Family Myctophidae) of the Eastern Pacific Ocean. Navy Ocean Research and Development Activity Report No. 3, Bay St. Louis, Mississippi, 229 pp. 10.21236/ADA041654

[B246] Wolter K Timlin MS (2011) El Niño/Southern Oscillation behaviour since 1871 as diagnosed in an extended multivariate ENSO index (MEI.ext). International Journal of Climatology 31(7): 1074–1087. 10.1002/joc.2336

[B247] Yoklavich MM Cailliet GM Barry JP Ambrose DA Antrim BS (1991) Temporal and spatial patterns in abundance and diversity of fish assemblages in Elkhorn Slough, California. Estuaries 14(4): 465–480. 10.2307/1352270

[B248] Yoklavich MM Cailliet GM Oxman DS Barry JP Lindquist DC (2002) Fishes. In: Caffrey JM Brown M Tyler B (Eds) Changes in a California Estuary: An Ecosystem Profile of Elkhorn Slough.Monterey Bay Aquarium Foundation. Monterey, California, 163–185. http://digital.mlml.calstate.edu/islandora/object/islandora%3A2438

